# Diphosphoryl‐functionalized Polyoxometalates: Structurally and Electronically Tunable Hybrid Molecular Materials

**DOI:** 10.1002/anie.202302446

**Published:** 2023-04-26

**Authors:** Sharad S. Amin, Kieran D. Jones, Alexander J. Kibler, Heather A. Damian, Jamie M. Cameron, Kevin S. Butler, Stephen P. Argent, Max Winslow, David Robinson, Nicholas J. Mitchell, Hon Wai Lam, Graham N. Newton

**Affiliations:** ^1^ The GlaxoSmithKline Carbon Neutral Laboratories for Sustainable Chemistry University of Nottingham Jubilee Campus Triumph Road Nottingham NG7 2TU UK; ^2^ School of Chemistry University of Nottingham University Park Nottingham NG7 2RD UK; ^3^ Department of Chemistry and Forensics School of Science and Technology Nottingham Trent University Nottingham NG11 8NS UK

**Keywords:** Clusters, Hybrid Materials, Organophosphorus, Polyoxometalates, Redox Properties

## Abstract

Herein, we report the synthesis and characterization of a new class of hybrid Wells–Dawson polyoxometalate (POM) containing a diphosphoryl group (P_2_O_6_X) of the general formula [P_2_W_17_O_57_(P_2_O_6_X)]^6−^ (X=O, NH, or CR^1^R^2^). Modifying the bridging unit X was found to impact the redox potentials of the POM. The ease with which a range of α‐functionalized diphosphonic acids (X=CR^1^R^2^) can be prepared provides possibilities to access diverse functionalized hybrid POMs. Compared to existing phosphonate hybrid Wells–Dawson POMs, diphosphoryl‐substituted POMs offer a wider tunable redox window and enhanced hydrolytic stability. This study provides a basis for the rational design and synthesis of next‐generation hybrid Wells–Dawson POMs.

## Graphical abstract

The synthesis and characterization of a new class of hybrid diphosphoryl Wells–Dawson polyoxometalate (POM) is described. The covalent tethering of diphosphoryl compounds to the inorganic cluster gives redox‐active materials with enhanced hydrolytic stability compared to the corresponding phosphonate analogues. These readily modifiable precursors provide a platform for structural and electronic modulation of the POM, enabling the rational design and synthesis of next‐generation hybrid Wells–Dawson POMs.

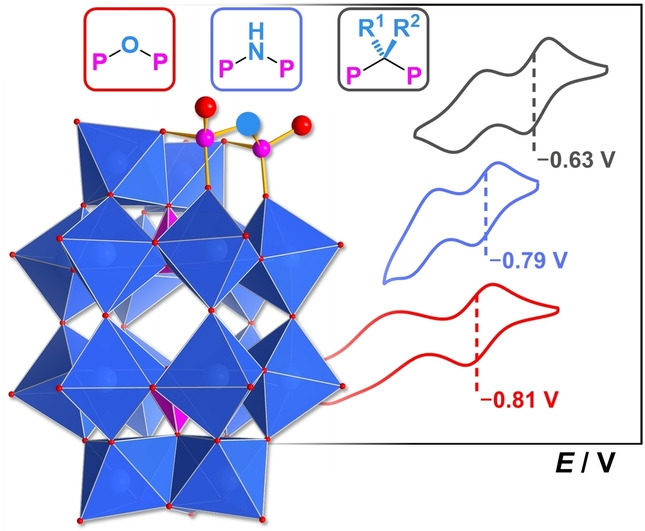

Polyoxometalates (POMs) are polyanionic molecular metal oxide clusters with diverse structures, versatile chemical properties, and a capacity for reversible multi‐electron redox chemistry.^[1]^ These properties make them attractive components for incorporation into a range of hybrid, soft, and nanoscale materials.^[2]^ In some cases, they can also be covalently modified with organic fragments to yield so‐called organic–inorganic hybrid POMs.^[3]^ These systems can be rationally designed to incorporate desirable chemical functionalities, and to exhibit varying structural topographies based on the properties of their constituent organic and inorganic components.

Organophosphorus functionalization of POMs allows the tuning of the electronic structure and photochemical behavior of the metal oxide based on the electronic properties of the appended organic group.^[4]^ This functionalization approach has been demonstrated with the Keggin and Wells–Dawson cluster archetypes, which typically yield hybrid POM clusters with two identical phosphoryl moieties tethered to a single lacunary site on the inorganic POM core, although functionalization with two different phosphoryl groups was recently demonstrated for the first time.^[5]^

The requirement to attach two groups to fill the lacunary site of Wells–Dawson POMs can be a limitation. The use of two equivalents of one organophosphonate in the condensation reaction results in a hybrid POM with two identical appendages, which may not always be desired, whereas the use of two different groups is synthetically challenging because of the potential to create mixtures of three possible POMs.^[5a]^ The development of strategies to address this limitation, while retaining the benefits offered by organophosphorus functionalization, would be highly valuable to increase the range of accessible hybrid POMs.

One approach to address this limitation is to use a single unit that will bridge between all four tungsten centers that define the corners of the lacunary site. We considered diphosphoryl compounds as promising candidates in view of their accessibility and ease of synthesis.^[6]^ Pyrophosphate, medronic and etidronic acid derivatives have been used for the hybridization of metal oxides, leading to atypical W^VI^ or Mo^VI^ oxide clusters of various properties and functions;^[7]^ however, their incorporation into the Wells–Dawson framework has yet to be demonstrated.

Herein, we describe the synthesis and electronic properties of diphosphoryl hybrid Wells–Dawson phosphotungstates (Figure 1) of the general formula [P_2_W_17_O_57_(P_2_O_6_X)]^6−^ (X=O, NH, or CR^1^R^2^). To the best of our knowledge, these are the first examples of pyrophosphate and diphosphonates covalently attached at the lacunary site of a Wells–Dawson POM, as well as the only example of imidodiphosphate incorporation with any POM archetype. This study demonstrates diphosphoryl compounds are versatile building blocks for the rational design of functionalized POM‐based molecular materials.

**Figure 1:**
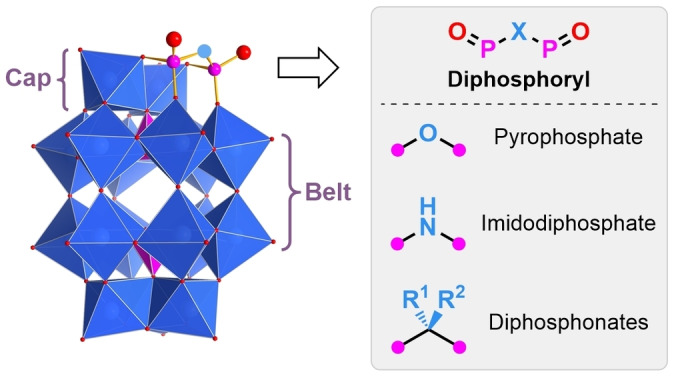
Hybrid diphosphoryl Wells–Dawson phosphotungstates. Blue polyhedra=(WO_6_), pink polyhedra=(PO_4_), red spheres=O, pink spheres=P.

K_6_[P_2_W_17_O_57_(P_2_O_7_)] (**2**), K_6_[P_2_W_17_O_57_(P_2_O_6_NH)] (**3**), and K_6_[P_2_W_17_O_57_(P_2_O_6_CH_2_)] (**4**) were prepared (Scheme 1A) by condensation of their respective commercially available diphosphoryl components (tetrasodium pyrophosphate, tetrasodium imidodiphosphate, and methylene diphosphonic acid) with the lacunary precursor K_10_[P_2_W_17_O_61_] (**1**) in DMF and 12 M aqueous HCl (8–10 equiv). Hybrid POMs **2**–**4** were characterized by electrospray ionization mass spectrometry, NMR, and IR spectroscopies (see the Supporting Information for details). The ^31^P NMR spectra showed three distinctive peaks for each hybrid: two resonances for the templating phosphates, and one for the diphosphoryl group, the chemical shift of which decreased across the series [14.64 (P−C−P), 5.44 (P−N−P), and −18.07 (P−O−P) ppm].

**Scheme 1:**
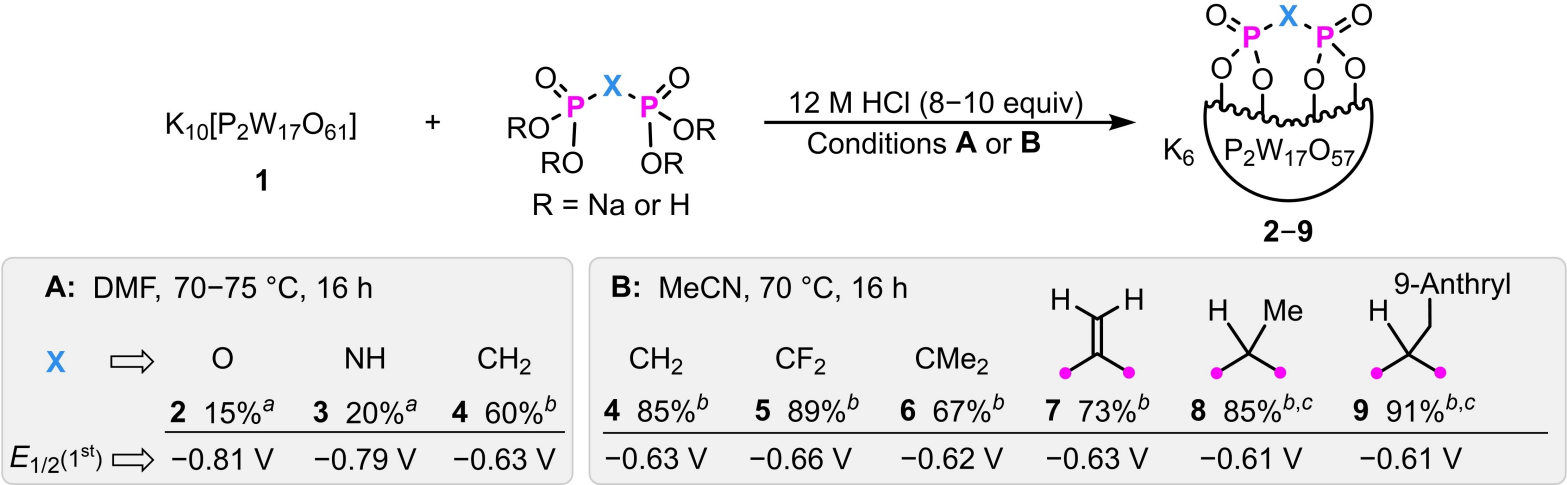
Synthesis of diphosphoryl hybrid Wells–Dawson phosphotungstates. Isolated yields are reported. *E*_1/2_ for the first redox couple is shown, calculated from cyclic voltammetry of 1 mM of compound in DMF with 0.1 M ^*n*^Bu_4_NPF_6_ supporting electrolyte vs. Fc^+^|Fc redox couple. ^*a*^Tetrasodium diphosphoryl salts (R=Na) were used. ^*b*^Diphosphonic acids (R=H) were used. ^*c*^Isolated as a 1 : 1 mixture of inseparable diastereomers as determined by ^1^H NMR spectroscopy.

Compound **3** and the tetrabutylammonium (^*n*^Bu_4_N) salt of [P_2_W_17_O_57_(P_2_O_6_CH_2_)]^6−^ (**4′**) were isolated as crystalline solids. Single crystal X‐ray diffraction analysis of **3** and **4′** showed them to be in the triclinic *P* $\bar{1}$ and orthorhombic *P*2_1_2_1_2_1_ space groups, respectively (see the Supporting Information for details), and in both cases, a single diphosphoryl group occupies the lacunary pocket of the precursor **1**.

Changing the solvent from DMF to MeCN (Scheme 1B) improved the efficiency of the condensation of diphosphonic acids, giving **4** in excellent yield (85 %). This procedure could also be conducted on a gram scale (84 % yield), demonstrating the accessibility of these materials. The Streb group has recently reported mechanistic insight into these condensation reactions which might be useful in further optimizing their efficiency.^[8]^ Replacement of the methylene bridge of compound **4** with more highly substituted derivatives enables the preparation of additional functionalized hybrid POMs and the opportunity to further modulate physical properties. The use of diphosphonic acid precursors with two fluorine or two methyl groups at the central carbon gave the corresponding hybrid POMs **5** (X=CF_2_) and **6** (X=CMe_2_) in 89 % and 67 % yield, respectively. Encouragingly, alkenyl functionality was also tolerated, as shown by the formation of **7** (X=C=CH_2_) in good yield (73 %). Condensation of **1** with a diphosphonic acid containing one methyl group at the central carbon gave **8** (X=CHMe) as a 1 : 1 mixture of inseparable diastereomers. Replacement of the methyl group with a (9‐anthracenyl)methyl moiety gave **9** [X=CHCH_2_(9‐anthryl)] in excellent yield (91 %), albeit as a 1 : 1 mixture of inseparable diastereomers, suggesting that the diastereomeric ratio is not readily affected by steric factors.

The two groups attached to the bridging carbon atom of the diphosphonate of POMs **4**–**6**, **8**, and **9** are diastereotopic, an inherent structural feature resulting from one group pointing towards the cap and the other pointing away from the cap (Figure 1). By ^1^H NMR spectroscopy (500 MHz), these inequivalent protons in **4** appear as a complex multiplet (Figure 2, left), which was simplified by ^31^P decoupling experiments (Figure 2, right) to show two doublets (^2^ *J*_HH_=−15.1 Hz), characteristic of geminal protons. The observed second order coupling is the result of two overlapping doublet of triplets which originate from geminal coupling partnered with ^1^H−^31^P coupling. Inspection of the occupied molecular orbitals of **4**, as calculated using density functional theory (see the Supporting Information for details), found the HOMO‐1 to be the highest occupied orbital with appreciable electron density at the disphosphoryl bridge. The calculated electron densities at each of the two diastereotopic hydrogen atoms are appreciably different, which is consistent with the 0.12 ppm difference in their chemical shifts in the ^1^H NMR spectrum.

**Figure 2:**
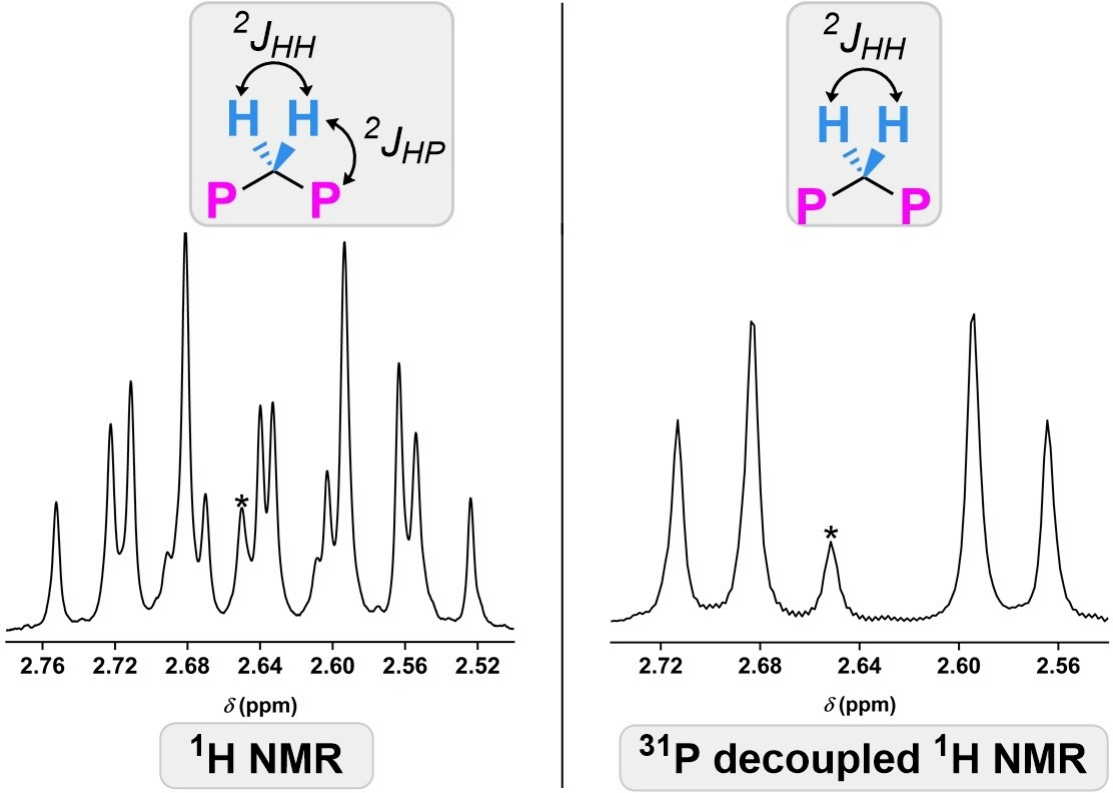
Left: ^1^H NMR spectrum of POM **4** in D_2_O (500 MHz) showing the resonances of the diastereotopic methylene protons. Right: ^1^H NMR spectrum with ^31^P decoupling of POM **4** showing geminal proton coupling. *Unidentified impurity.

Despite the absence of alkylammonium cations or bulky organic groups that would increase their solubility in organic solvents, POMs **2**–**9** are readily soluble in acetone and MeCN. The potassium salts of these clusters are also water‐soluble; however, previous studies have shown monophosphonate‐hybridized POMs undergo hydrolytic cleavage of the phosphoryl group from the POM cluster, which limits their application in aqueous systems.^[9]^ The hydrolytic stabilities of materials **2**–**4** were tested alongside the known phenylphosphonate hybrid POM K_6_[P_2_W_17_O_57_(P_2_O_6_Ph_2_)] by monitoring solutions of these compounds in D_2_O at room temperature by ^31^P NMR spectroscopy (see the Supporting Information for details). The phenylphosphonate‐functionalized POM showed short term hydrolytic stability (24 h), which is in agreement with the literature;^[9b]^ however, formation of PhPO_3_D_2_, [P_2_W_17_O_61_]^10−^ and [P_2_W_18_O_62_]^6−^ was observed after 4 days. The oxo‐ and imido‐ bridges of **2** and **3** were susceptible to hydrolytic cleavage, with [P_2_W_18_O_62_]^6−^, phosphate, and phosphoramidate by‐products evident after 4 and 1 days, respectively. In comparison, the methylene‐bridged diphosphoryl cluster **4** showed excellent aqueous‐stability with no notable change in spectra after 28 days. The robustness of **4** can be attributed to the four bonds between the POM and the capping group and the stability of the P−C bonds.

Cyclic voltammetry (CV) was conducted in DMF (0.1 M ^*n*^Bu_4_NPF_6_) to assess the electronic structure of compounds **2**–**9**. All were found to exhibit two quasi‐reversible processes, all of which are positively shifted with respect to the first two redox events of the plenary parent species K_6_[P_2_W_18_O_62_] (see the Supporting Information for details). This observation is ascribed to the electron‐withdrawing nature of phosphoryl groups, leading to reduced electron density on the W^VI^ centers, which in turn lowers the LUMO energy.[^9b^, ^10^]

Altering the bridging atom has a significant impact on the *E*_1/2_ value of the first redox process (Figure 3). POM **4** displays the most positive redox process (*E*_1/2_=−0.63 V vs. Fc^+^|Fc), followed by **3** (*E*_1/2_=−0.79 V vs. Fc^+^|Fc) and then **2** (*E*_1/2_=−0.81 V vs. Fc^+^|Fc) which suggests that in these systems pyrophosphate and imidodiphosphate are less electron‐withdrawing than methylene diphosphonate. Based on the relative reduction potentials, **4** is the most readily reduced, and therefore possesses the lowest LUMO energy. This is corroborated by DFT calculations of the potential (see the Supporting Information for details), where the potential of **4** is (−0.67 V) followed by **3** (−0.81 V) and finally **2** (−0.96 V).^11^

**Figure 3:**
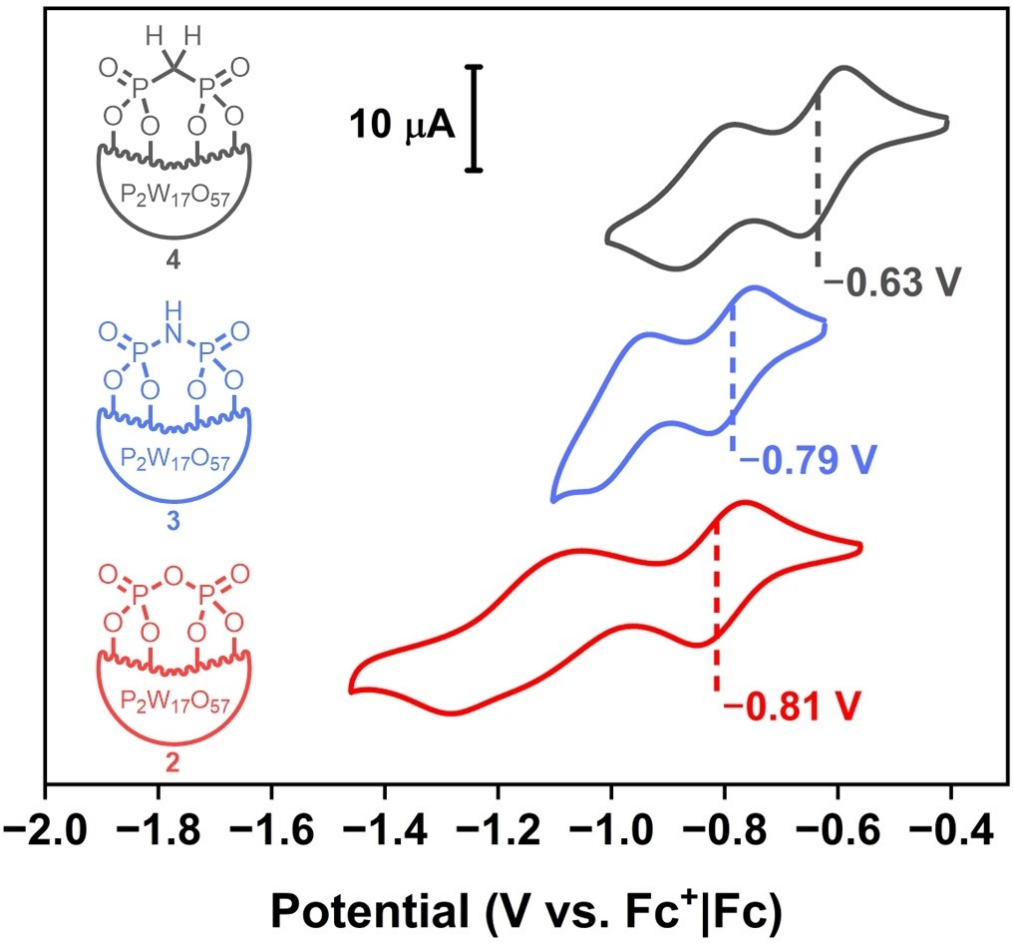
A cyclic voltammogram of 1 mM of compounds of **2**, **3**, and **4** in DMF with 0.1 M ^*n*^Bu_4_NPF_6_ supporting electrolyte at 100 mV s^−1^ vs. Fc^+^|Fc redox couple. Glassy carbon working electrode (*d*=3 mm), Pt wire counter electrode, and a Ag wire pseudo reference were used. The *E*_1/2_ potentials are highlighted for the first redox process.

With POM **4** displaying excellent hydrolytic stability and the lowest LUMO energy, diphosphonate‐hybridized POMs are valuable targets for further modulation of the redox properties of the Wells–Dawson POM. Difluoromethylenediphosphonate is known to be an isopolar analogue of pyrophosphate,^[12]^ and its incorporation into POM **5** (*E*_1/2_=−0.66 V vs. Fc^+^|Fc) mimicked the behavior of **2** with a negative shift in the first redox event with respect to **4** (−30 mV), albeit to a lesser degree. Alkyl substitution at the carbon bridge resulted in a marginally shifted *E*_1/2_ of the first redox event as seen for the α‐monosubstituted diphosphonates **8** and **9** (*E*_1/2_=−0.61 V vs. Fc^+^|Fc), as well as the α,α‐disubstituted disphosphonate **6** (*E*_1/2_=−0.62 V vs. Fc^+^|Fc). The increased s‐character at the carbon bridge of **7** (*E*_1/2_=−0.63 V vs. Fc^+^|Fc) did not notably shift the first redox potential in relation to **4**. Considering the observed redox properties of diphosphoryl‐hybridized POMs **2**–**9**, LUMO modulation appears heavily dependent on the electronic character of the bridging atom.

Phosphonate‐functionalized Wells–Dawson POMs have a comparatively narrower window of redox tunability than diphosphoryl POMs (**2**–**9**). The first redox event of the phenylphosphonate hybrid POM K_6_[P_2_W_17_O_57_(P_2_O_6_Ph_2_)] appears at −0.76 V vs. Fc^+^|Fc and has been shown to be shifted up to 30–40 mV with appropriate modification of the phosphonate.[^9b^, ^13^] In contrast, the described diphosphoryl hybrids offer a wider redox window of approximately 200 mv.

In conclusion, we have demonstrated that commercial or readily prepared diphosphoryl compounds can be covalently bound to the lacunary site of the Wells–Dawson phosphotungstate. The diphosphonic acid condensation is robust, scalable, and utilizes readily modifiable synthetic precursors that enables access to diverse hybrid POMs. Partnered with improved hydrolytic stability over monophosphonate functionalized clusters and readily tunable redox properties, this new class of hybrid diphosphoryl POM may lead to the next generation of designer covalently‐tethered hybrid POM materials.

## Conflict of interest

The authors declare no conflict of interest.

## Supporting information

As a service to our authors and readers, this journal provides supporting information supplied by the authors. Such materials are peer reviewed and may be re‐organized for online delivery, but are not copy‐edited or typeset. Technical support issues arising from supporting information (other than missing files) should be addressed to the authors.

Supporting Information

Supporting Information

## Data Availability

The data that support the findings of this study are available in the Supporting Information of this article.
