# Supplementary material for: Diphosphoryl‐functionalized Polyoxometalates: Structurally and Electronically Tunable Hybrid Molecular Materials
Source: Angew Chem Int Ed Engl. 2023 Apr 26;62(23):e202302446. doi: 10.1002/anie.202302446 (PMC10952223; doi:10.1002/anie.202302446)
Supplement: Supplementary file 1 — Supporting Information [file ANIE-62-0-s001.pdf]

Angewandte Chemie
Eine Zeitschrift der Gesellschaft Deutscher Chemiker

Supporting Information

Diphosphoryl-functionalized Polyoxometalates: Structurally and Electronically Tunable Hybrid Molecular Materials

S. S. Amin, K. D. Jones, A. J. Kibler, H. A. Damian, J. M. Cameron, K. S. Butler, S. P. Argent, M. Winslow, D. Robinson, N. J. Mitchell, H. W. Lam, G. N. Newton**

Contents

1.0 General experimental 3

1.1 Chemistry general protocols 3

1.2 General remarks on NMR spectroscopy 4

2.0 Experimental Procedures & Compound Characterisation 5

2.1 Preparation & characterisation 5

$K_6[P_2W_{17}O_{57}(P_2O_7)]$ (2) 6

$K_6[P_2W_{17}O_{57}(P_2O_6NH)]$ (3) 7

$K_6[P_2W_{17}O_{57}(P_2O_6CH_2)]$ (4) 8

$K_6[P_2W_{17}O_{57}(P_2O_6CF_2]$ (5) 10

Propane-2,2-diyldiphosphonic acid (S7) 12

$K_6[P_2W_{17}O_{57}(P_2O_6C(CH_3)_2)]$ (6) 13

$K_6[P_2W_{17}O_{57}(P_2O_6CCH_2)]$ (7) 14

$K_6[P_2W_{17}O_{57}(P_2O_6C(H)CH_3)]$ (8) 15

Tetraethyl (2-(anthracen-9-yl)ethane-1,1-diyl)bis(phosphonate) (S8) 16

(2-(Anthracen-9-yl)ethane-1,1-diyl)diphosphonic acid (S9) 17

$K_6[P_2W_{17}O_{57}(P_2O_6C(H)CH_2$9-anthryl)] (9) 18

2.2 Hydrolytic stability experiments of S6, 2, 3, and 4 19

2.3 Cyclic voltammetry studies 23

2.3 Computational details 25

3.0 Appendix 26

3.1 NMR Spectra 26

3.2 Crystallography 48

4.0 References 52

1.0 General experimental

1.1 Chemistry general protocols

Starting materials and reagents were available from Thermo Fisher Scientific, Sigma Aldrich, Alfa Aesar, and used without further purification unless otherwise stated.

Anhydrous solvents were distilled over the appropriate drying agents according to Amarego and Chai,[1] acquired from a PureSolv MD 5 Solvent Purification System (Inert corp.), or purchased over molecular sieves and degassed (argon) prior to use. All molarity (M) and normality (N) solutions are aqueous unless stated otherwise. All stirred reactions were fitted with a polytetrafluoroethylene (PTFE) coated magnetic stirring bar and magnetically stirred using a hot plate magnetic stirrer unless otherwise stated.

Thin layer chromatography (TLC) was performed on Merck DF-Alufoilien 60F254 0.2 mm precoated plates and compounds were visualized by exposure to UV light. Chromatography was performed using a CombiFlash Nextgen 100 (Teledyne ISCO) fitted with Redisep® silver silica gel disposable flash columns.

^{1}H, ^{13}C, ^{19}F, and ^{31}P Nuclear Magnetic Resonance (NMR) spectra were acquired on a Bruker AV(III)400 HD or a Bruker AV(III)500 HD fitted with a 5 mm BBFO and 5 mm prodigy BBO probes, respectively. For ^{1}H with decoupled ^{31}P NMR experiments, an inverse-gated sequence with sw 21 ppm and 16000 points to give an AQ of 0.78 s was used. Processing of all NMR data was conducted with Bruker TopSpin or Mestrelab Research Mnova software suites.

Electrospray ionisation mass spectrometry (ESI-MS) was performed on a Bruker MicroTOF spectrometer operating in negative mode. Samples were prepared for analysis by dissolving ca. 1 mg of the solid compound in 1 mL of HPLC grade acetonitrile. 50 µL of this stock solution was then introduced to the spectrometer through an auto-sampler by mixing into a stream of 30:70 H_2O:MeOH. All data was subsequently analysed using the Bruker DataAnalysis software suite. Modified instrument parameters were used for the detection of high molecular weight polyoxometalate anions.

Attenuated Total Reflection Fourier-transform Infra-red spectroscopy (ATR-FTIR) was recorded on a Bruker Tensor 27 spectrometer equipped with a Pike GladiATR module with a diamond crystal.

Cyclic voltammetry (CV) was performed on a CHI450c, CHI600E, or CHI700D workstation for solution state measurements. A standard three electrode set-up was employed with a glassy carbon working electrode (d = 3 mm), Pt wire counter electrode, and a Ag wire pseudo reference. CV was carried out on 1 mM of analyte in deoxygenated anhydrous DMF (5 mL) with 0.1 M $^{n}Bu_4NPF_6$ supporting electrolyte, scan rate 100 mVs^{-1}. Redox potentials are referenced to the ferrocenium (Fc^{+})|ferrocene (Fe) couple used as an internal standard.

Single crystal X-ray diffraction data were collected on Oxford Diffraction SuperNova diffractometers fitted with TitanS2 CCD area detector (mirror-monochromated Cu-Kα radiation source; λ = 1.54184 Å), and Atlas CCD area detector (mirror-monochromated Mo-Kα radiation source; λ = 0.71073 Å; ω scans). Full crystallographic data and specific details can be found in the appendices.

The following materials were prepared according to literature procedures: $K_{10}[P_2W_{17}O_{61}]$ (**1**),[2] $K_6[P_2W_{18}O_{62}]$ (**S1**),[2] (difluoromethylene)diphosphonic acid (**S2**),[3] tetraethyl propane-2,2-diylbis(phosphonate) (**S3**),[4] vinylidene-1,1-diphosphonic acid (**S4**),[5] ethane-1,1-diyldiphosphonic acid (**S5**),[5] and $K_6[P_2W_{17}O_{57}(P_2O_6Ph_2)]$ (**S6**).[6]

1.2 General remarks on NMR spectroscopy

All signals δ are reported in parts per million (ppm). Where appropriate, chemical shifts in ^{1}H and ^{13}C spectra were referenced to the residual (partially) non-deuterated solvent according to Fulmer *et al*.[7] ^{19}F and ^{31}P NMR spectra were referenced through the solvent lock (^{2}H) signal according to the IUPAC-recommended secondary referencing method following Bruker protocols.[8]

Data are reported as follows where appropriate: chemical shift, multiplicity, coupling constant, integration, and assignment. Multiplicity is abbreviated as follows: s (singlet), d (doublet), t (triplet), dt (doublet of triplets), tt (triplet of triplets), and td (triplet of doublets). Broad signals are labelled with the prefix "br" (*e.g.* br s = broad singlet). Apparent multiplets are labelled with the prefix "app." and describe multiplicity that reflects an ordered system but does not display coupling constants (*e.g.* app. t = apparent triplet). Complex signals of high order or due to signal overlap, that also display no apparent multiplicity, are reported with m (multiplet). Coupling constants *J* are reported in hertz (Hz). Some structural assignments were aided by APT, DEPT, COSY, HSQC, and HMBC spectroscopies.

2.0 Experimental Procedures & Compound Characterisation

2.1 Preparation & characterisation

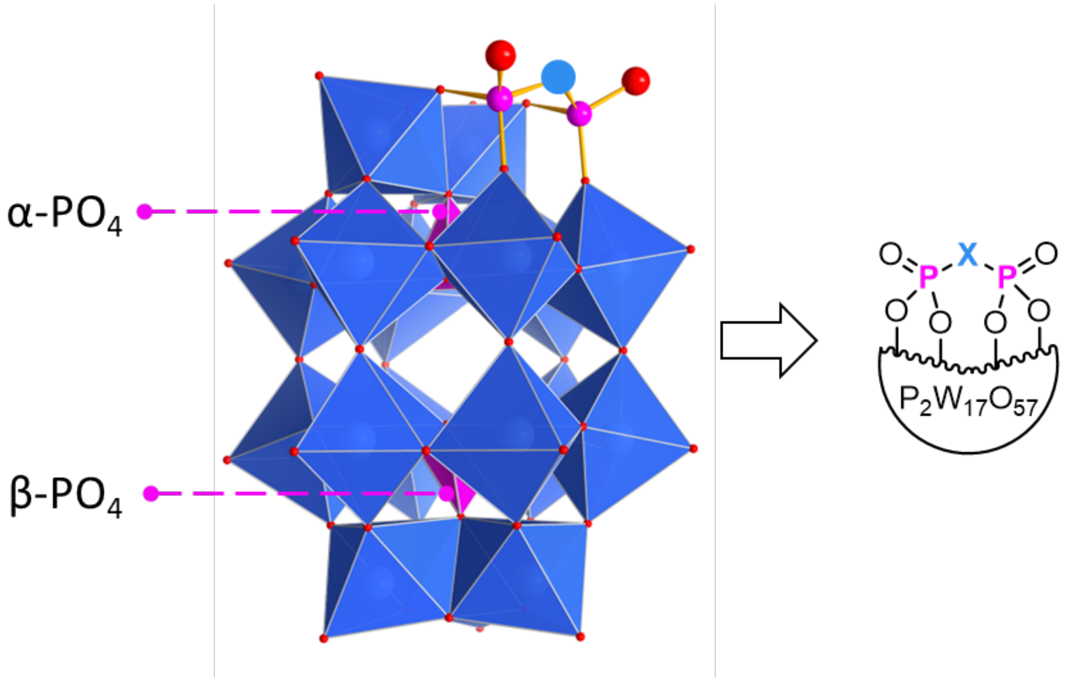

SI Figure 1. Simplification of the generalized diphosphoryl-hybridized Wells–Dawson phosphotungstate structure.

$K_6[P_2W_{17}O_{57}(P_2O_7)]$ (2)

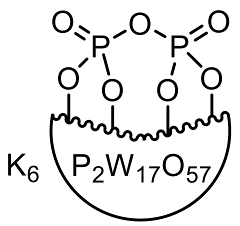

A mixture of tetrasodium pyrophosphate (15 mg, 0.056 mmol, 1.02 eq.) in DMF (20 mL) and 12 M HCl (25 µL, 0.3 mmol, 5.4 eq.) was stirred at 35 °C for 2 min to encourage dissolution. This mixture was then added in one portion to a stirring suspension of $K_{10}[P_2W_{17}O_{61}]$ (250 mg, 0.055 mmol, 1.0 eq.) in DMF (20 mL) and 12 M HCl (25 µL, 0.3 mmol, 5.4 eq.) at 35 °C. Stirring continued at 35 °C for 2 min before being heated at 75 °C for 16 h. The reaction mixture was then cooled to room temperature and centrifuged (8000 rpm, 5 min) to remove the remaining solid. The separated solution was diluted with Et_2O (150 mL) resulting in a white suspension which was centrifuged (8000 rpm, 5 min) and the solvent decanted off. The remaining white solid was then dried *in vacuo*, before being redissolved in a minimum MeCN (5 mL) followed by centrifugation (8000 rpm, 5 min) and decantation to remove any remaining fine precipitate. Trituration with Et_2O (40 mL) resulted in a white suspension which was subjected to centrifugation, decantation, and vacuum drying of the resulting white powder before dissolution in minimum acetone (2 mL). The suspension was centrifuged (8000 rpm, 5 min) and solution separated from any fine precipitate before a final trituration with Et_2O (40 mL) followed by centrifugation, decantation, and vacuum drying of the resulting solid gave the target POM $K_6[P_2W_{17}O_{57}(P_2O_7)]$ (**2**) as a grey solid (49.5 mg, 0.011 mmol, 20%).

^{31}P NMR (162 MHz, CD_3CN) δ −10.98 (α-<u>P</u>O_4), −12.48 (β-<u>P</u>O_4), −18.08 (<u>P</u>O<u>P</u>).

^{31}P NMR (202 MHz, D_2O) δ −10.88 (α-<u>P</u>O_4), −12.55 (β-<u>P</u>O_4), −17.27 (<u>P</u>O<u>P</u>).

ATR-IR (neat, cm^{-1}): 3650, 3201, 1634, 1404, 1180, 1115, 937, 795, 660, 500, 480.

SI Table 1. Mass spectra (ESI) of **2**.

**Assignment**	*z*	***m/z* (calc.)**	***m/z* (obs.)**
$\{H_4[W_{17}P_2O_{57}(P_2O_7)]\}$	2−	2138.35	2138.46
$\{H_4[W_{17}P_2O_{57}(P_2O_7)(H_2O)]\}$	2−	2147.35	2147.44
$\{H_3Na[W_{17}P_2O_{57}(P_2O_7)]\}$	2−	2149.34	2149.45
$\{H_3K[W_{17}P_2O_{57}(P_2O_7)]\}$	2−	2157.33	2157.46
$\{H_3(C_2H_8N)[W_{17}P_2O_{57}(P_2O_7)]\}$	2−	2160.88	2160.93
$\{H_3(C_2H_8N)[W_{17}P_2O_{57}(P_2O_7)](H_2O)\}$	2−	2169.88	2169.92

$K_6[P_2W_{17}O_{57}(P_2O_6NH)]$ (3)

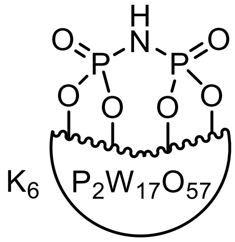

A mixture of tetrasodium imidodiphosphate (15 mg, 0.056 mmol, 1.02 eq.) in DMF (20 mL) and 12 M HCl (25 µL, 0.3 mmol, 5.4 eq.) was stirred at 35 °C for 2 min. This mixture was then added in one portion to a stirring suspension of $K_{10}[P_2W_{17}O_{61}]$ (250 mg, 0.055 mmol) in DMF (20 mL) and 12 M HCl (25 µL, 0.3 mmol, 5.4 eq.) at 35 °C. Stirring continued at 35 °C for 2 min before being heated at 75 °C for 16 h. The reaction mixture was then cooled to room temperature and centrifuged (8000 rpm, 5 min) to remove the remaining solid. The separated solution was diluted with Et_2O (150 mL) resulting in a white suspension which was centrifuged (8000 rpm, 5 min) and the solvent decanted off. The remaining white solid was then dried *in vacuo*, before being redissolved in minimum MeCN (5 mL) where any precipitate was sonicated followed by centrifugation (8000 rpm, 5 min) and decantation to remove any remaining fine precipitate. Trituration with Et_2O (40 mL) resulted in a white suspension which was subjected to centrifugation, decantation, and vacuum drying of the resulting white powder before dissolution in minimum acetone (2 mL) and MeOH (1 mL). The suspension was centrifuged (8000 rpm, 5 min) and solution separated from any fine precipitate before a final trituration with Et_2O (40 mL) followed by centrifugation, decantation, and vacuum drying of the resulting solid which gave the target POM $K_6[P_2W_{17}O_{57}(P_2O_6NH)]$ (**3**) as a grey solid (37.1 mg, 8.2 µmol, 15%).

Crystals suitable for SC-XRD were prepared by vapour diffusion with acetonitrile and methanol as the antisolvent. Please see appendix 3.2 for details. CCDC identification Number: 2239782.

$^{31}P\{^1H\}$ NMR (162 MHz, CD_3CN) δ −5.43 (PNP), −10.96 (α-PO_4), −12.45 (β-PO_4).

$^{31}P\{^1H\}$ NMR (202 MHz, D_2O) δ −5.17 (PNP), −10.86 (α-PO_4), −12.70 (β-PO_4).

ATR-IR (neat, cm^{-1}): 3650, 3201, 2987, 1731, 1390, 1222, 1070, 945, 905, 800, 555.

SI Table 2. Mass spectra (ESI) of **3**.

Assignment	z	*m/z* (calc.)	*m/z* (obs.)
$\{H_4[W_{17}P_2O_{57}(P_2O_6NH)]\}$	2−	2137.86	2137.96
$\{H_3Na[W_{17}P_2O_{57}(P_2O_6NH)]\}$	2−	2148.85	2148.85
$\{H_2Na_2[W_{17}P_2O_{57}(P_2O_6NH)]\}$	2−	2159.84	2159.95
$\{HNa_3[W_{17}P_2O_{57}(P_2O_6NH)]\}$	2−	2170.83	2170.96

$K_6[P_2W_{17}O_{57}(P_2O_6CH_2)]$ (4)

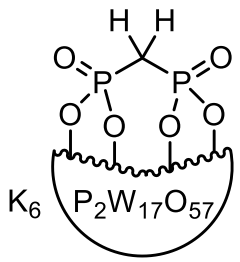

Method A: To a round bottom flask fitted with a magnetic stirrer bar methylenediphosphonic acid (15 mg, 0.085 mmol, 1.6 eq.) was added to DMF (40 mL) followed by $K_{10}[P_2W_{17}O_{61}]$ (250 mg, 0.055 mmol, 1.0 eq.). 12 M HCl (50 µL, 0.6 mmol, 10.9 eq.) was added dropwise to the vigorous stirring mixture before being heated at 70 °C for 16 h. The reaction mixture was then cooled to room temperature and centrifuged (8000 rpm, 5 min) to remove the remaining solid. The separated solution was diluted with Et_2O (150 mL) resulting in a white suspension which was centrifuged (8000 rpm, 5 min) and the solvent decanted off. The remaining white solid was then dried *in vacuo*, before being redissolved in a minimum MeCN (5 mL) followed by centrifugation (8000 rpm, 5 min) and decantation to remove any remaining fine precipitate. Trituration with Et_2O (40 mL) resulted in a white suspension which was subjected to centrifugation, decantation, and vacuum drying of the resulting white powder before dissolution in minimum acetone (2 mL). The suspension was centrifuged (8000 rpm, 5 min) and solution separated from any fine precipitate before a final trituration with Et_2O (40 mL) followed by centrifugation, decantation, and vacuum drying of the resulting solid gave the target POM $K_6[P_2W_{17}O_{57}(P_2O_6CH_2)]$ (**4**) as a grey solid (148 mg, 0.033 mmol, 60%) that takes on a blue hue when exposed to light.

Crystals suitable for SC-XRD were prepared via cation exchange to the tetrabutylammonium salt $(^nBu_4N)_6[P_2W_{17}O_{57}(P_2O_6CH_2)]$ (**4′**) followed by vapour diffusion with DMF and ethyl acetate as the antisolvent. Please see appendix 3.2 for details. CCDC identification Number: 2239783.

Method B[‡]: To a round bottom flask fitted with a magnetic stirrer bar, $K_{10}[P_2W_{17}O_{61}]$ (250 mg, 0.055 mmol, 1.0 eq.), methylenediphosphonic acid (15 mg, 0.085 mmol, 1.6 eq.), and acetonitrile (40 mL) were added. 12 M HCl (30 µL, 0.36 mmol, 6.5 eq.) was added dropwise to the vigorous stirring mixture resulting in the partial solution of the white suspension. The reaction mixture was heated at 70 °C (internal temperature of 61 – 62 °C) for 16 h before being cooled to room temperature. The resulting mixture was centrifuged (8000 rpm, 5 min) to remove the remaining white solid and the separated solution was concentrated under reduced pressure to give a glassy solid. This solid was dissolved in minimum acetone (~2 mL) and centrifuged (6000 rpm, 2 min) to remove any remaining fine precipitate. Trituration with Et_2O (35 mL) gave a white slurry which was subjected to centrifugation, decantation, and vacuum drying of the resulting solid gave the target POM $K_6[P_2W_{17}O_{57}(P_2O_6CH_2)]$ (**4**) as a white solid (197 mg, 0.044 mmol, 85%) that takes on a blue hue when exposed to light.

^{1}H NMR (400 MHz, CD_3CN) δ 2.85-2.61 (m, 2H, $P_2C\underline{H}_2$).

^{1}H NMR (500 MHz, D_2O) δ 2.70 (1H, dt, $^2J_{HH} = -15.1$ Hz, $^2J_{HP} = 20.6$ Hz, $C\underline{H}_2$), 2.58 (1H, dt, $^2J_{HH} = -15.1$ Hz, $^2J_{HP} = 20.6$ Hz, $C\underline{H}_2$)

[‡]Method B can be conducted on the gram scale using $K_{10}[P_2W_{17}O_{61}]$ (1.0 g, 0.22 mmol), methylenediphosphonic acid (60 mg, 0.34 mmol), 12 M HCl (120 µL, 1.44 mmol), and acetonitrile (80 mL) to give target POM **4** (834 mg, 0.185 mmol, 84%) in excellent yield.

$^{1}H\{^{31}P\}$ NMR (500 MHz, D_2O) δ 2.70 (1H, d, $^{2}J_{HH} = -15.1$ Hz, C<u>H</u>$_2$), 2.58 (1H, d, $^{2}J_{HH} = -15.1$ Hz, C<u>H</u>$_2$).

^{13}C NMR (126 MHz, CD_3CN) δ 28.0 (t, $^{1}J_{CP} = 145.0$ Hz, <u>C</u>H$_2$).

$^{31}P\{^{1}H\}$ NMR (202 MHz, CD_3CN) δ 14.49 (<u>P</u>C<u>P</u>), −11.00 (α-<u>P</u>O$_4$), −12.34 (β-<u>P</u>O$_4$).

$^{31}P\{^{1}H\}$ NMR (202 MHz, D_2O) δ 13.21 (<u>P</u>C<u>P</u>), −10.91 (α-<u>P</u>O$_4$), −12.74 (β-<u>P</u>O$_4$).

^{31}P NMR (202 MHz, D_2O) δ 13.23 (t, $^{2}J_{PH} = 20.6$ Hz, <u>P</u>C<u>P</u>), −10.91 (α-<u>P</u>O$_4$), −12.74 (β-<u>P</u>O$_4$).

ATR-IR (neat, cm^{-1}) 3650, 3201,1630, 1445, 1070, 945, 905, 800, 600.

SI Table 3. Mass spectra (ESI) of **4** prepared via *Method A*.

Assignment	*z*	***m/z*** **(calc.)**	***m/z*** **(obs.)**
$\{H_3(C_2H_8N)[W_{17}P_2O_{57}(P_2O_6CH_2)]H_2O\}$	2−	2168.89	2168.97
$\{H_3(C_2H_8N)[W_{17}P_2O_{57}(P_2O_6CH_2)](H_2O)_2\}$	2−	2177.90	2177.99
$\{H_2K(C_2H_8N)[W_{17}P_2O_{57}(P_2O_6CH_2)]\}$	2−	2178.86	2178.97
$\{H_3(C_2H_8N)[W_{17}P_2O_{57}(P_2O_6CH_2)](H_2O)_3\}$	2−	2186.90	2186.98
$\{Na_3(C_2H_8N)[W_{17}P_2O_{57}(P_2O_6CH_2)]\}$	2−	2192.86	2192.98
$\{HNa(C_2H_8N)_2[W_{17}P_2O_{57}(P_2O_6CH_2)]\}$	2−	2193.41	2193.47
$\{H_3(C_2H_8N)[W_{17}P_2O_{57}(P_2O_6CH_2)](H_2O)_4\}$	2−	2195.91	2195.98
$\{Na_2(C_2H_8N)_2[W_{17}P_2O_{57}(P_2O_6CH_2)]\}$	2−	2204.40	2204.48

$K_6[P_2W_{17}O_{57}(P_2O_6CF_2]$ (5)

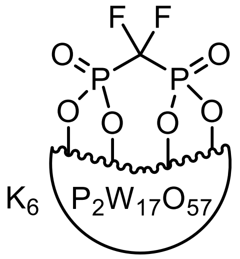

To a round bottom flask fitted with a magnetic stirrer bar $K_{10}[P_2W_{17}O_{61}]$ (250 mg, 0.055 mmol, 1.0 eq.) in acetonitrile (20 mL), a solution of (difluoromethylene)diphosphonic acid (**S2**, 17 mg, 0.081 mmol, 1.55 eq.) in acidified (12 M HCl: 40 µL, 0.48 mmol, 8.7 eq.) acetonitrile (40 mL) was added in one portion to the vigorous stirring mixture resulting in the partial solution of the white suspension. The reaction mixture was heated at 70 °C (internal temperature of 59 – 60 °C) for 16 h before being cooled to room temperature. The resulting mixture was centrifuged (8000 rpm, 5 min) to remove the remaining white solid and the separated solution was concentrated under reduced pressure to give a glassy solid. This solid was dissolved in minimum acetonitrile (~2 mL) and centrifuged (6000 rpm, 2 min) to remove any remaining fine precipitate. Trituration with Et_2O (35 mL) gave a white slurry which was subjected to centrifugation, decantation, and vacuum drying of the resulting solid gave the target POM $K_6[P_2W_{17}O_{57}(P_2O_6CF_2]$ (**5**) as a white solid (221 mg, 0.049 mmol, 89%) that takes on a blue hue with prolonged exposed to light.

^{19}F NMR (377 MHz, CD_3CN) δ −127.32 (t, $^2J_{FP}$ = 91.0 Hz, C<u>F</u>$_2$), −127.34 (t, $^2J_{FP}$ = 89.0 Hz, C<u>F</u>$_2$).

^{19}F NMR (470 MHz, Acetone-d$_6$) δ −127.30 (t, $^2J_{FP}$ = 91.3 Hz, C<u>F</u>$_2$), −127.46 (t, $^2J_{FP}$ = 88.2 Hz, C<u>F</u>$_2$).

^{31}P NMR (162 MHz, CD_3CN) δ −4.30 (t, $^2J_{PF}$ = 91.2 Hz, <u>P</u>C<u>P</u>), −11.00 (α-<u>P</u>O$_4$), −12.31 (β-<u>P</u>O$_4$).

^{31}P NMR (202 MHz, Acetone-d$_6$) δ −3.95 (t, $^2J_{PF}$ = 88.5 Hz, <u>P</u>C<u>P</u>), −10.79 (α-<u>P</u>O$_4$), −12.06 (β-<u>P</u>O$_4$).

ATR-IR (neat, cm^{-1}) 3593, 2980, 1616, 1371, 1084, 1057, 955, 909, 723, 528.

SI Table 4. Mass spectra (ESI) of **5**.

Assignment	*z*	***m/z*** **(calc.)**	***m/z*** **(obs.)**
$\{H_4[W_{17}P_2O_{57}(P_2O_6CF_2)]\}$	2−	2155.35	2155.36
$\{H_4[W_{17}P_2O_{57}(P_2O_6CF_2)]H_2O\}$	2−	2164.35	2164.35
$\{H_3Na[W_{17}P_2O_{57}(P_2O_6CF_2)]\}$	2−	2166.34	2166.35
$\{H_3K[W_{17}P_2O_{57}(P_2O_6CF_2)]H_2O\}$	2−	2183.33	2183.34
$\{H_2Na_2[W_{17}P_2O_{57}(P_2O_6CF_2)]H_2O\}$	2−	2186.34	2186.34
$\{H_2K_2[W_{17}P_2O_{57}(P_2O_6CF_2)]H_2O\}$	2−	2202.31	2202.33
$\{K_4[W_{17}P_2O_{57}(P_2O_6CF_2)]\}$	2−	2231.76	2231.80

^{13}C NMR: Evidence of the carbon and its connectivity in the proposed structure of difluoromethylene diphosphonate hybrid POM **5** is presented below. ^{13}C{^{19}F} NMR (acetone-d$_6$, 126 MHz) spectroscopy (**SI Figure 2**) was run with a 5mm Prodigy BBO probe for enhanced sensitivity, 65000 scans was collected using standard zgpg30 pulse program with a 1 ms adiabatic ^{19}F decoupling pulse to decouple over the bandwidth required. O2P was then set to the ^{19}F resonance (−127.00 ppm) to be decoupled. A broad triplet was identified at 117.1 ppm with an approximate $^1J_{CP}$ coupling constant of 195.0 Hz. The observed shift and coupling constant are within the expected regions for difluoromethylene diphosphonates as seen in literature for alkyl substituted examples.[9] To further validate this data,

^{19}F–^{13}C HSQC NMR (acetone-d_6) spectroscopy was collected with 32 points in the F1, O1P set to ^{19}F resonance (−127.35 ppm), and the $^{1}J_{CF}$ set to the typical one bond ^{13}C-^{19}F coupling of 190 Hz. This data (**SI Figure 3**) supports the C-F connectivity and brings confidence to the suppressed ^{13}C resonance at 117.1 ppm.

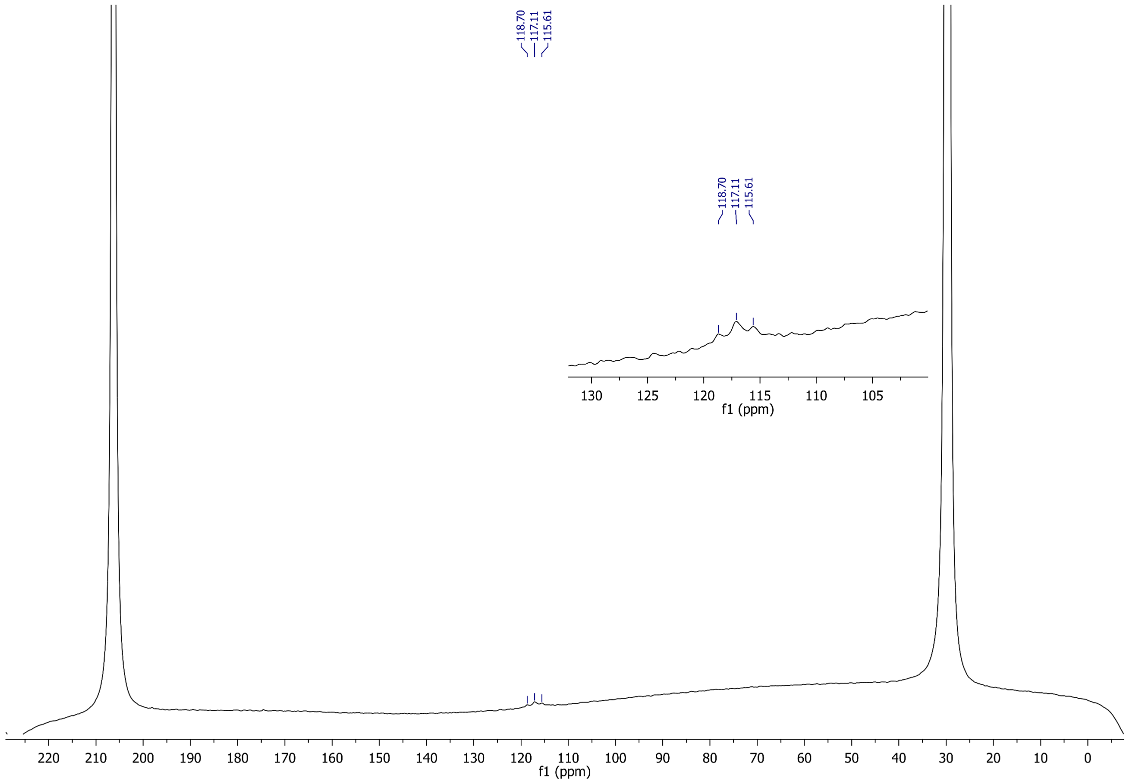

SI Figure 2. $^{13}C\{^{19}F\}$ NMR (126 MHz, Acetone-d_6) of $K_6[P_2W_{17}O_{57}(P_2O_6CF_2]$ (**5**).

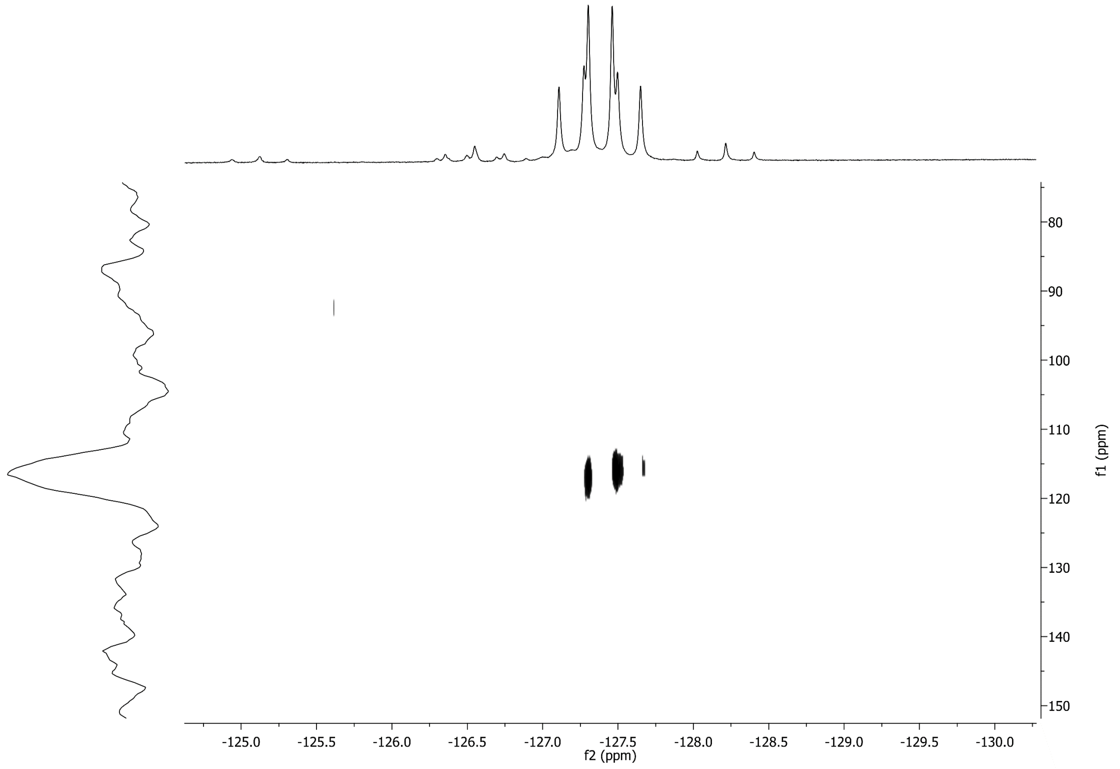

SI Figure 3. ^{19}F–^{13}C HSQC NMR (Acetone-d_6) of $K_6[P_2W_{17}O_{57}(P_2O_6CF_2]$ (**5**).

Propane-2,2-diyldiphosphonic acid (S7)

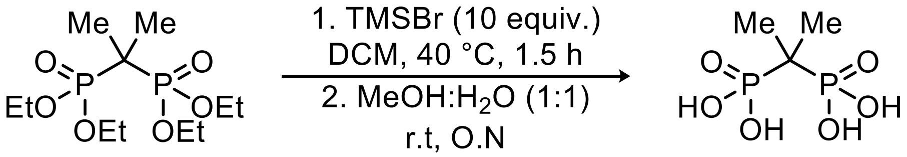

To an oven-dried flask fitted with a magnetic stirrer bar, tetraethyl propane-2,2-diylbis(phosphonate) (**S3**, 197 mg, 0.62 mmol, 1.0 eq.) was added, the flask placed under vacuum and then back filled with argon (3 times). Anhydrous DCM (3.0 mL) was added, followed by the addition of TMSBr (0.82 mL, 949 mg, 6.2 mmol, 10 eq.) in one portion. The resulting orange mixture was then heated to 40 °C and stirring continued for 1.5 h before cooling to room temperature. The solvent was removed under reduced pressure (cold trap) to give an orange residue which was dissolved in MeOH:H_2O (1:1, 20 mL) and the resulting pale yellow solution was stirred at room temperature overnight. The solvent was then removed *in vacuo*, redissolved in water (10 mL), and subjected to lyophilization to give diphosphonic acid **S7** (112 mg, 0.55 mmol, 88%) as a crystalline white solid which was used without further purification.

m.p. 239-241 °C (H_2O).

^{1}H NMR (400 MHz, CD_3OD) δ 1.43 (t, $^3J_{HP}$ = 16.1 Hz, 6H, C(C$\underline{H}_3$)$_2$).

^{13}C NMR (101 MHz, CD_3OD) δ 36.7 (t, $^1J_{CP}$ = 132.0 Hz, $\underline{C}$(CH$_3$)$_2$), 19.8 (t, $^2J_{CP}$ = 4.8 Hz, C($\underline{C}$H$_3$)$_2$).

^{31}P{^{1}H} NMR (162 MHz, CD_3OD) δ 26.76 ($\underline{P}$C$\underline{P}$).

ATR-IR (neat, cm^{-1}) 2662, 2109, 1459, 1178, 1136, 971, 919, 776, 621, 493, 432.

HRMS (ESI). Calc. for $[C_3H_9P_2O_6]^-$ $[M-H]^-$: 212.9874, found: 202.9863. Dimer was also identified, calc. for $[C_6H_{19}P_4O_{12}]^-$ $[M-H]^-$: 406.9827, found: 406.9847.

$K_6[P_2W_{17}O_{57}(P_2O_6C(CH_3)_2)]$ (6)

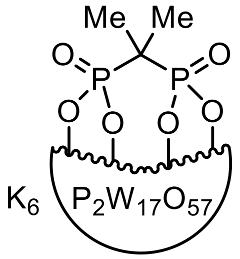

To a round bottom flask fitted with a magnetic stirrer bar $K_{10}[P_2W_{17}O_{61}]$ (250 mg, 0.055 mmol, 1.0 eq.), propane-2,2-diyldiphosphonic acid (**S7**, 17 mg, 0.085 mmol, 1.6 eq.), and acetonitrile (40 mL) were added. 12 M HCl (40 µL, 0.48 mmol, 8.7 eq.) was added dropwise to the vigorous stirring mixture resulting in the partial solution of the white suspension. The reaction mixture was heated at 70 °C (internal temperature of 61 – 62 °C) for 16 h before being cooled to room temperature. The resulting mixture was centrifuged (8000 rpm, 5 min) to remove the remaining white solid and the separated solution was concentrated under reduced pressure to give a glassy solid. This solid was dissolved in minimum acetone (~2 mL) and centrifuged (6000 rpm, 2 min) to remove any remaining fine precipitate. Trituration with Et_2O (35 mL) gave a white slurry which was subjected to centrifugation, decantation, and vacuum drying of the resulting solid gave the target POM $K_6[P_2W_{17}O_{57}(P_2O_6C(CH_3)_2)]$ (**6**) as a white solid (166 mg, 0.037 mmol, 67%) that takes on a blue hue when exposed to light.

^{1}H NMR (400 MHz, CD_3CN) δ 1.55 (t, $^3J_{HP}$ = 17.7 Hz, 3H, C$\underline{H}_3$), 1.49 (t, $^3J_{HP}$ = 17.4 Hz, 3H, C$\underline{H}_3$).

^{13}C NMR (101 MHz, CD_3CN) δ 37.3 (t, $^1J_{CP}$ = 145.8 Hz, $\underline{C}(CH_3)_2$), 18.9 (t, $^2J_{CP}$ = 5.3 Hz, C($\underline{C}H_3)_2$), 18.5 (t, $^2J_{CP}$ = 5.2 Hz, C($\underline{C}H_3)_2$).

^{31}P{^{1}H} NMR (202 MHz, CD_3CN) δ 21.47 ($\underline{P}C\underline{P}$), −11.14 ($\alpha$-$\underline{P}O_4$), −12.33 ($\beta$-$\underline{P}O_4$).

ATR-IR (neat, cm^{-1}) 1691, 1155, 1083, 1055, 956, 907, 718, 525.

SI Table 5. Mass spectra (ESI) of **6**.

Assignment	*z*	*m/z* (calc.)	*m/z* (obs.)
$\{H_4[W_{17}P_2O_{57}(P_2O_6C_3H_6)]H_2O\}$	2−	2160.38	2160.39
$\{H_3Na[W_{17}P_2O_{57}(P_2O_6C_3H_6)]H_2O\}$	2−	2171.37	2171.37
$\{H_2Na_2[W_{17}P_2O_{57}(P_2O_6C_3H_6)]H_2O\}$	2−	2182.36	2182.37
$\{HNa_3[W_{17}P_2O_{57}(P_2O_6C_3H_6)]H_2O\}$	2−	2193.35	2193.36
$\{Na_4[W_{17}P_2O_{57}(P_2O_6C_3H_6)]\}$	2−	2195.34	2195.35
$\{HNa_2K[W_{17}P_2O_{57}(P_2O_6C_3H_6)]H_2O\}$	2−	2201.34	2201.35

$K_6[P_2W_{17}O_{57}(P_2O_6CCH_2)]$ (7)

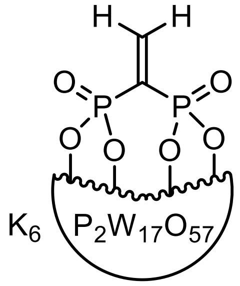

To a round bottom flask fitted with a magnetic stirrer bar $K_{10}[P_2W_{17}O_{61}]$ (250 mg, 0.055 mmol, 1.0 eq.), vinylidene-1,1-diphosphonic acid (**S4**, 16 mg, 0.085 mmol, 1.6 eq.), and acetonitrile (40 mL) were added. 12 M HCl (40 µL, 0.48 mmol, 8.7 eq.) was added dropwise to the vigorous stirring mixture resulting in the partial solution of the white suspension. The reaction mixture was heated at 70 °C (internal temperature of 61 – 62 °C) for 16 h before being cooled to room temperature. The resulting mixture was centrifuged (8000 rpm, 5 min) to remove the remaining white solid and the separated solution was concentrated under reduced pressure to give a glassy solid. This solid was dissolved in minimum acetone (~2 mL) and centrifuged (6000 rpm, 2 min) to remove any remaining fine precipitate. Trituration with Et_2O (35 mL) gave a white slurry which was subjected to centrifugation, decantation, and vacuum drying of the resulting solid gave the target POM $K_6[P_2W_{17}O_{57}(P_2O_6CCH_2)]$ (**7**) as a white solid (181 mg, 0.040 mmol, 73%) that takes on a blue hue when exposed to light.

^{1}H NMR (500 MHz, CD_3CN) δ 6.66 (t, $^3J_{HP}$ = 38.1 Hz, 2H, C=C<u>H</u>$_2$).

^{13}C NMR (126 MHz, CD_3CN) δ 141.5 (C=<u>C</u>H$_2$), 135.3 (t, $^1J_{CP}$ = 183.3 Hz, <u>C</u>=CH$_2$).

^{31}P{^{1}H} NMR (202 MHz, CD_3CN) δ 4.96 (<u>P</u>C<u>P</u>), −11.01 (α-<u>P</u>O$_4$), −12.38 (β-<u>P</u>O$_4$).

ATR-IR (neat, cm^{-1}) 1616, 1084, 1057, 954, 906, 725, 523.

SI Table 6. Mass spectra (ESI) of **7**.

**Assignment**	*z*	***m/z* (calc.)**	***m/z* (obs.)**
$\{H_4[W_{17}P_2O_{57}(P_2O_6C_2H_2)]\}$	2−	2143.36	2143.39
$\{H_4[W_{17}P_2O_{57}(P_2O_6C_2H_2)]H_2O\}$	2−	2152.36	2152.36
$\{H_3Na[W_{17}P_2O_{57}(P_2O_6C_2H_2)]\}$	2−	2154.35	2154.37
$\{H_2Na_2[W_{17}P_2O_{57}(P_2O_6C_2H_2)]H_2O\}$	2−	2174.35	2174.37
$\{HNa_3[W_{17}P_2O_{57}(P_2O_6C_2H_2)]\}$	2−	2176.33	2176.36
$\{H_2NaK[W_{17}P_2O_{57}(P_2O_6C_2H_2)]H_2O\}$	2−	2182.33	2182.37
$\{HNa_2K[W_{17}P_2O_{57}(P_2O_6C_2H_2)]\}$	2−	2184.32	2184.36
$\{HNa_3[W_{17}P_2O_{57}(P_2O_6C_2H_2)]H_2O\}$	2−	2185.34	2185.37
$\{Na_4[W_{17}P_2O_{57}(P_2O_6C_2H_2)]H_2O\}$	2−	2196.33	2196.35

$K_6[P_2W_{17}O_{57}(P_2O_6C(H)CH_3)]$ (8)

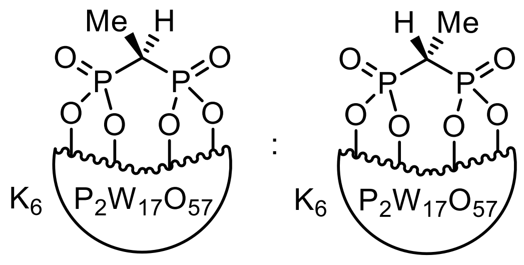

1:1 mixture of inseparable diastereomers

To a round bottom flask fitted with a magnetic stirrer bar $K_{10}[P_2W_{17}O_{61}]$ (250 mg, 0.055 mmol, 1.0 eq.), ethane-1,1-diyldiphosphonic acid (**S5**, 16 mg, 0.084 mmol, 1.5 eq.), and acetonitrile (40 mL) were added. 12 M HCl (40 µL, 0.48 mmol, 8.7 eq.) was added dropwise to the vigorous stirring mixture resulting in the partial solution of the white suspension. The reaction mixture was heated at 70 °C (internal temperature of 61 – 62 °C) for 16 h before being cooled to room temperature. The resulting mixture was centrifuged (8000 rpm, 5 min) to remove the remaining white solid and the separated solution was concentrated under reduced pressure to give a glassy solid. This solid was dissolved in minimum MeCN (~2 mL) and centrifuged (6000 rpm, 2 min) to remove any remaining fine precipitate. Trituration with Et_2O (35 mL) gave a white slurry which was subjected to centrifugation, decantation, and vacuum drying of the resulting solid gave the target POM $K_6[P_2W_{17}O_{57}(P_2O_6C(H)CH_3)]$ (**8**) as a white solid (213 mg, 0.047 mmol, 85%) that takes on a blue hue when exposed to light. POM **8** was isolated as a 1:1 mixture of inseparable diastereomers.

^{1}H NMR (500 MHz, CD_3CN) δ 2.77 – 2.57 (m, 2H, $^{a/b}$C<u>H</u>), 1.56 (dt, $^3J_{HH}$ = 7.0, $^3J_{HP}$ = 18.7 MHz, 3H, ^{a}C<u>H</u>$_3$), 1.49 (dt, $^3J_{HH}$ = 7.1 Hz, $^3J_{HP}$ = 18.4 Hz, 3H, ^{b}C<u>H</u>$_3$).ǂ

^{13}C NMR (126 MHz, CD_3CN) δ 33.2 (t, $^1J_{CP}$ = 145.0 Hz, ^{b}<u>C</u>H), 32.5 (t, $^1J_{CP}$ = 145.2 Hz, ^{a}<u>C</u>H), 10.4 (t, $^2J_{CP}$ = 6.5 Hz, ^{b}<u>C</u>H$_3$), 9.8 (t, $^2J_{CP}$ = 6.3 Hz, ^{a}<u>C</u>H$_3$).ǂ

^{31}P{^{1}H} NMR (202 MHz, CD_3CN) δ 18.49 (<u>P</u>C<u>P</u>), −11.06 (α-<u>P</u>O$_4$), −11.09 (α-<u>P</u>O$_4$), −12.29 (β-<u>P</u>O$_4$), −12.34 (β-<u>P</u>O$_4$).

^{31}P{^{1}H} NMR (202 MHz, D_2O) δ 17.86 (<u>P</u>C<u>P</u>), 17.60 (<u>P</u>C<u>P</u>), −10.95 (α-<u>P</u>O$_4$), −11.02 (α-<u>P</u>O$_4$), −12.74 (β-<u>P</u>O$_4$), −12.78 (β-<u>P</u>O$_4$).

ATR-IR (neat, cm^{-1}) 3468, 1613, 1160, 1083, 955, 907, 702, 522.

SI Table 7. Mass spectra (ESI) of **8**.

**Assignment**	*z*	***m/z*** **(calc.)**	***m/z*** **(obs.)**
$\{Na_3H[W_{17}P_2O_{57}(P_2O_6C_2H_4)]\}$	2−	2177.34	2177.35
$\{Na_3K[W_{17}P_2O_{57}(P_2O_6C_2H_4)]\}$	2−	2196.32	2196.32
$\{NaK_3[W_{17}P_2O_{57}(P_2O_6C_2H_4)]\}$	2−	2212.79	2212.80
$\{K_3H[W_{17}P_2O_{57}(P_2O_6C_2H_4)]H_2O\}$	2−	2210.80	2210.81
$\{K_2NaH[W_{17}P_2O_{57}(P_2O_6C_2H_4)]H_2O\}$	2−	2202.32	2202.32
$\{K_2Na_2[W_{17}P_2O_{57}(P_2O_6C_2H_4)]H_2O\}$	2−	2213.31	2213.31
$\{K_3Na[W_{17}P_2O_{57}(P_2O_6C_2H_4)]H_2O\}$	2−	2221.80	2221.80

ǂResonances have been assigned to one of two diastereomers where possible, labelled as *a* or *b*, based on correlations to each other as indicated by 2D NMR spectroscopy. The specific diastereomer that *a* and *b* represent has not been determined.

Tetraethyl (2-(anthracen-9-yl)ethane-1,1-diyl)bis(phosphonate) (S8)

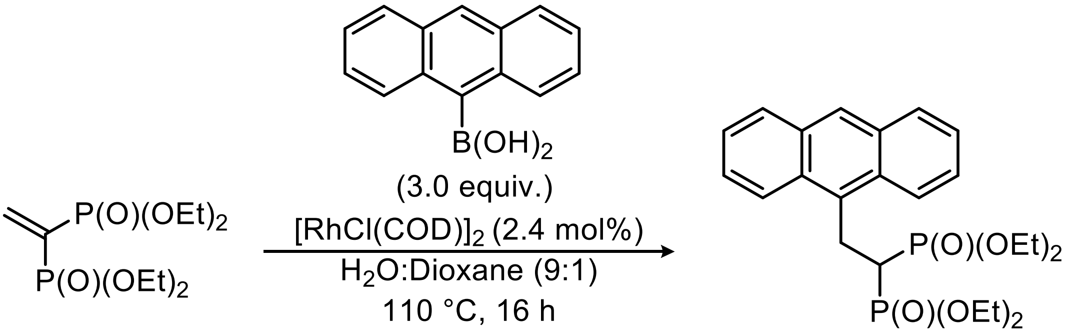

Tetraethyl (2-(anthracen-9-yl)ethane-1,1-diyl)bis(phosphonate) (**S8**) was prepared via a Rh(I) mediated 1,4-conjugate addition of aryl boronic acids to vinylidenebisphosphonate esters as reported by Strukul, G and co-workers.[10]

To an oven-dried microwave vial fitted with a magnetic stirrer bar, vinylidene-1,1-diphosphonic acid (**S4**, 120 mg, 0.4 mmol, 1.0 eq.) and 9-anthraceneboronic acid (266 mg, 1.2 mmol, 3.0 eq.) were added. The vial was capped with a crimp cap seal and placed under vacuum then back filled with argon (3 times). To this, degassed DI water (3.0 mL) and $[RhCl(COD)]_2$/dioxane solution (300 µL, 0.033 M, 0.99 µmol, 2.4 mol% dimer, 4.8 mol% Rh)ǂ were added. The resulting yellow mixture was heated at 110 °C for 16 h before being cooled to room temperature, diluted with water (5 mL), and extracted with ethyl acetate (3 x 5 mL). The combined organic fractions were then washed with brine (5 mL), dried (Na_2SO_4), filtered, and concentrated under reduced pressure. The resulting mixture was subjected to flash chromatography (silica, 80% ethyl acetate:cyclohexane → 100% ethyl acetate) to give diphosphonate **S8** (143 mg, 0.30 mml, 75%) as an orange oil.

$\boldsymbol{R_f}$ = 0.19 in 100% ethyl acetate.

^{1}H NMR (400 MHz, CD_3CN) δ 8.47 (br. s, 2H, ArC<u>H</u>), 8.45 (br. s, 1H, ArC<u>H</u>), 8.05 (d, J = 8.3 Hz, 2H, ArC<u>H</u>), 7.59-7.54 (m, 2H, ArC<u>H</u>), 7.52-7.48 (m, 2H. ArC<u>H</u>), 4.28 (td, $^3J_{HH}$ = 6.8, $^3J_{HP}$ = 15.7 Hz, 2H, C<u>H</u>$_2$Ar), 4.04-3.83 (m, 8H, C<u>H</u>$_2$CH$_3$), 2.87 (tt, $^3J_{HH}$ = 6.8, $^2J_{HP}$ = 23.2 Hz, 1H. P$_2$C<u>H</u>), 1.08 (t, $^3J_{HH}$ = 7.1 Hz, 6H, CH$_2$C<u>H</u>$_3$), 1.00 (t, $^3J_{HH}$ = 7.1 Hz, 6H, CH$_2$C<u>H</u>$_3$).

^{13}C NMR (126 MHz, CD_3CN) δ 132.5 (3 x Ar<u>C</u>), 131.1 (2 x Ar<u>C</u>), 130.0 (2 x Ar<u>C</u>H), 127.6 (Ar<u>C</u>H), 126.7 (2 x Ar<u>C</u>H), 126.00 (2 x Ar<u>C</u>H), 125.97 (2 x Ar<u>C</u>H), 63.4 (d, $^2J_{CP}$ = 6.6 Hz, <u>C</u>H$_2$CH$_3$), 63.0 (d, $^2J_{CP}$ = 6.5 Hz, <u>C</u>H$_2$CH$_3$), 38.7 (t, $^1J_{CP}$ = 130.8 Hz, P$_2$<u>C</u>H), 24.5 (t, $^2J_{CP}$ = 4.4, <u>C</u>H$_2$Ar), 16.4 (d, $^3J_{CP}$ = 6.6 Hz, <u>C</u>H$_3$), 16.3 (d, $^3J_{CP}$ = 6.6 Hz, <u>C</u>H$_3$).

^{31}P{^{1}H} NMR (162 MHz, CD_3CN) δ 22.3 (<u>P</u>C<u>P</u>).

ATR-IR (neat, cm^{-1}) 2980, 2906, 1669, 1600, 1391, 1243, 1161, 1016, 964, 733, 601, 521.

HRMS (ESI) Calc. for $[C_{24}H_{33}O_6P_2]^+$ $[M+H]^+$: 479.1752, found: 479.1745. Calc. for $[C_{24}H_{32}O_6P_2Na]^+$ $[M+Na]^+$: 501.1572, found: 501.1564.

ǂA stock solution of $[RhCl(COD)]_2$ in 1,4-dioxane was prepared by dissolving $[RhCl(COD)]_2$ (48 mg, 0.097 mmol) in 3 mL of 1,4-dioxane that had been degassed by the freeze pump thaw method.

(2-(Anthracen-9-yl)ethane-1,1-diyl)diphosphonic acid (S9)

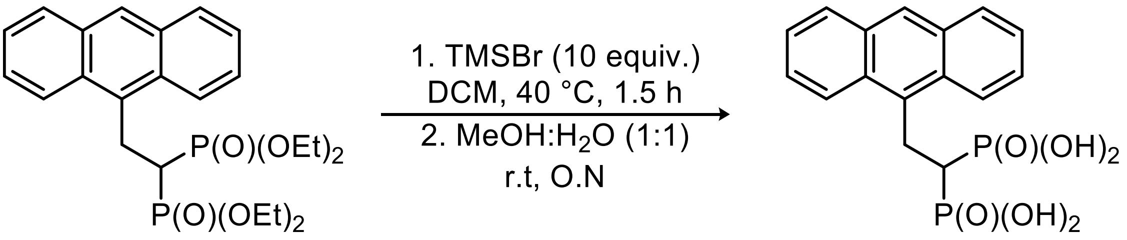

To an oven-dried flask fitted with a magnetic stirrer bar, diphosphonate **S8** (71.5 mg, 0.15 mmol, 1.0 eq.) was added, the flask placed under vacuum and then back filled with argon (3 times). Anhydrous DCM (1.5 mL) was added followed by the addition of TMSBr (198 µL, 229 mg, 1.5 mmol, 10 eq.) in one portion. The resulting orange mixture was then heated to 40 °C and stirring continued for 1.5 h before cooling to room temperature. The solvent was removed under reduced pressure (cold trap) to give a orange residue which was dissolved in MeOH:H_2O (1:1, 20 mL) and the resulting yellow solution was stirred at room temperature overnight. The solvent was then removed *in vacuo*, redissolved in water (10 mL), and concentrated under reduced pressure to give diphosphonic acid **S9** (51.2 mg, 0.14 mmol, 93%) as a crystalline yellow solid which was used without further purification.

m.p. Decomposition occurred in the region of 255–257 °C.

^{1}H NMR (400 MHz, CD_3OD) δ 8.55 (d, J = 9.0 Hz, 2H, ArC<u>H</u>), 8.39 (s, 1H, ArC<u>H</u>), 8.01 (d, J = 8.4 Hz, 2H, ArC<u>H</u>), 7.52 (app. t, 2H, ArC<u>H</u>), 7.44 (app. t, 2H, ArC<u>H</u>), 4.31 (td, $^3J_{HH}$ = 6.7, $^3J_{HP}$ =15.8 Hz, 2H, C<u>H</u>$_2$Ar), 2.90 (tt, $^3J_{HH}$ = 6.6, $^2J_{HP}$ = 23.0 Hz, 1H, P_2C<u>H</u>).

^{13}C NMR (101 MHz, CD_3OD) δ 133.1 (3 x Ar<u>C</u>), 131.6 (2 x Ar<u>C</u>), 130.2 (2 x Ar<u>C</u>H), 127.8 (Ar<u>C</u>H), 126.6 (2 x Ar<u>C</u>H), 126.2 (2 x Ar<u>C</u>H), 125.8 (2 x Ar<u>C</u>H), 41.0 (t, $^1J_{CP}$ = 126.1 Hz, P_2<u>C</u>H), 24.6 (t, $^2J_{CP}$ = 4.1 Hz, <u>C</u>H$_2$Ar).

^{31}P{^{1}H} NMR (162 MHz, CD_3OD) δ 21.45 (<u>P</u>C<u>P</u>).

ATR-IR (neat, cm^{-1}) 2980, 2888, 2324, 2122, 1141, 1022, 963, 921, 890, 726, 412.

HRMS (ESI) Calc. for $[C_{16}H_{15}O_6P_2]^-$ $[M-H]^-$: 365.0344, found: 365.0346.

$K_6[P_2W_{17}O_{57}(P_2O_6C(H)CH_29$-anthryl)] (9)

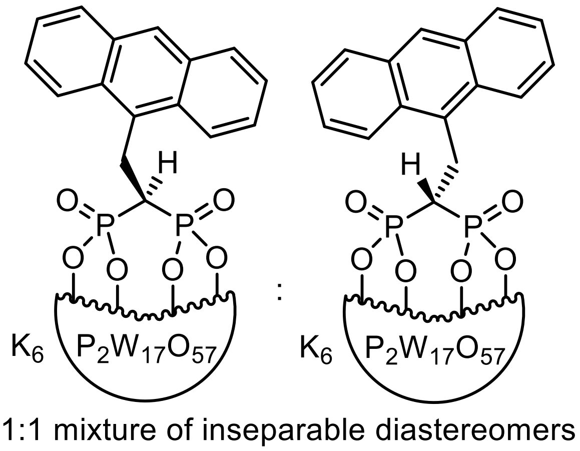

To a round bottom flask fitted with a magnetic stirrer bar $K_{10}[P_2W_{17}O_{61}]$ (250 mg, 0.055 mmol, 1.0 eq.), (2-(anthracen-9-yl)ethane-1,1-diyl)diphosphonic acid (**S9**, 29.7 mg, 0.081 mmol, 1.5 eq.), and acetonitrile (40 mL) were added. 12 M HCl (40 µL, 0.48 mmol, 8.7 eq.) was added dropwise to the vigorous stirring mixture resulting in the partial solution of the white suspension. The reaction mixture was heated at 70 °C (internal temperature of 60 – 61 °C) for 16 h before being cooled to room temperature. The resulting orange mixture was centrifuged (8000 rpm, 5 min) to remove the remaining white solid and the separated solution was concentrated under reduced pressure to give a glassy dark green solid. This solid was dissolved in minimum MeCN (~2 mL) and centrifuged (6000 rpm, 2 min) to remove any remaining fine precipitate. Trituration with Et_2O (35 mL) gave a purple slurry which was subjected to centrifugation, decantation, and vacuum drying of the resulting solid gave the target POM $K_6[P_2W_{17}O_{57}(P_2O_6C(H)CH_29$-anthryl)] (**9**) as a dark green solid (232 mg, 0.050 mmol, 91%). POM **9** was isolated as a 1:1 mixture of inseparable diastereomers.

^{1}H NMR (500 MHz, D_2O) δ 8.71-8.54 (m, 6H, ArC<u>H</u>), 8.13 (app. t, 4H, ArC<u>H</u>), 7.73-7.64 (m, 3H, ArC<u>H</u>), 7.59-7.54 (m, 5H, ArC<u>H</u>), 4.57-4.37 (m, 4H, C<u>H</u>$_2$Ar), 3.28 (tt, $^3J_{HH}$ = 7.6 Hz, $^2J_{HP}$ = 22.1 Hz, P_2C<u>H</u>), 3.07 (tt, $^3J_{HH}$ = 6.0 Hz, $^2J_{HH}$ = 21.6 Hz, 1H, P_2C<u>H</u>).

^{13}C NMR (126 MHz, D_2O) δ 131.24 (3 x Ar<u>C</u>), 131.18 (3 x Ar<u>C</u>), 129.9 (2 x Ar<u>C</u>H), 129.8 (2 x Ar<u>C</u>H), 129.0 (2 x Ar<u>C</u>H), 128.9 (2 x Ar<u>C</u>H), 126.6 (Ar<u>C</u>H), 126.5 (Ar<u>C</u>H), 126.0 (2 x Ar<u>C</u>H), 125.8 (2 x Ar<u>C</u>H), 125.5 (2 x Ar<u>C</u>H), 125.2 (2 x Ar<u>C</u>H), 125.2 (2 x Ar<u>C</u>H), 125.0 (2 x Ar<u>C</u>H), 40.8 (t, $^1J_{CP}$ = 136.3 Hz, P_2<u>C</u>H), 40.5 (t, $^1J_{CP}$ = 138.0 Hz, P_2<u>C</u>H), 24.1 (<u>C</u>H$_2$Ar), 23.1 (<u>C</u>H$_2$Ar).

^{31}P{^{1}H} NMR (202 MHz, D_2O) δ 15.09 (<u>P</u>C<u>P</u>), 14.70 (<u>P</u>C<u>P</u>), −10.92 (α-<u>P</u>O$_4$), −11.02 (α-<u>P</u>O$_4$), −12.74 (β-<u>P</u>O$_4$), −12.84 (β-<u>P</u>O$_4$).

ATR-IR (neat, cm^{-1}) 3476, 2980, 1611, 1154, 1083, 954, 907, 710, 524.

SI Table 8. Mass spectra (ESI) of **9**.

Assignment	*z*	*m/z* (calc.)	*m/z* (obs.)
$\{H_4[W_{17}P_2O_{57}(P_2O_6C_{16}H_{12})]H_2O\}$	2−	2241.40	2241.41
$\{K_3H[W_{17}P_2O_{57}(P_2O_6C_{16}H_{12})]H_2O\}$	2−	2260.38	2260.39
$\{Na_3H[W_{17}P_2O_{57}(P_2O_6C_{16}H_{12})]\}$	2−	2265.37	2265.38
$\{KNaH_2[W_{17}P_2O_{57}(P_2O_6C_{16}H_{12})]H_2O\}$	2−	2271.37	2271.38
$\{K_2H_2[W_{17}P_2O_{57}(P_2O_6C_{16}H_{12})]H_2O\}$	2−	2279.86	2279.87
$\{KNa_2H[W_{17}P_2O_{57}(P_2O_6C_{16}H_{12})]H_2O\}$	2−	2282.36	2282.37
$\{K_2NaH[W_{17}P_2O_{57}(P_2O_6C_{16}H_{12})]H_2O\}$	2−	2290.85	2290.86

2.2 Hydrolytic stability experiments of S6, 2, 3, and 4

Method: A sample of polyoxometalate (approx. 30 mg) was dissolved in D_2O (1.5 mL) and NMR spectra taken periodically over the course of weeks. These solutions were kept in their NMR tubes and stored in the dark under ambient conditions between measurements. No special precautions were taken to avoid exposure to the atmosphere.

Previous hydrolysis studies of phenylphosphonate-hybridized Keggin POMs of general formula $[PhP(O)]_2X^{n+}W_{11}O_{39}^{(8-n)-}$ ($X = P^{5+}$, Si^{4+}) have shown these clusters to be relatively unstable.[11] For the P Keggin POM (**SI Scheme 1, a**), partial cleavage of the phosphoryl group occurred when dissolved in wet DMSO, and the intermediate cluster was isolable. The Si-centred Keggin POM (**SI Scheme 1, b**) was shown to slowly undergo complete hydrolytic cleavage of the phosphoryl group from the POM cluster under similar conditions to give $PhPO_3^{2-}$ and the lacunary precursor complex $SiW_{11}O_{39}^{8-}$, no intermediate partially hydrolysed species were observed.

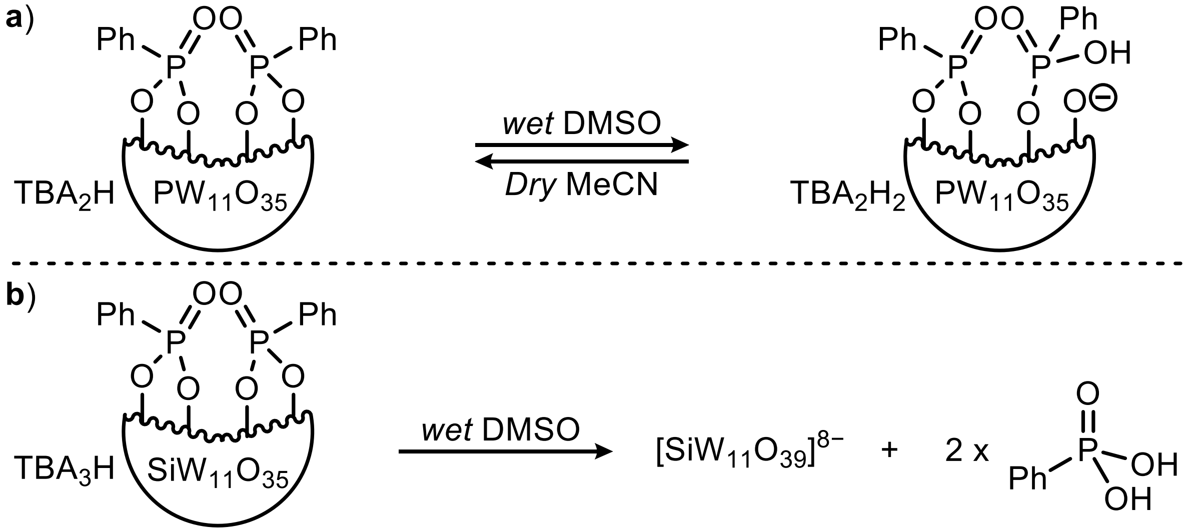

SI Scheme 1. Hydrolysis pathways of phenylphosphonate-hybridized Keggin POMs.[11]

Similar studies were conducted with phosphoryl hybridized Wells-Dawson POMs in 10% (v/v) D_2O/DMSO-d_6 solution.[12] Over 24 h, aryl phosphonate hybrid POMs showed no appreciable decomposition, while non-arylated phosphonate derivatives were susceptible to hydrolytic cleavage to give a mixture of species including loss of one or both phosphoryl groups.

Here, we found the aryl phosphonate hybrid $K_6[P_2W_{17}O_{57}(P_2O_6Ph_2)]$ (**S6**) to show similar stability over 24 h when dissolved in D_2O (**SI Figure 4**). However, monitoring of the reaction mixture over a longer timescale showed the cluster to slowly undergo complete hydrolysis as observed by the gradual formation of $PhPO_3H_2$ (14 ppm). Minor peaks were observed for the concurrent formation of $[P_2W_{17}O_{61}]^{10-}$, however, further decomposition of this metastable cluster gave the plenary Wells-Dawson POM $[P_2W_{18}O_{62}]^{6-}$ (**SI Scheme 2, a**), which were identified as a new species after 4 days with significant accumulation after 12 days.

The diphosphoryl hybrid POMs (**2** and **3**) similarly underwent direct cleavage to their components without observation of the partially hydrolysed species (**SI Scheme 2, b**). Pyrophosphate hybrid POM **2** had a similar lifetime to **S6**, with a notable increase in $[P_2W_{18}O_{62}]^{6-}$ formation after 4 days and trace phosphoric acid species apparent (**SI Figure 5**). Imidophosphate hybrid POM **3** was the least stable under aqueous conditions, rapidly forming phosphate and phosphoramidate by-products with significant amounts of $[P_2W_{18}O_{62}]^{6-}$ present (**SI Figure 6**). Conversely, methylene diphosphonate hybrid POM **4** showed excellent stability, with no notable change for the duration of this study

(28 days). In all examples (**S6**, **2-4**), a secondary POM isomer was observed (indicated by *) when solubilised in D_2O.

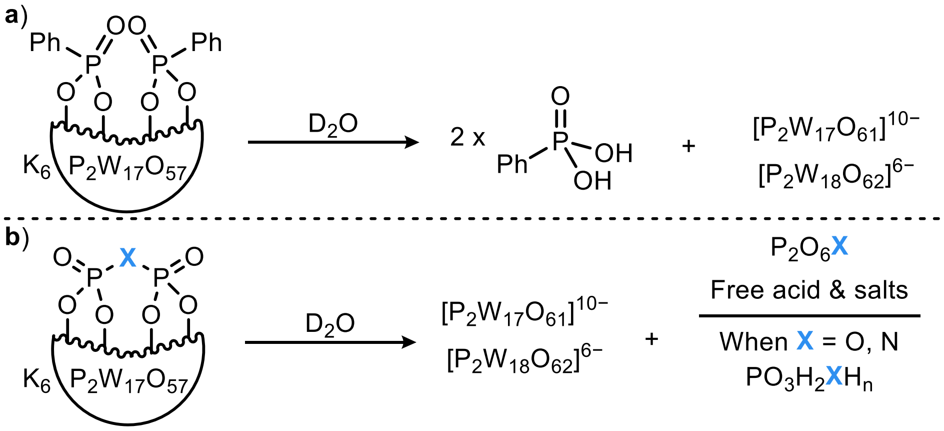

SI Scheme 2. Proposed hydrolysis pathways of phosphonate hybrid POM **S6** and diphosphonate hybrid POMs **2-4**.

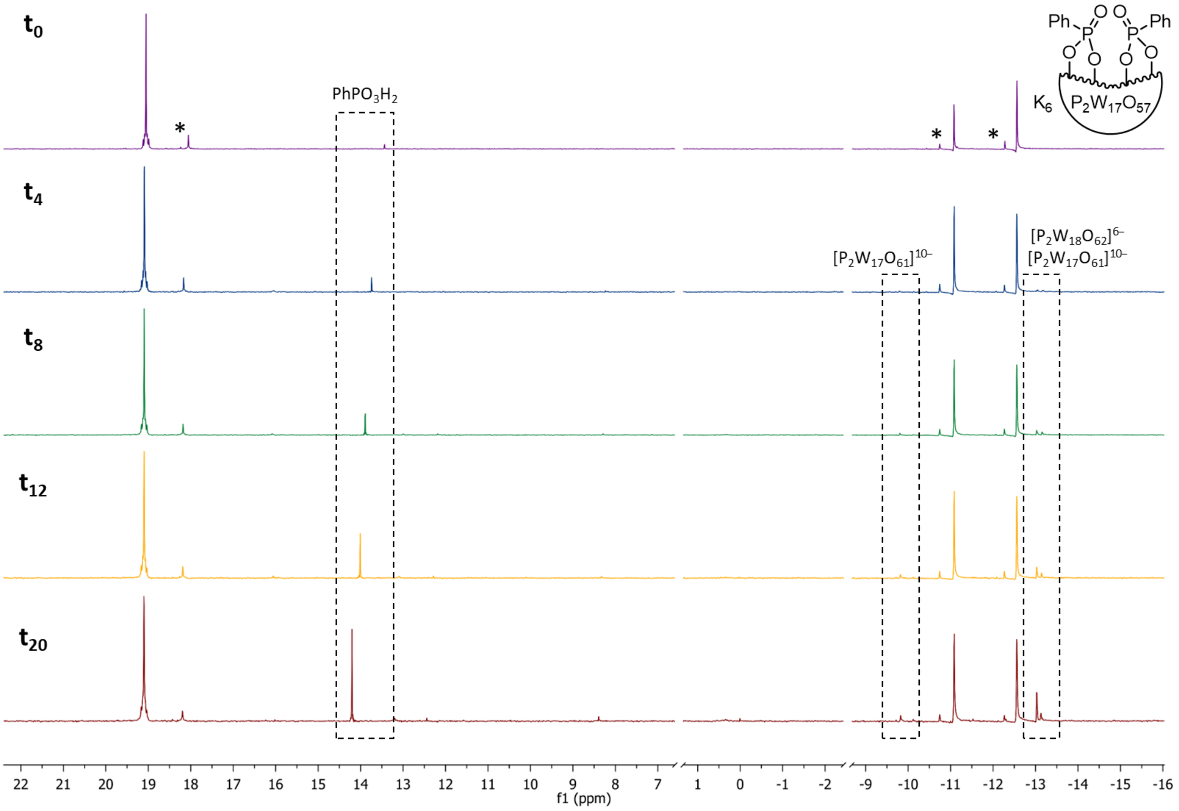

SI Figure 4. Solution of $K_6[P_2W_{17}O_{57}(P_2O_6Ph_2)]$ (**S6**) in D_2O monitored by $^{31}P\{^1H\}$ NMR (202 MHz) over t_{days}. Spectra window restrained to highlight areas with observed resonances. * Indicates POM isomer.

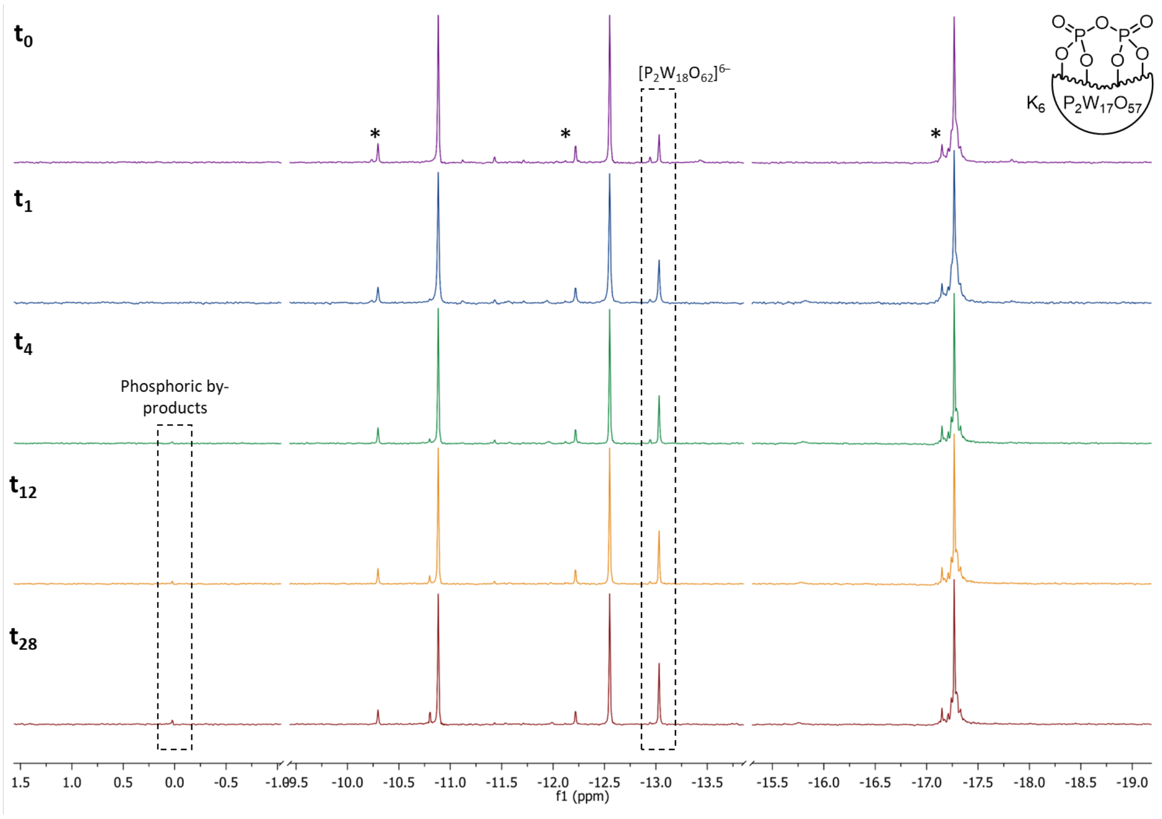

SI Figure 5. Solution of $K_6[P_2W_{17}O_{57}(P_2O_7)]$ (**2**) in D_2O monitored by $^{31}P\{^1H\}$ NMR (202 MHz) over t_{days}. Spectra window restrained to highlight areas with observed resonances. * Indicates POM isomer.

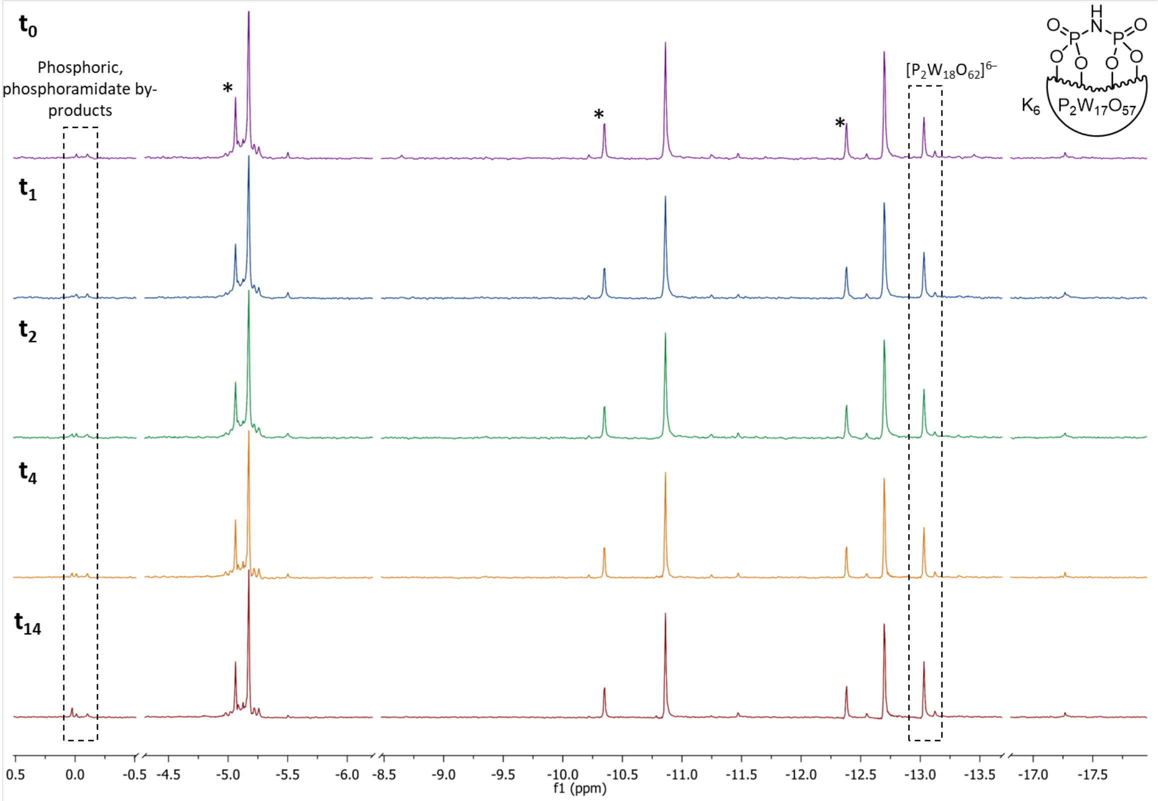

SI Figure 6. Solution of $K_6[P_2W_{17}O_{57}(P_2O_6NH)]$ (**3**) in D_2O monitored by $^{31}P\{^1H\}$ NMR (202 MHz) over t_{days}. Spectra window restrained to highlight areas with observed resonances. * Indicates POM isomer.

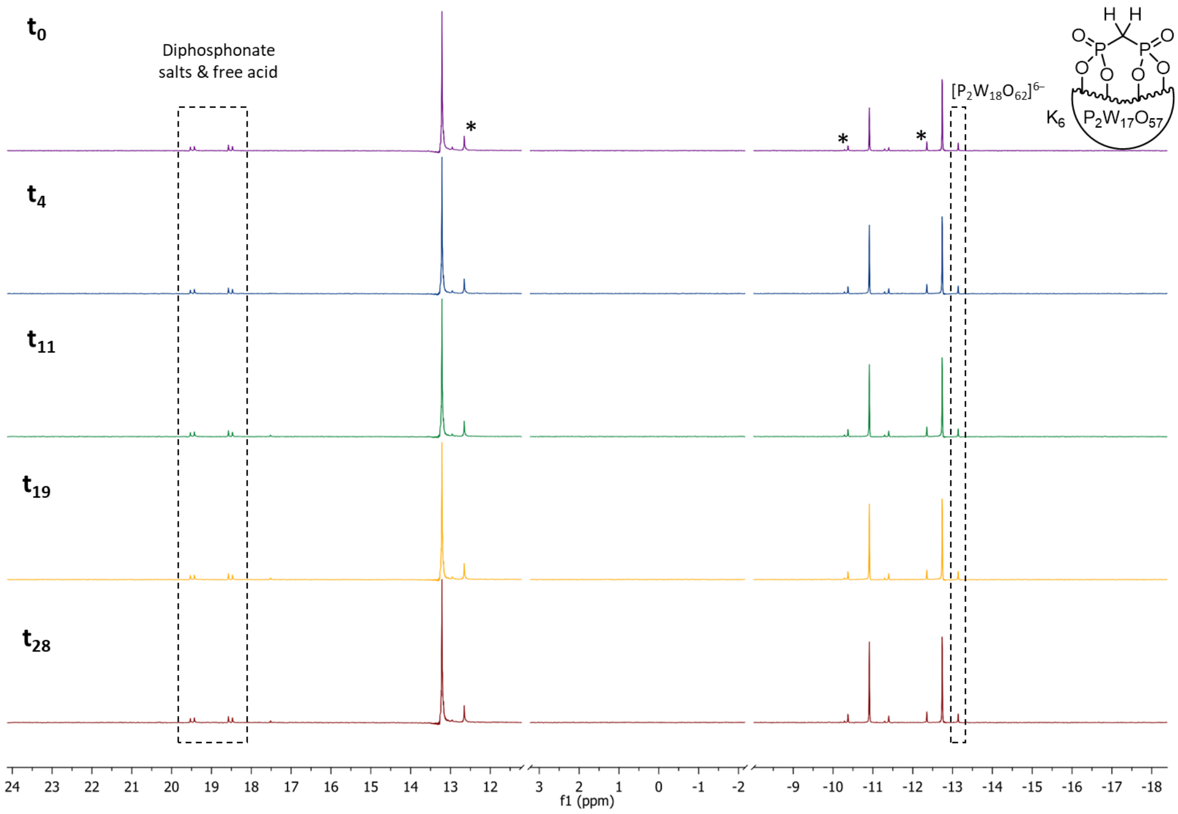

SI Figure 7. Solution of $K_6[P_2W_{17}O_{57}(P_2O_6CH_2)]$ (**4**) in D_2O monitored by $^{31}P\{^1H\}$ NMR (202 MHz) over t_{days}. Spectra window restrained to highlight areas with observed resonances. * Indicates POM isomer.

2.3 Cyclic voltammetry studies

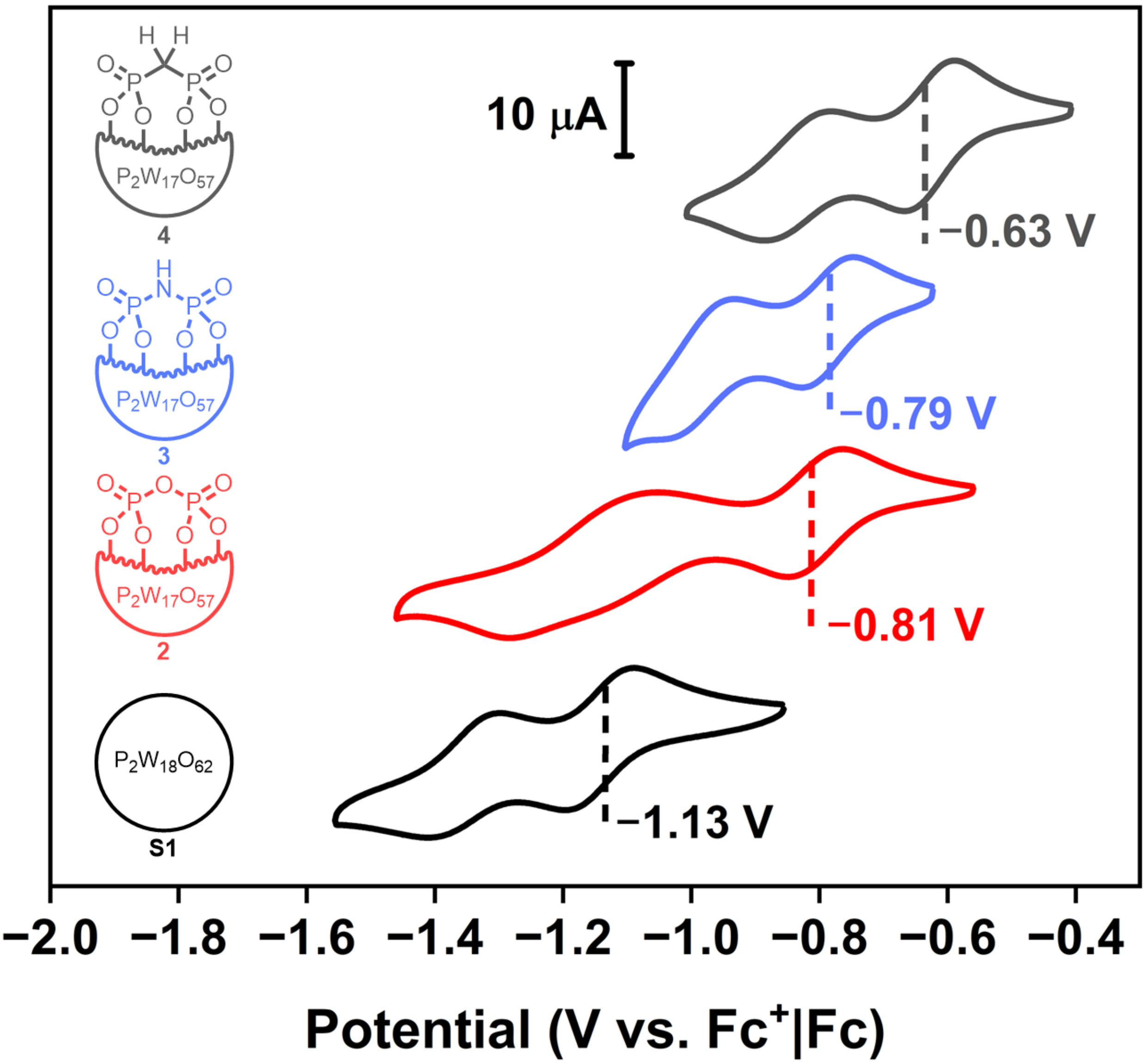

SI Figure 8. Cyclic voltammograms of 1 mM of compounds of **S1** and **2**-**4** in DMF with 0.1 M $^{n}Bu_4NPF_6$ supporting electrolyte at 100 mVs^{-1} vs. Fc^{+}|Fc redox couple. Glassy carbon working electrode (d = 3 mm), Pt wire counter electrode, and a Ag wire pseudo reference were used. The $E_{1/2}$ potentials are highlighted for the first redox process.

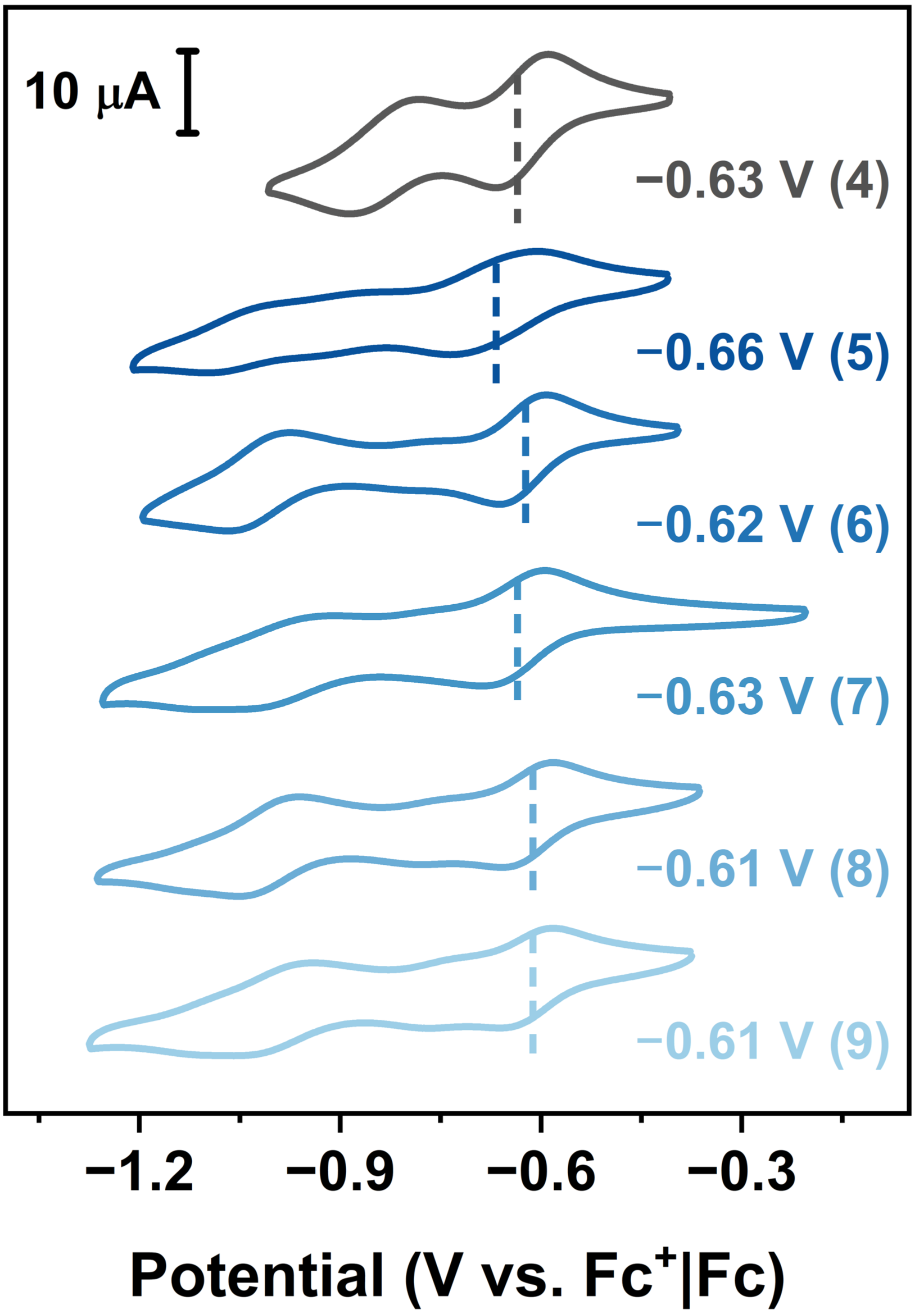

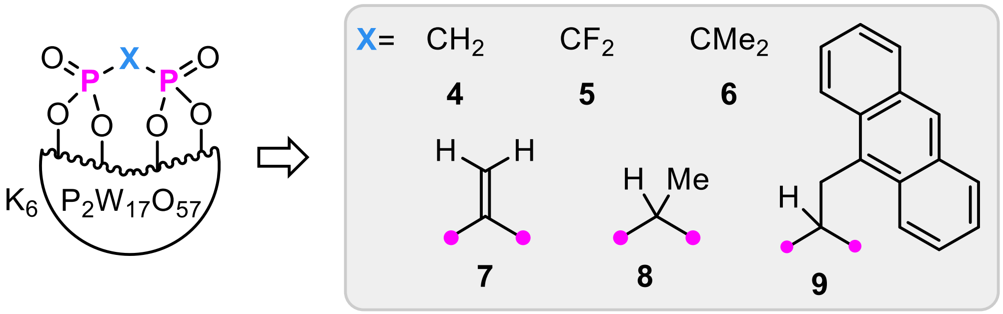

SI Figure 9. Cyclic voltammogram of 1 mM of compounds of **4-9** in DMF with 0.1 M $^{n}Bu_4NPF_6$ supporting electrolyte at 100 mVs^{-1} vs. Fc^+|Fc redox couple. Glassy carbon working electrode (d = 3 mm), Pt wire counter electrode, and a Ag wire pseudo reference were used. The $E_{1/2}$ potentials are highlighted for the first redox process.

2.3 Computational details

Density functional theory (DFT) was used with the BP86 functional and CRENBL basis set and effective core potential (ECP) to identify minimum energy geometries. Solvent was included using the polarisable continuum model (PCM), with the conductor-like screening algorithm (C-PCM). A dielectric constant of 36.7 was used. For tungsten atoms, a van der Waals radius of 2.1 Å was employed. The enthalpy of reduction was calculated as:

$$\Delta H_{reduction} = H_{reduced}^{elec} - H_{non-reduced}^{elec}$$

i.e. the difference in energy between the minimum energy geometries of the reduced and non-reduced POM. Correcting for the SHE and Fc^{+}|Fc absolute values (and using the Gibbs energies) according to reference [**13**] gave directly comparable values denoted as E_{ET}^{calc}. All DFT calculations were performed with the Q-Chem 5.4 package.

Kohn-Sham molecular orbitals were plotted using IQmol, with an isovalue of 0.02 $Å^{-3}$.

The qualitative trend of the reduction enthalpies matches the experimental CV data. It should be noted that the computed values are absolute values, while the CV data are relative to Fc^{+}|Fc.

Table S9. Calculated enthalpies of reduction. [a)] E_{ET}^{calc} values calculated according to the procedure in reference [**13**]. The reference value for the standard hydrogen electrode is taken from [**14**].

Compound	Bridging atom	Enthalpy of reduction (eV)	E_{ET}^{calc} / V[a]
**2**	-O-	-4.526	-0.96
**3**	-NH-	-4.453	-0.81
**4**	$-CH_2-$	-4.422	-0.67

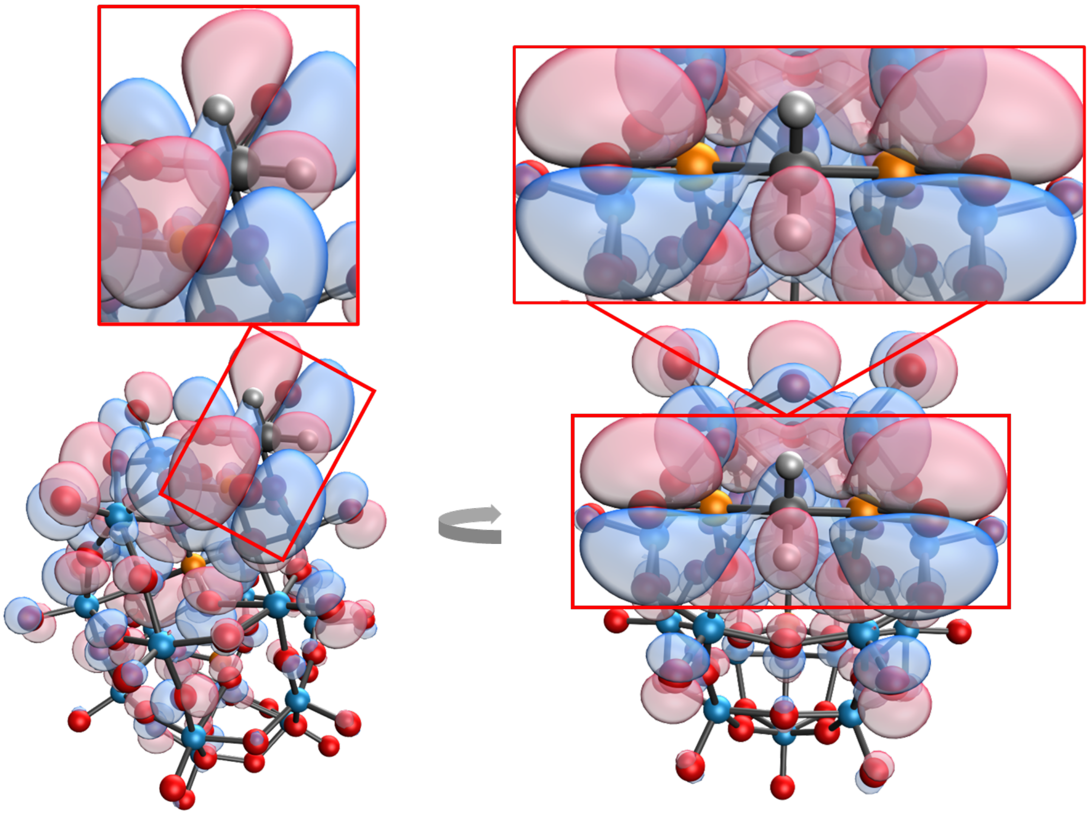

SI Figure 10. HOMO-1 of the methylene bridged POM **4**; (inset) close-up view of the non-symmetrical electron density on the C-H bonds of the methylene group.

3.0 Appendix

3.1 NMR Spectra

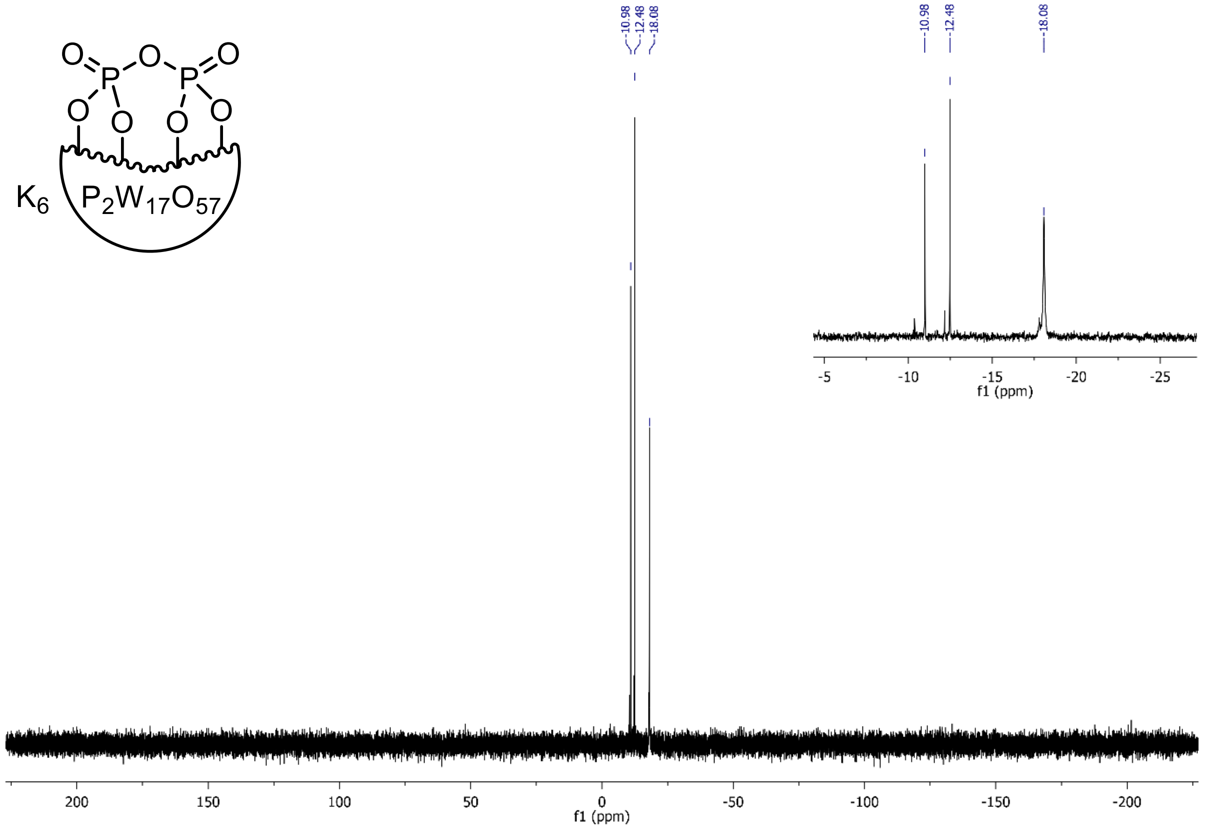

SI Figure 11. ^{31}P NMR (162 MHz, CD_3CN) of $K_6[P_2W_{17}O_{57}(P_2O_7)]$ (**2**).

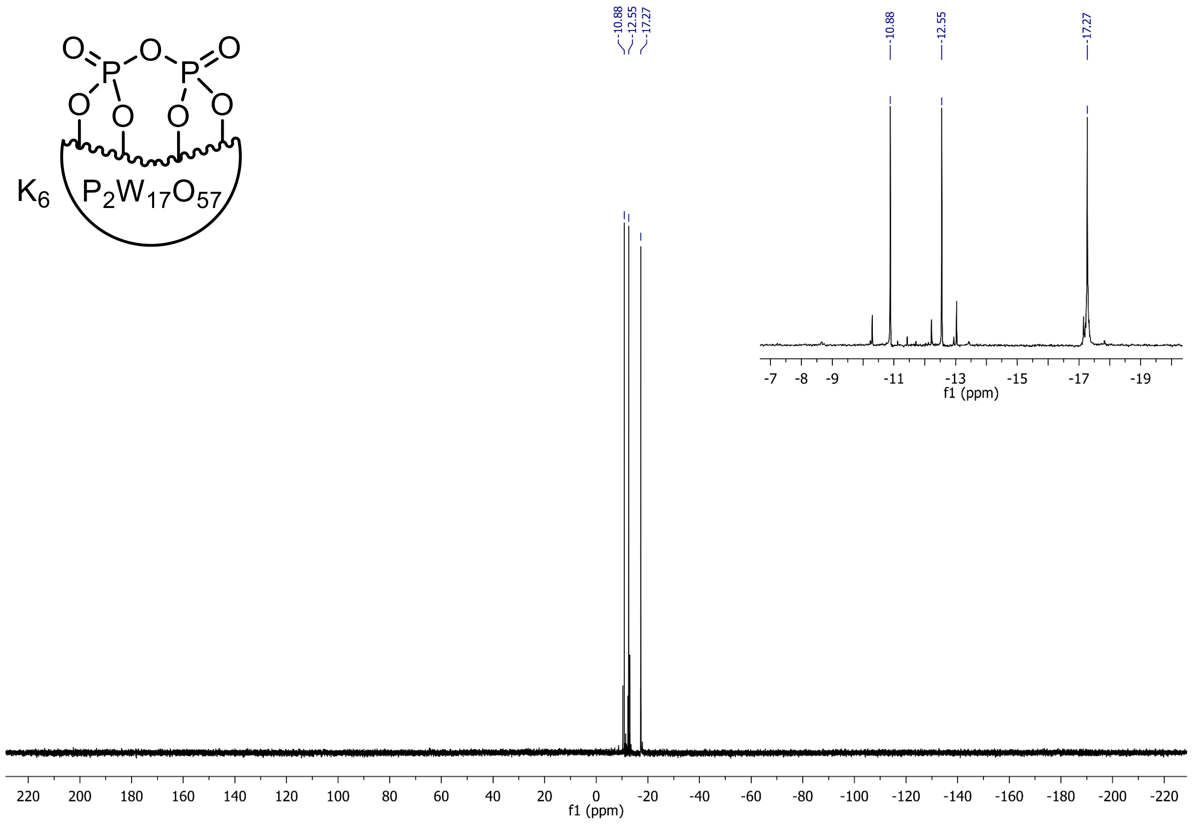

SI Figure 12. ^{31}P NMR (202 MHz, D_2O) of $K_6[P_2W_{17}O_{57}(P_2O_7)]$ (**2**).

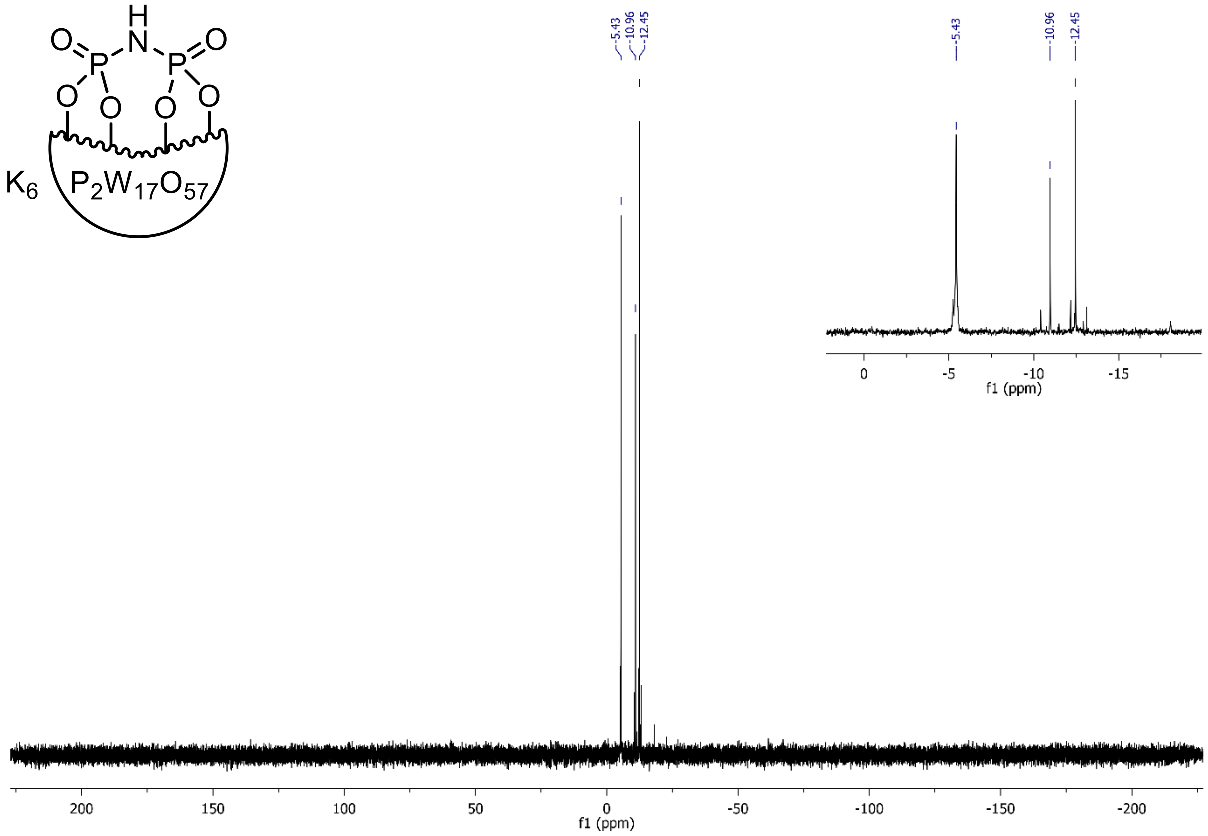

SI Figure 13. $^{31}P\{^{1}H\}$ NMR (162 MHz, CD_3CN) of $K_6[P_2W_{17}O_{57}(P_2O_6NH)]$ (**3**).

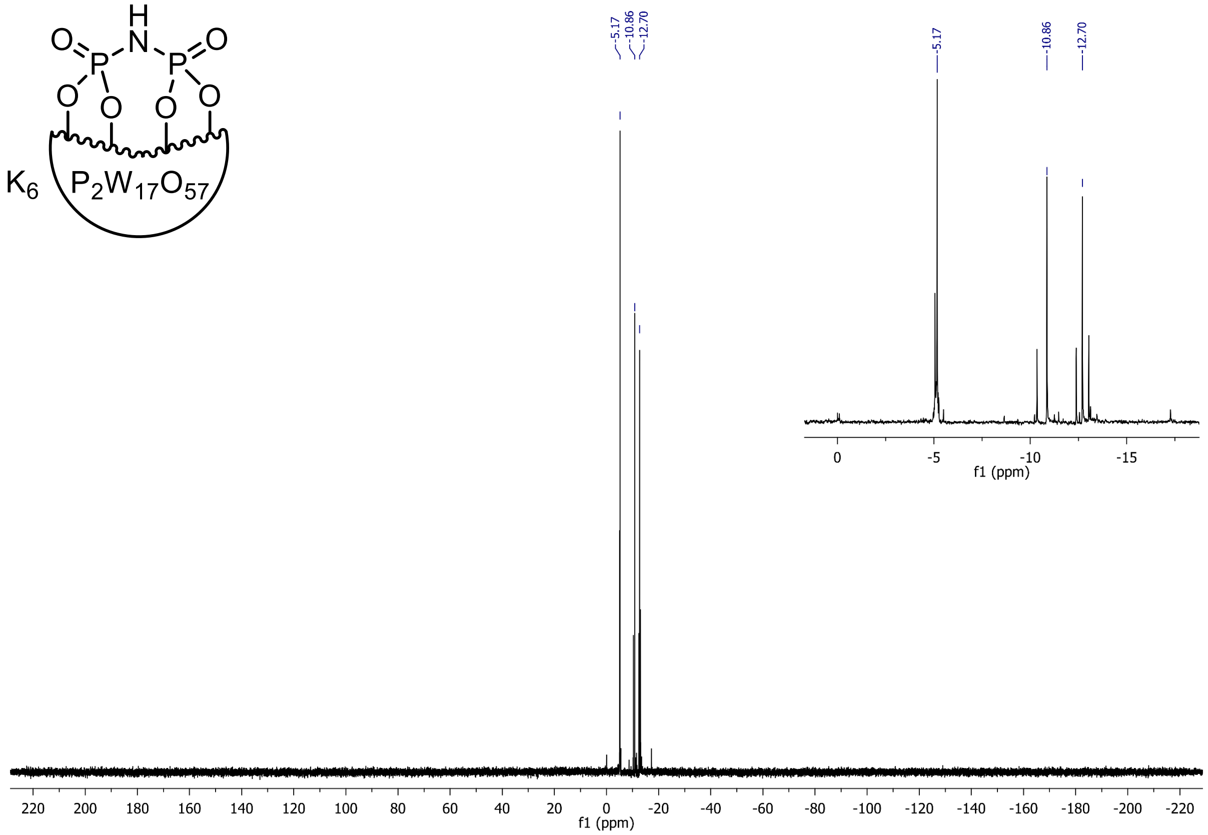

SI Figure 14. $^{31}P\{^{1}H\}$ NMR (202 MHz, D_2O) of $K_6[P_2W_{17}O_{57}(P_2O_6NH)]$ (**3**).

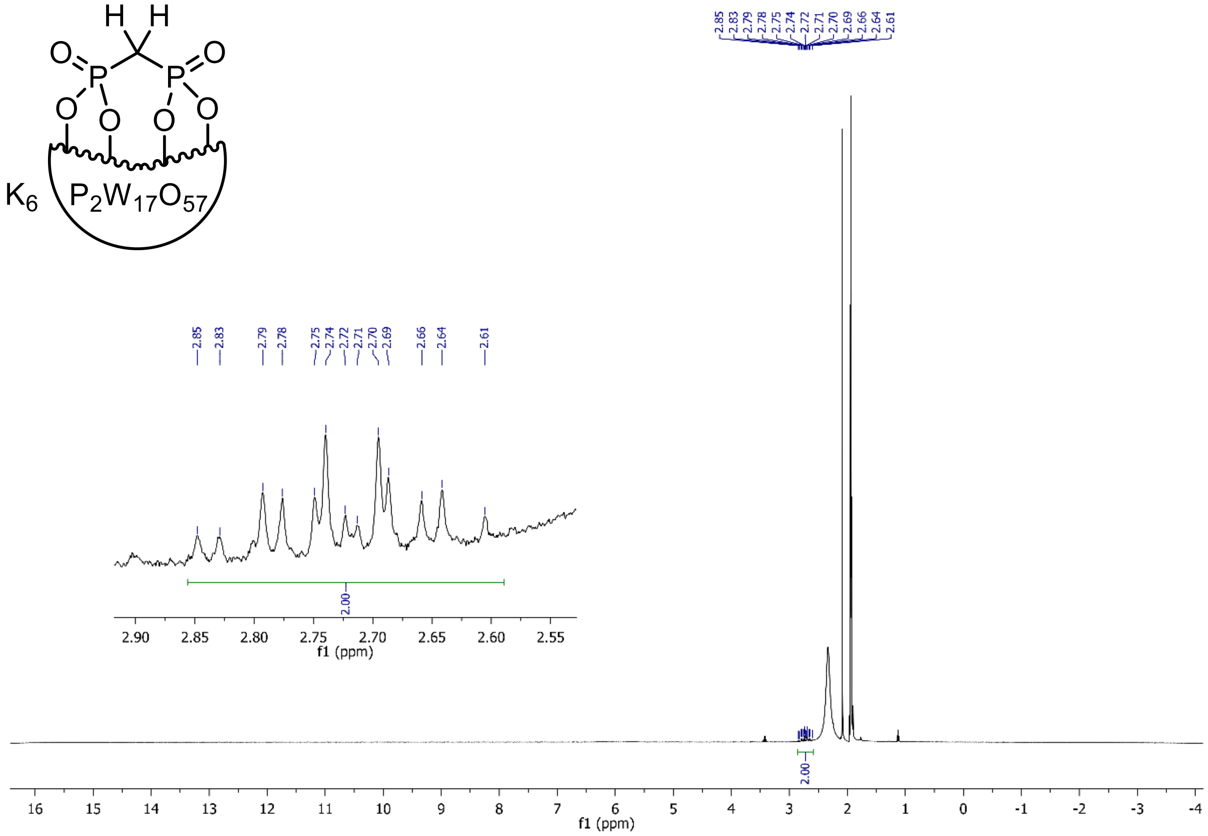

SI Figure 15. ^{1}H NMR (400 MHz, CD_3CN) of $K_6[P_2W_{17}O_{57}(P_2O_6CH_2)]$ (**4**).

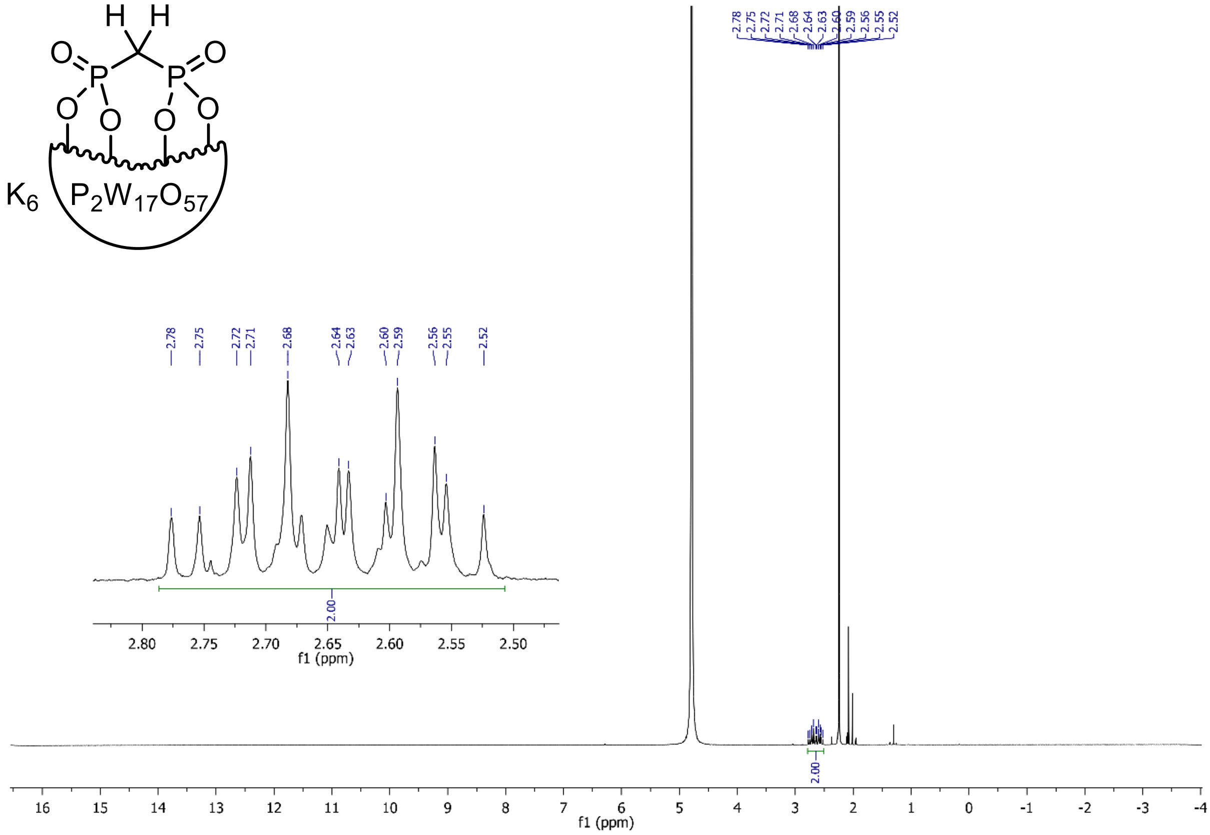

SI Figure 16. ^{1}H NMR (500 MHz, D_2O) of $K_6[P_2W_{17}O_{57}(P_2O_6CH_2)]$ (**4**).

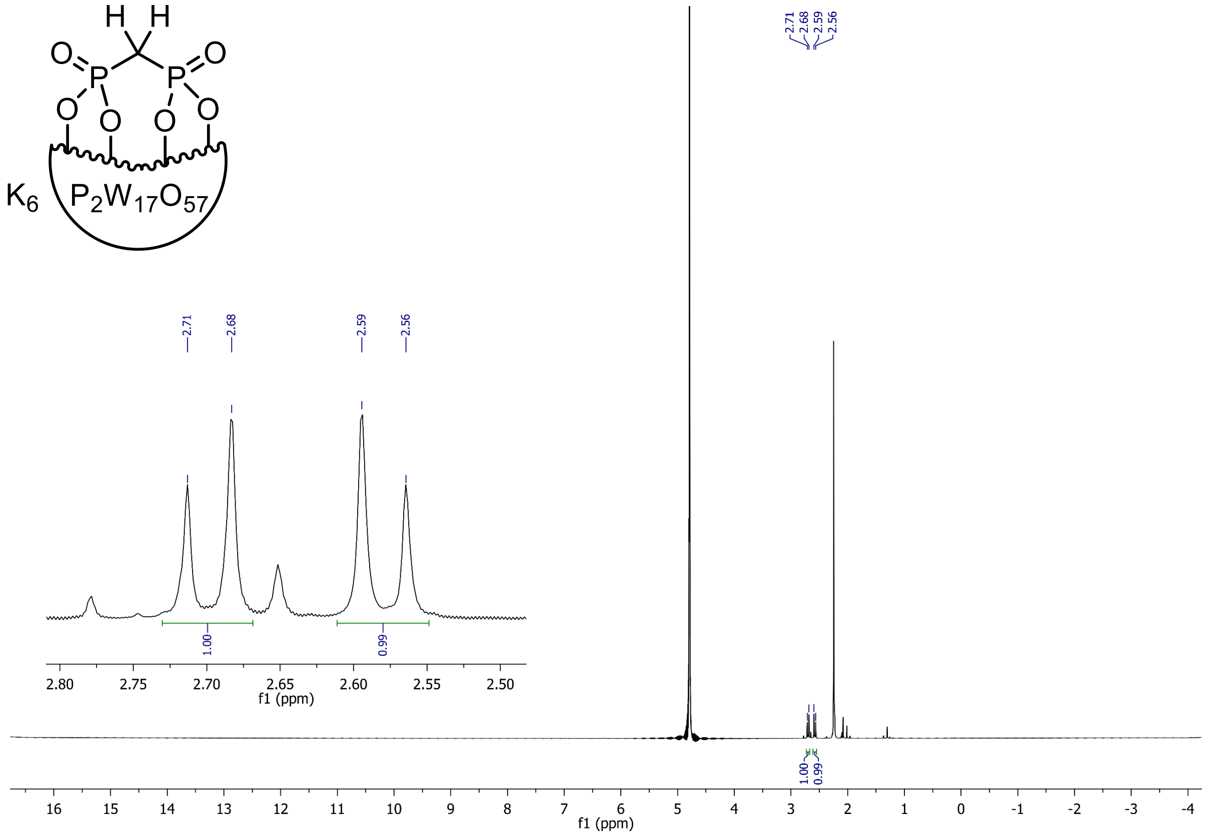

SI Figure 17. $^{1}H\{^{31}P\}$ NMR (500 MHz, D_2O) of $K_6[P_2W_{17}O_{57}(P_2O_6CH_2)]$ (**4**).

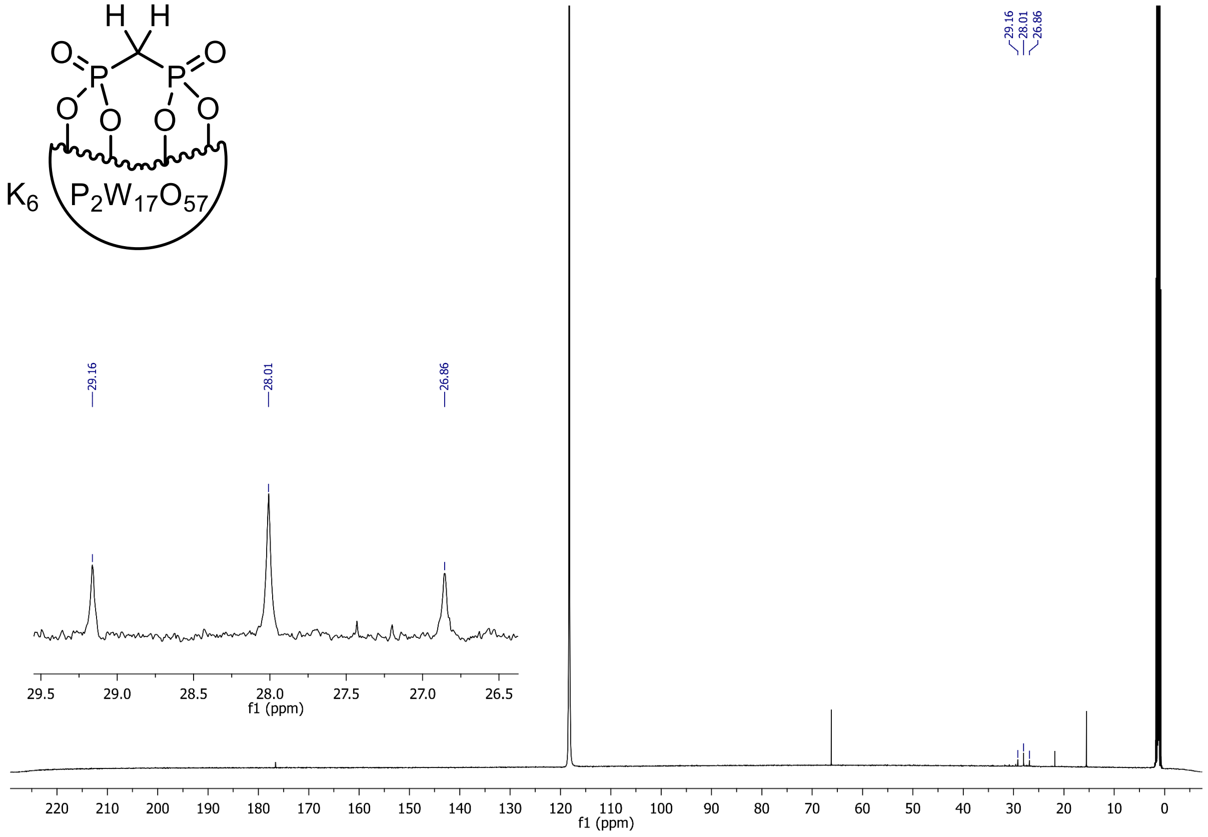

SI Figure 18. ^{13}C NMR (126 MHz, CD_3CN) of $K_6[P_2W_{17}O_{57}(P_2O_6CH_2)]$ (**4**).

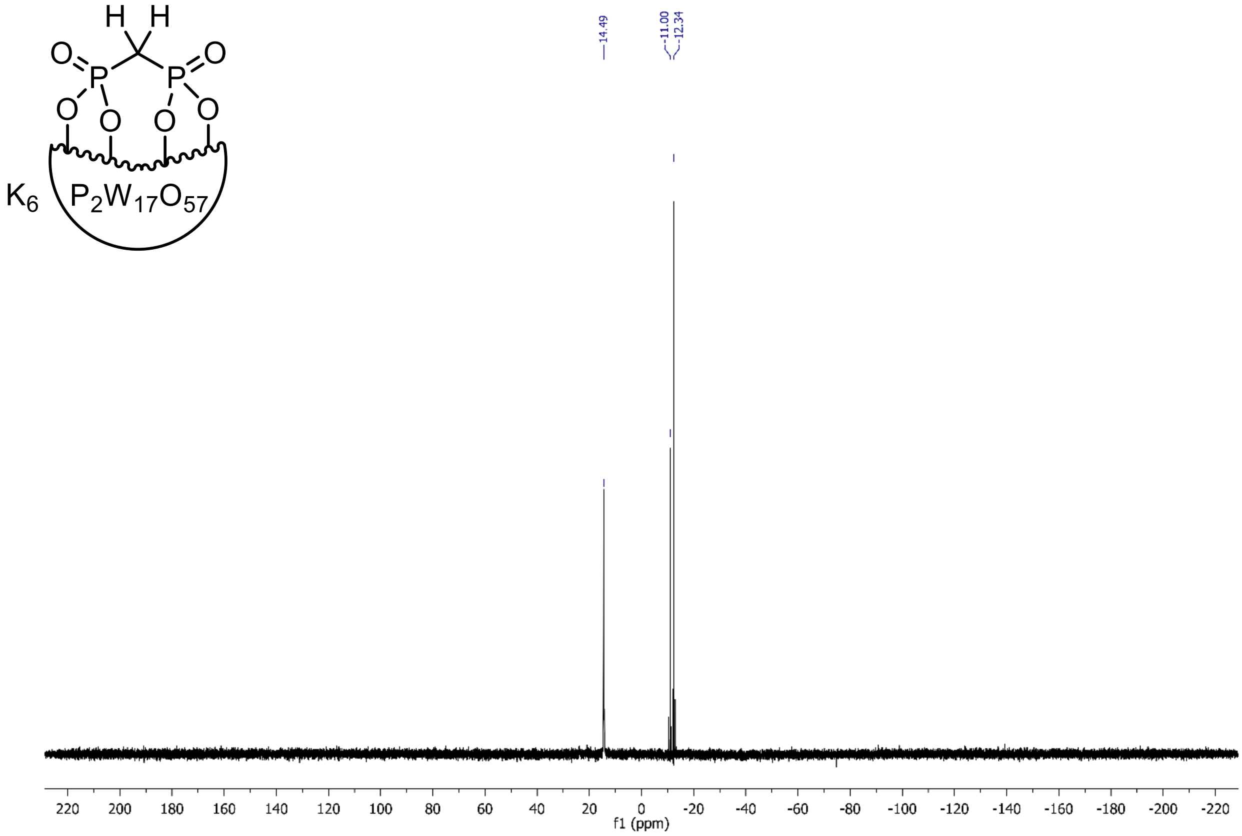

SI Figure 19. $^{31}P\{^{1}H\}$ NMR (202 MHz, CD_3CN) of $K_6[P_2W_{17}O_{57}(P_2O_6CH_2)]$ (**4**).

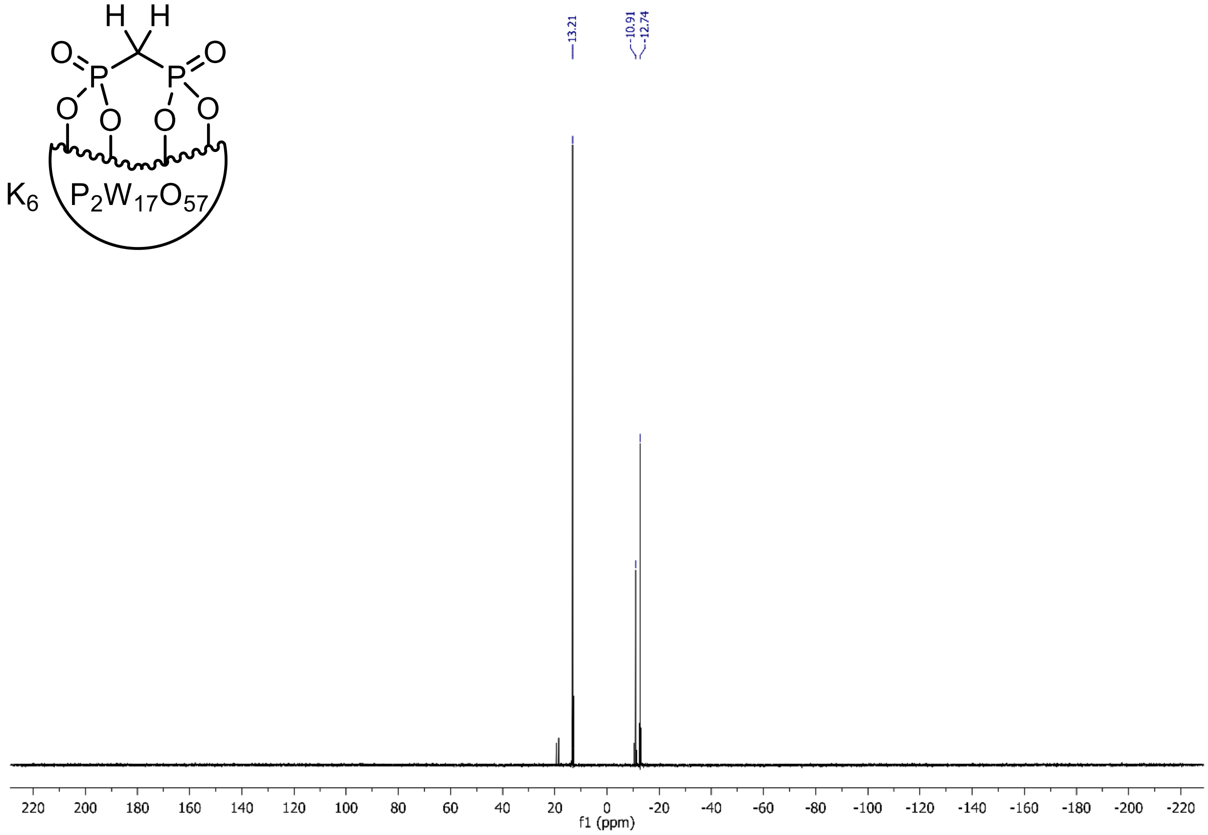

SI Figure 20. $^{31}P\{^{1}H\}$ NMR (202 MHz, D_2O) of $K_6[P_2W_{17}O_{57}(P_2O_6CH_2)]$ (**4**).

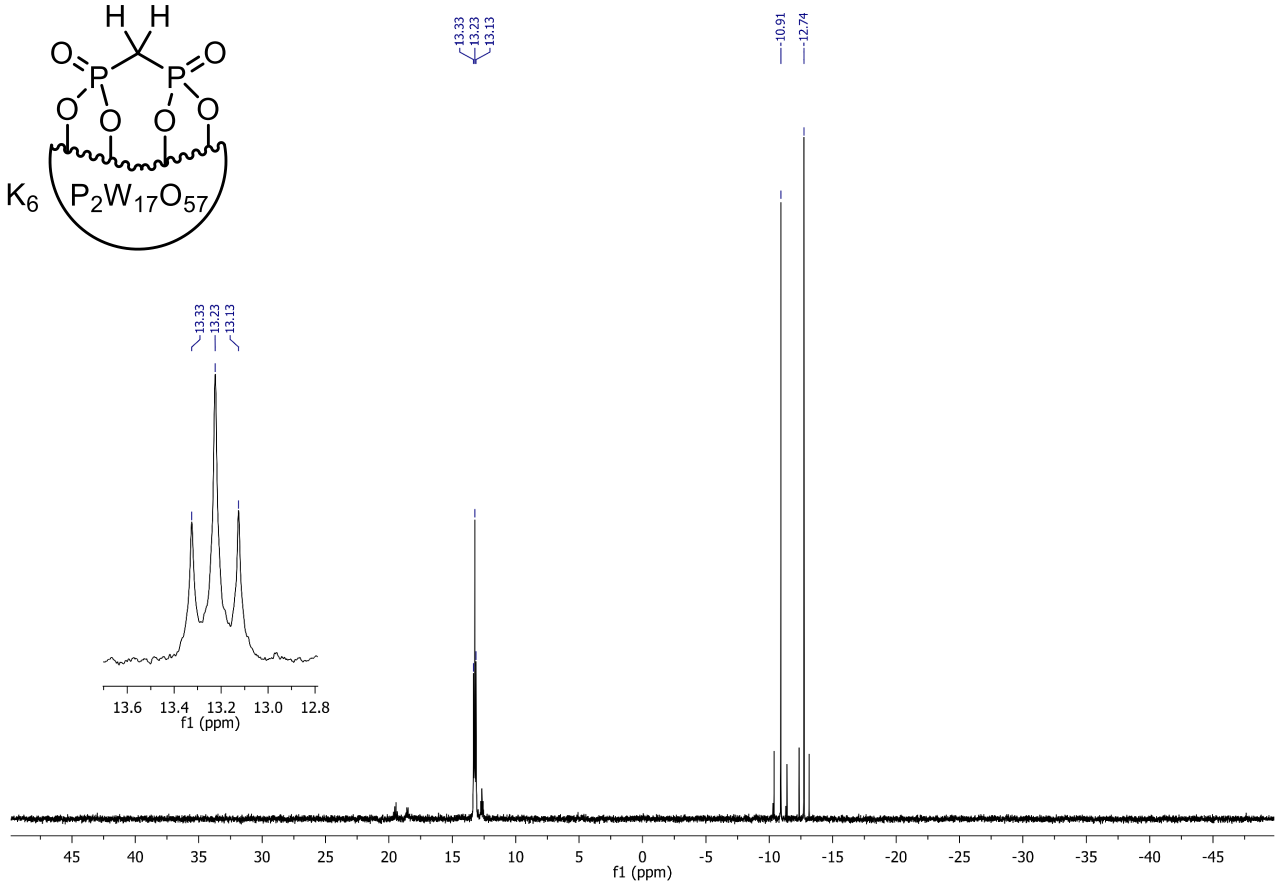

SI Figure 21. ^{31}P NMR (202 MHz, D_2O) of $K_6[P_2W_{17}O_{57}(P_2O_6CH_2)]$ (**4**).

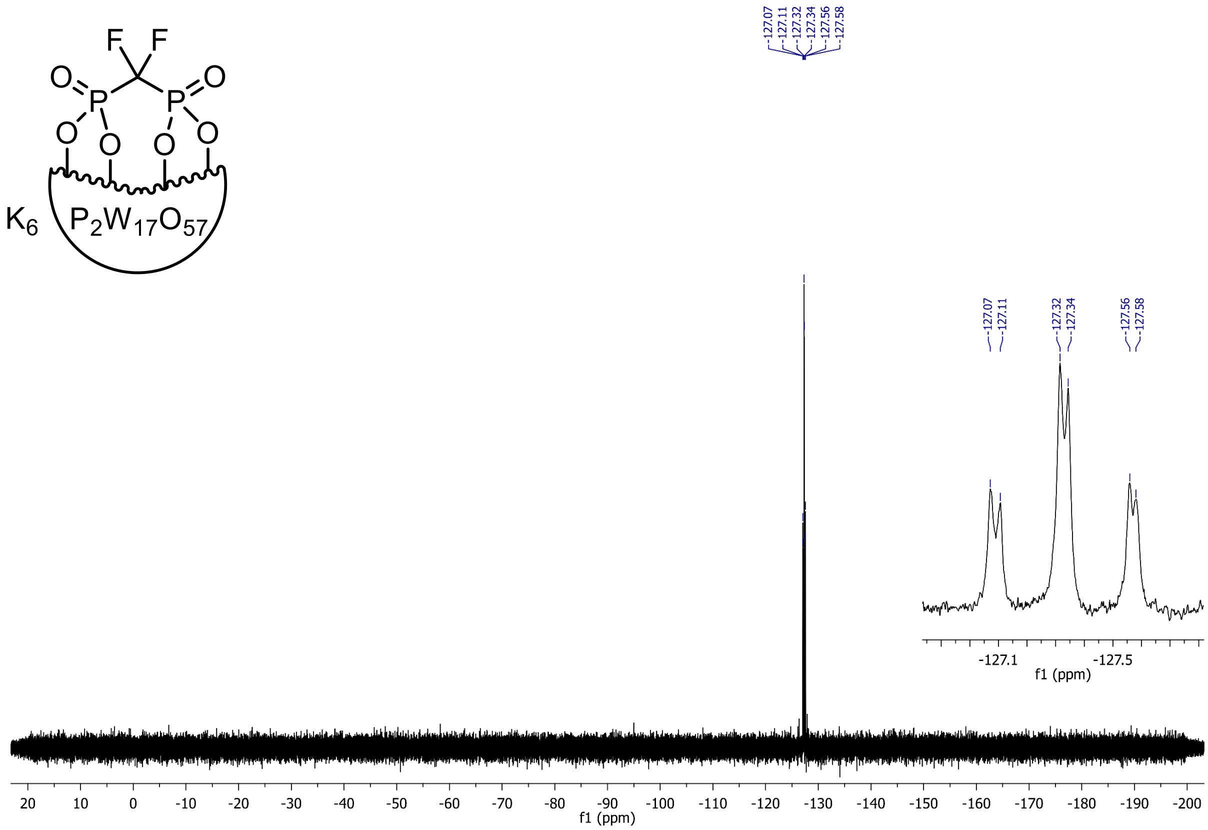

SI Figure 22. ^{19}F NMR (377 MHz, CD_3CN) of $K_6[P_2W_{17}O_{57}(P_2O_6CF_2)]$ (**5**).

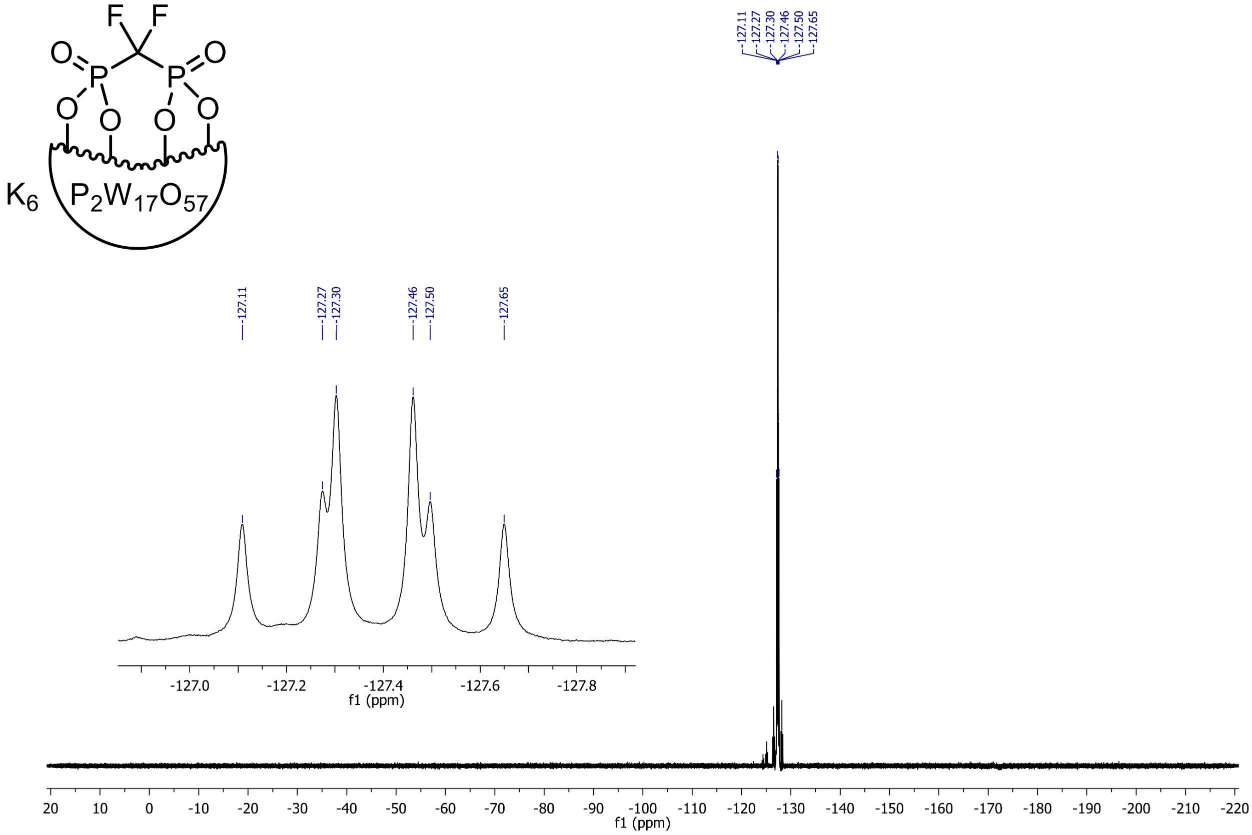

SI Figure 23. ^{19}F NMR (470 MHz, Acetone-d_6) of $K_6[P_2W_{17}O_{57}(P_2O_6CF_2)]$ (**5**).

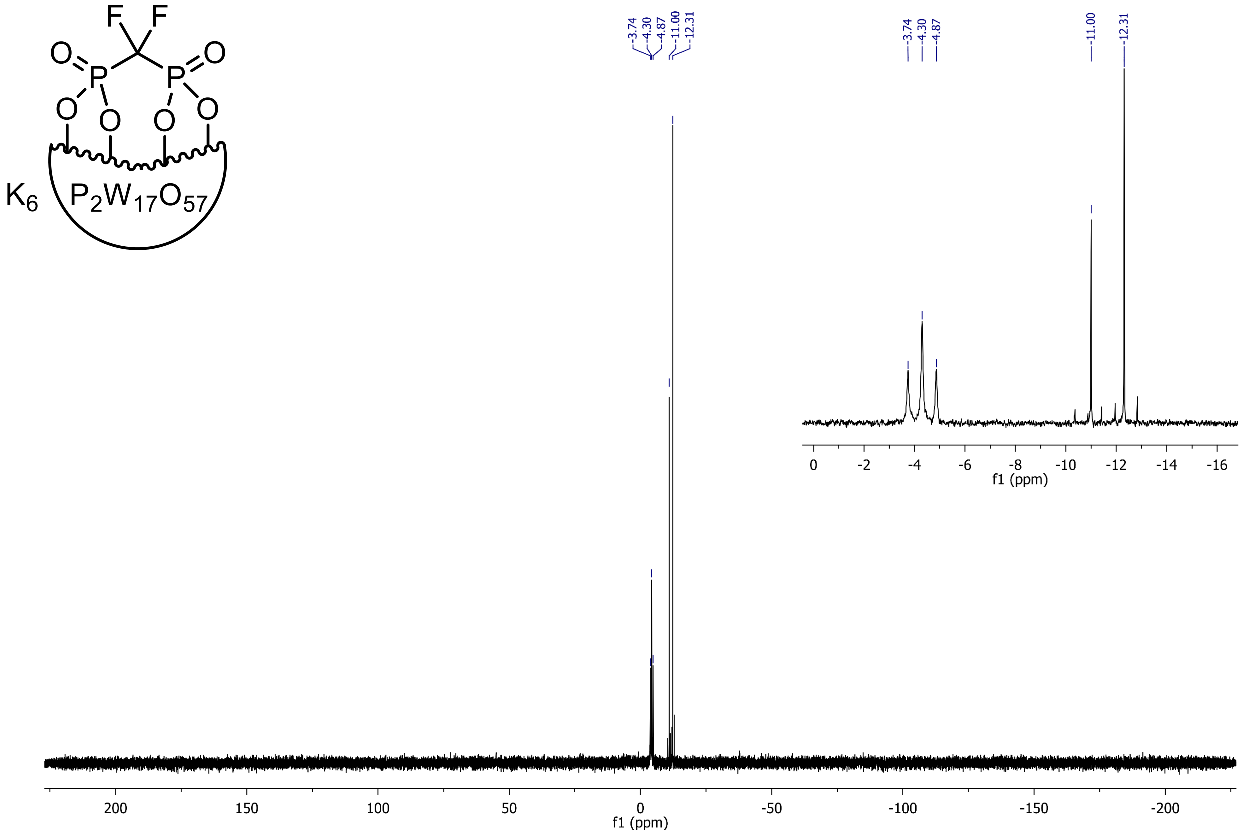

SI Figure 24. ^{31}P NMR (162 MHz, CD_3CN) of $K_6[P_2W_{17}O_{57}(P_2O_6CF_2)]$ (**5**).

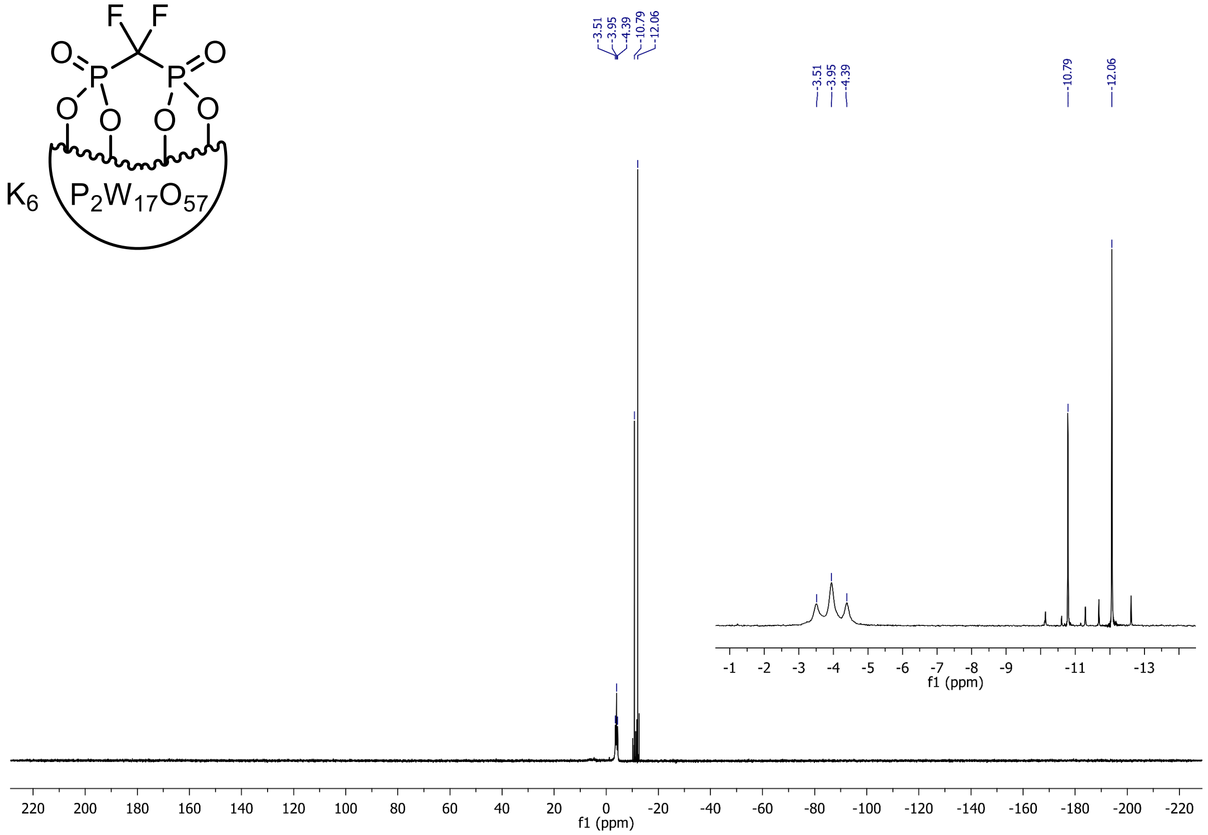

SI Figure 25. ^{31}P NMR (202 MHz, Acetone-d_6) of $K_6[P_2W_{17}O_{57}(P_2O_6CF_2)]$ (**5**).

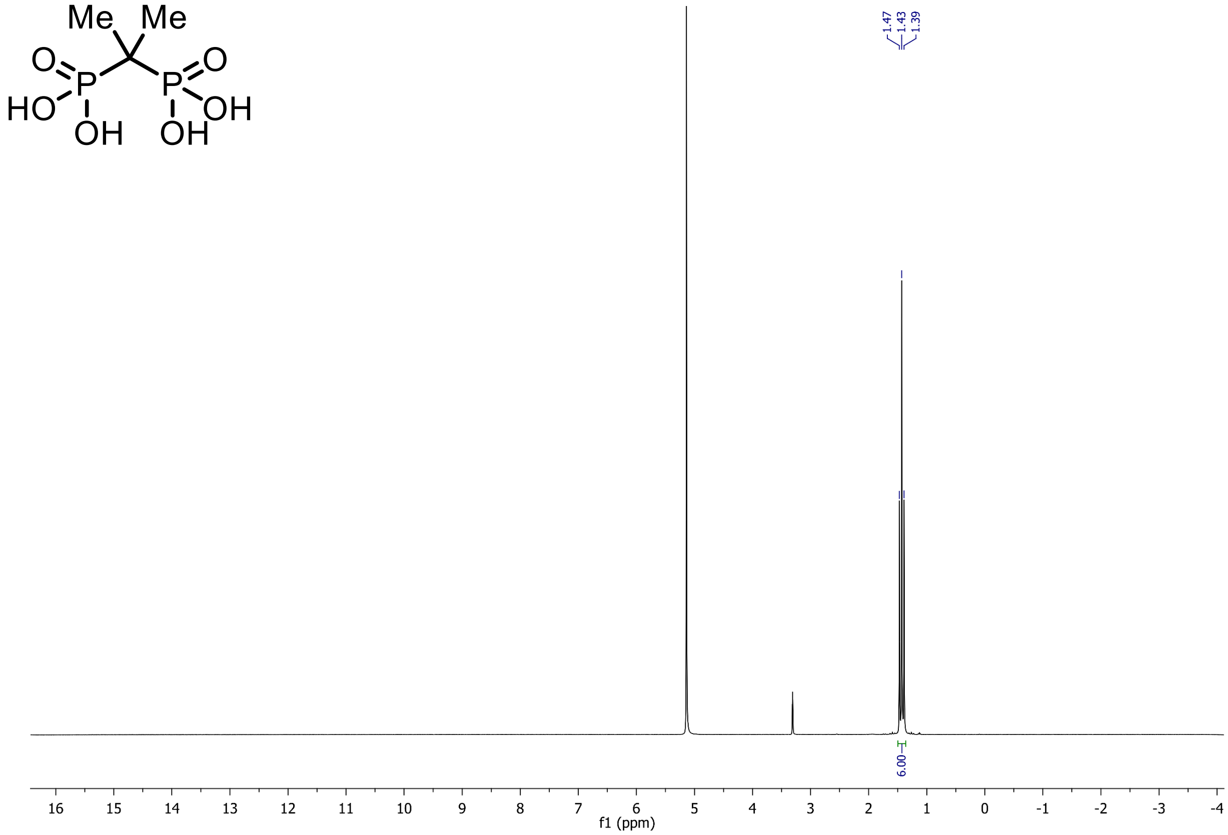

SI Figure 26. ^{1}H NMR (400 MHz, CD_3OD) of propane-2,2-diyldiphosphonic acid (**S7**).

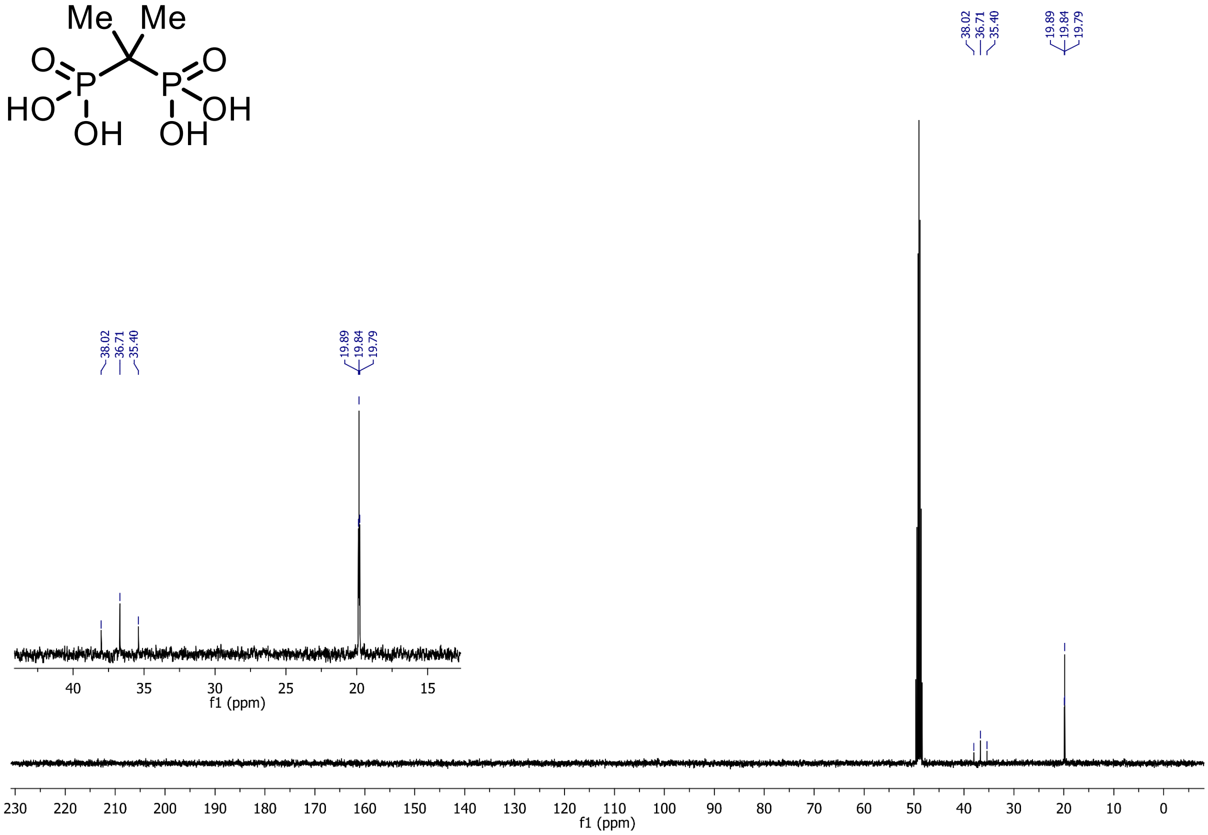

SI Figure 27. ^{13}C NMR (101 MHz, CD_3OD) of propane-2,2-diyldiphosphonic acid (**S7**).

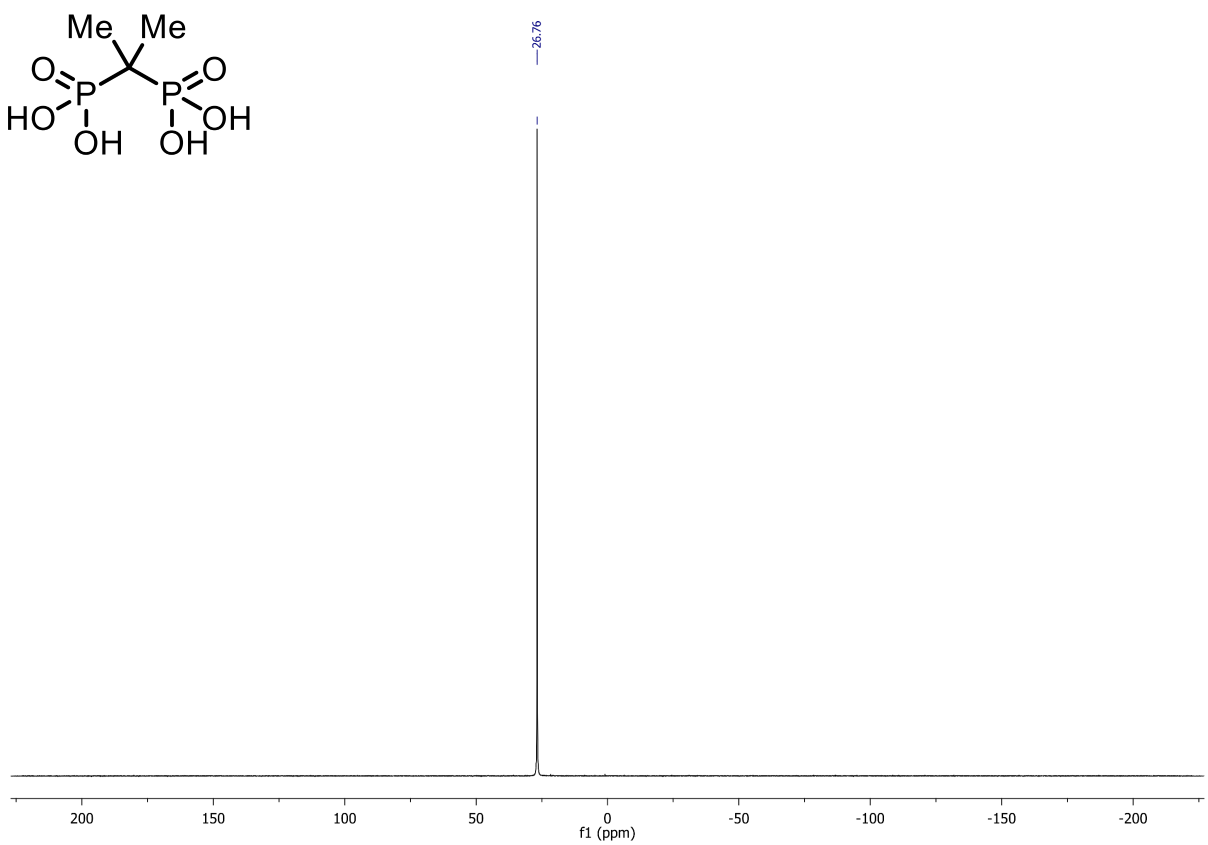

SI Figure 28. $^{31}P\{^{1}H\}$ NMR (162 MHz, CD_3OD) of propane-2,2-diyldiphosphonic acid (**S7**).

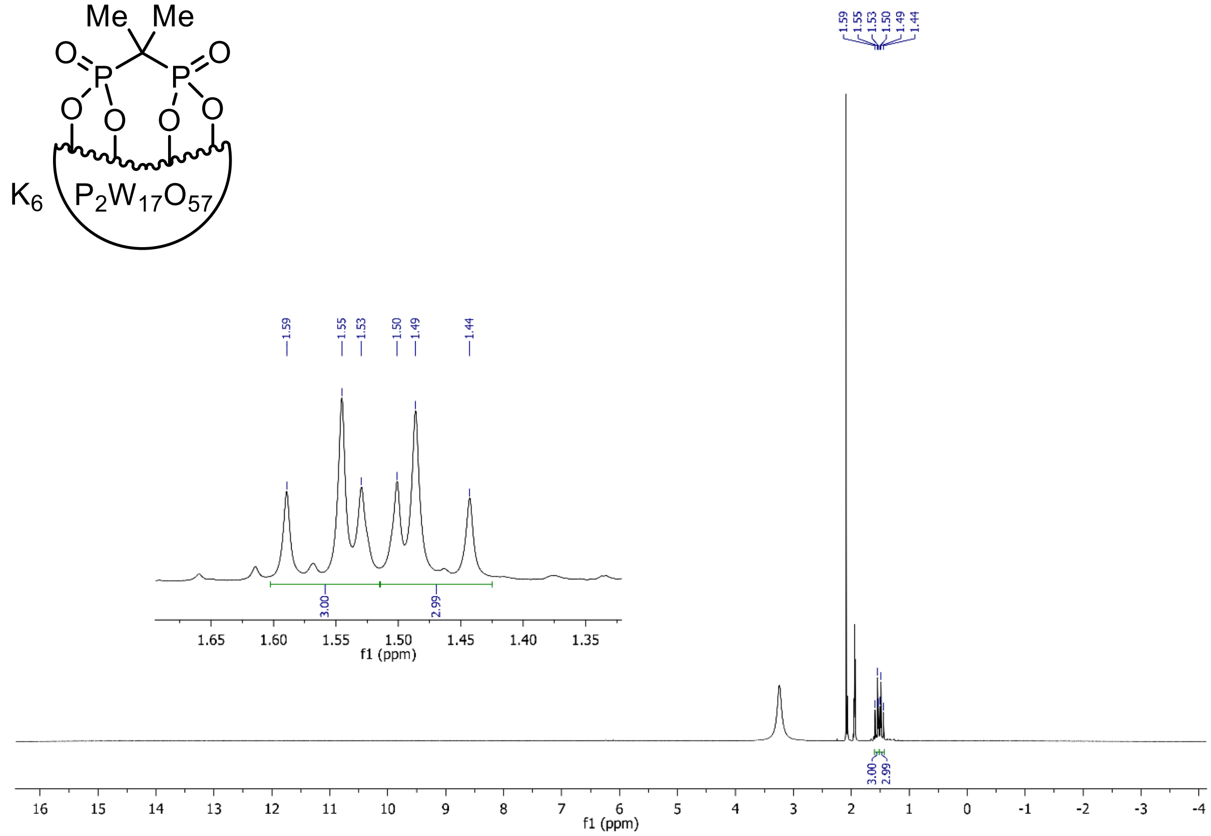

SI Figure 29. ¹H NMR (400 MHz, CD$_3$CN) of K$_6$[P$_2$W$_{17}$O$_{57}$(P$_2$O$_6$C(CH$_3$)$_2$)] (**6**).

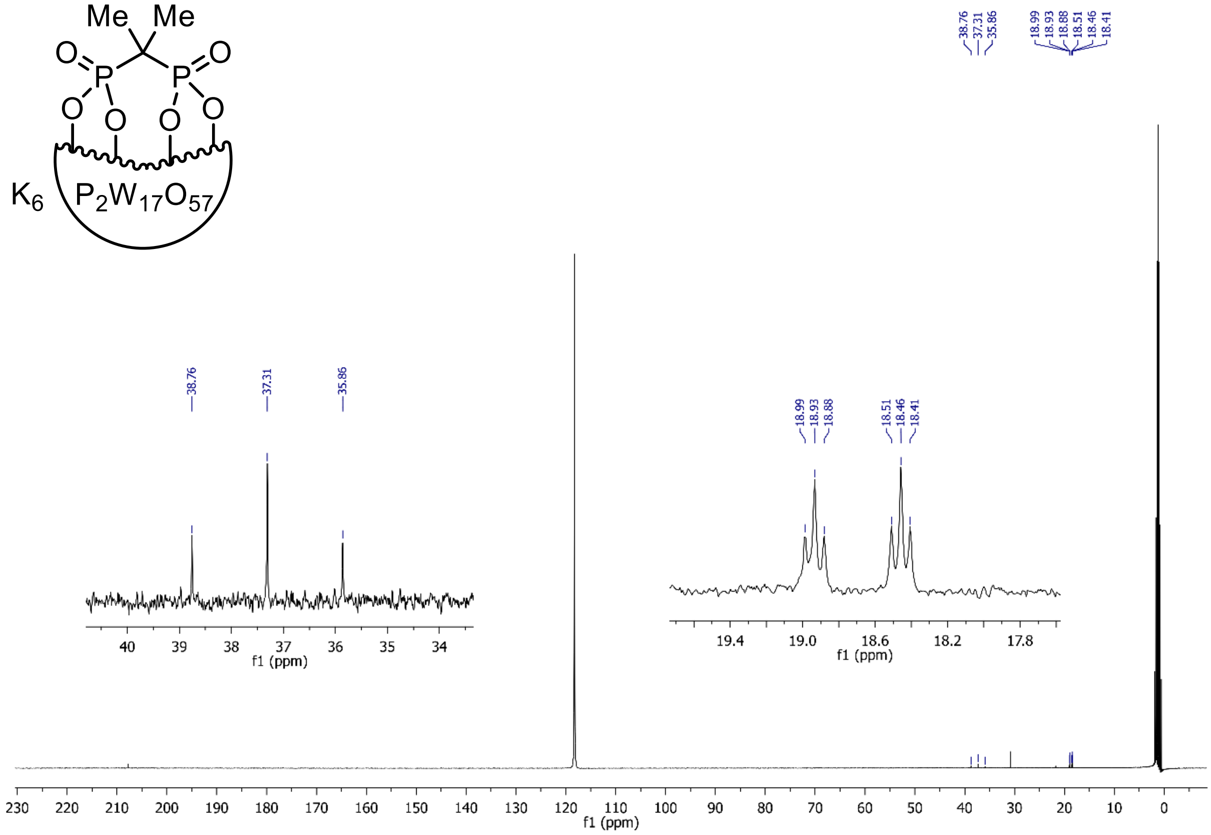

SI Figure 30. ¹³C NMR (101 MHz, CD$_3$CN) of K$_6$[P$_2$W$_{17}$O$_{57}$(P$_2$O$_6$C(CH$_3$)$_2$)] (**6**).

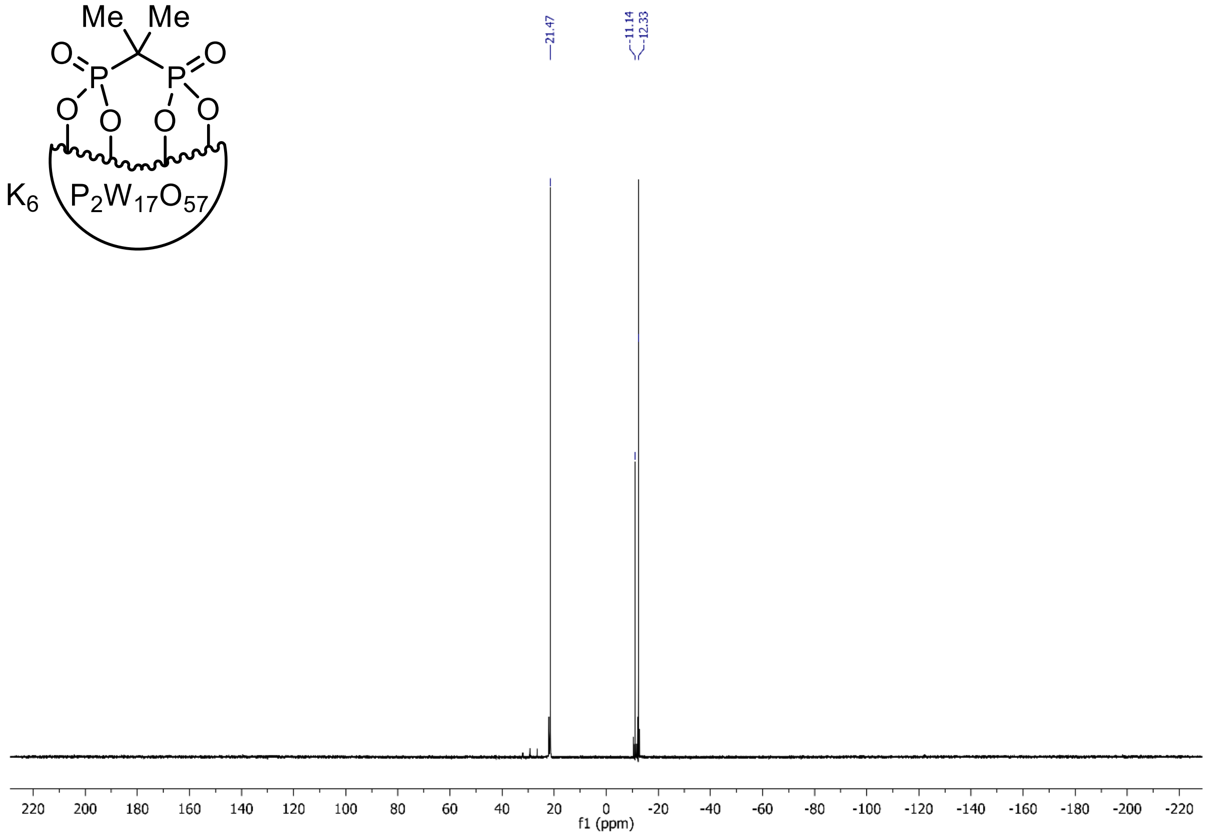

SI Figure 31. $^{31}P\{^1H\}$ NMR (202 MHz, CD_3CN) of $K_6[P_2W_{17}O_{57}(P_2O_6C(CH_3)_2)]$ (**6**).

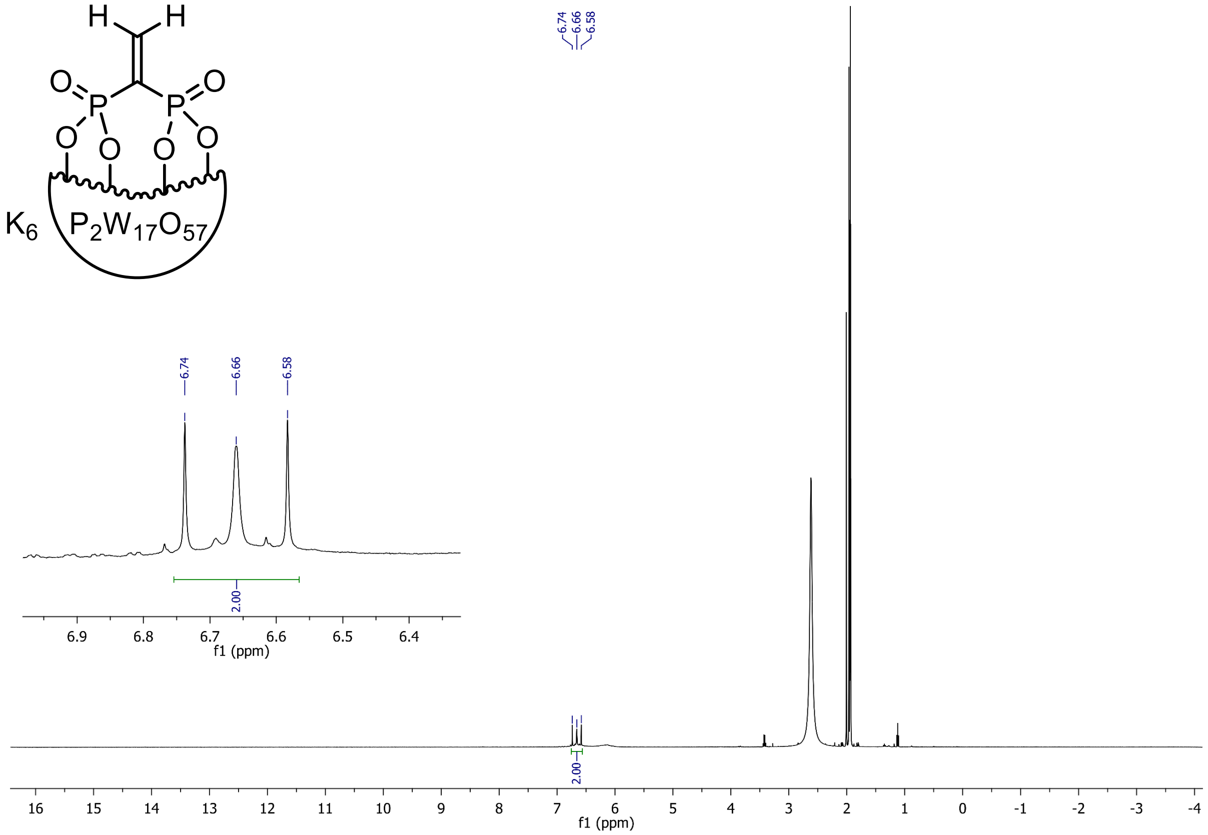

SI Figure 32. ^{1}H NMR (500 MHz, CD_3CN) of $K_6[P_2W_{17}O_{57}(P_2O_6CCH_2)]$ (**7**).

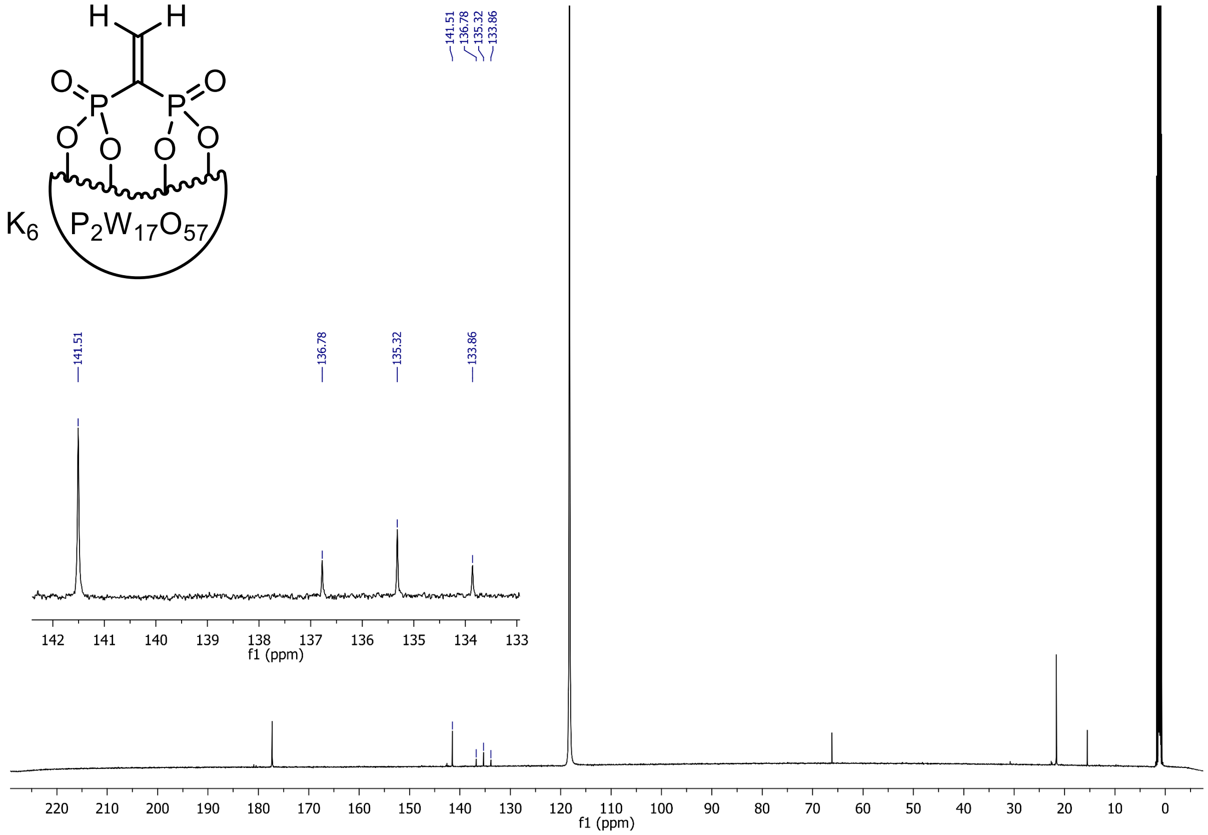

SI Figure 33. ^{13}C NMR (126 MHz, CD_3CN) of $K_6[P_2W_{17}O_{57}(P_2O_6CCH_2)]$ (**7**).

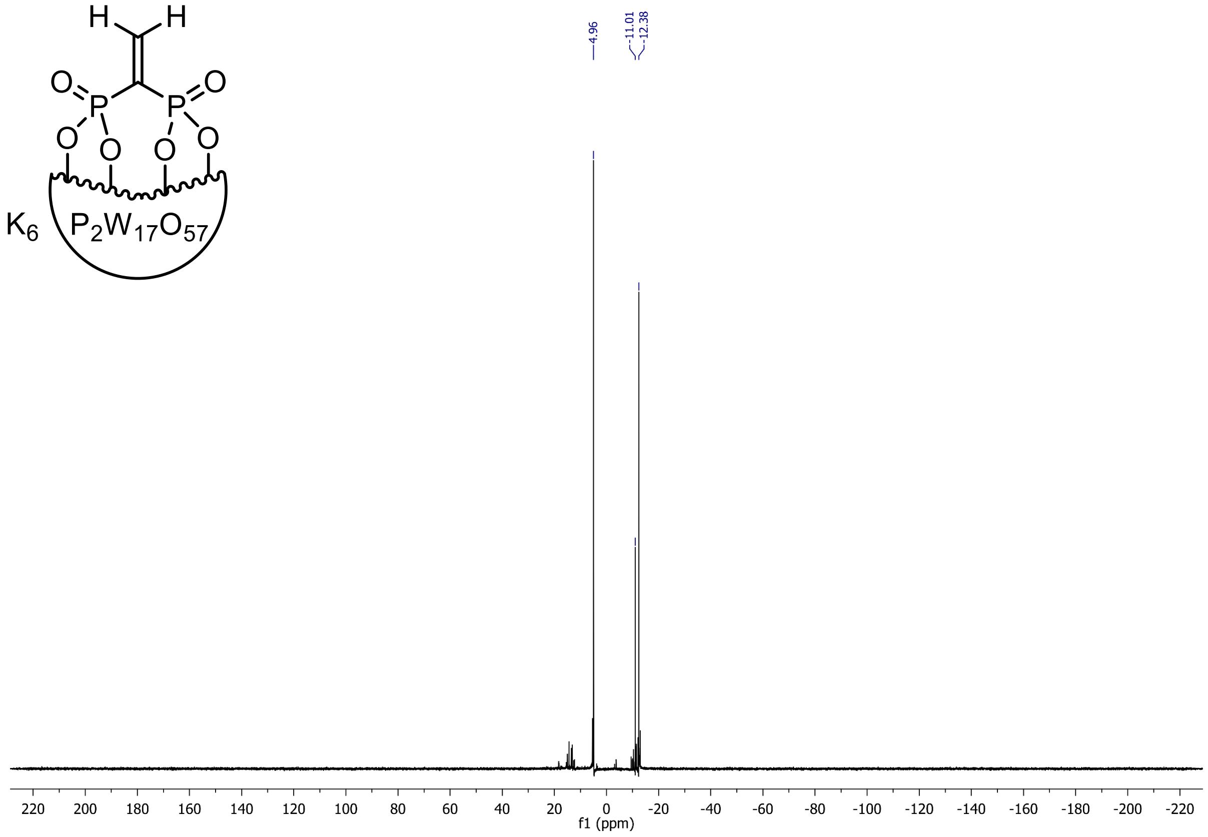

SI Figure 34. $^{31}P\{^{1}H\}$ NMR (202 MHz, CD_3CN) of $K_6[P_2W_{17}O_{57}(P_2O_6CCH_2)]$ (**7**).

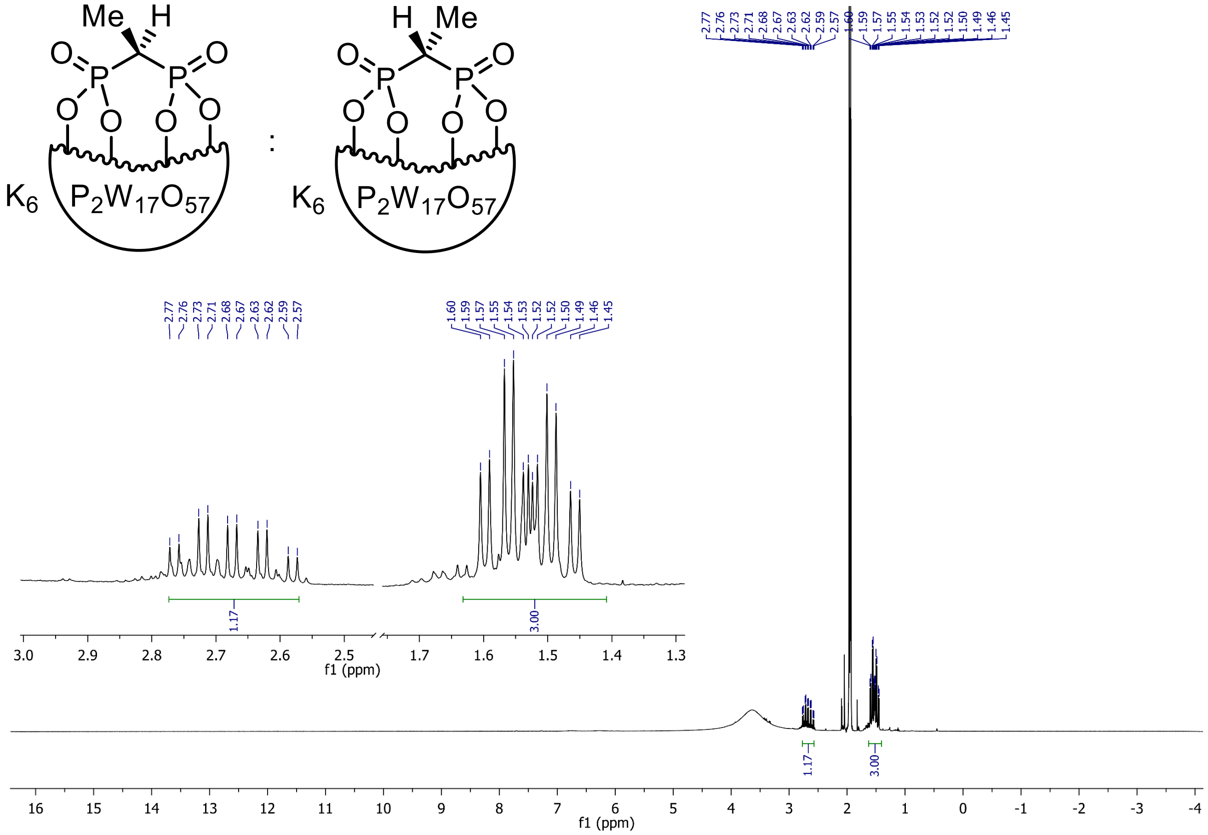

SI Figure 35. ^{1}H NMR (500 MHz, CD_3CN) of $K_6[P_2W_{17}O_{57}(P_2O_6CHCH_3)]$ (**8**) as a 1:1 mixture of diastereomers.

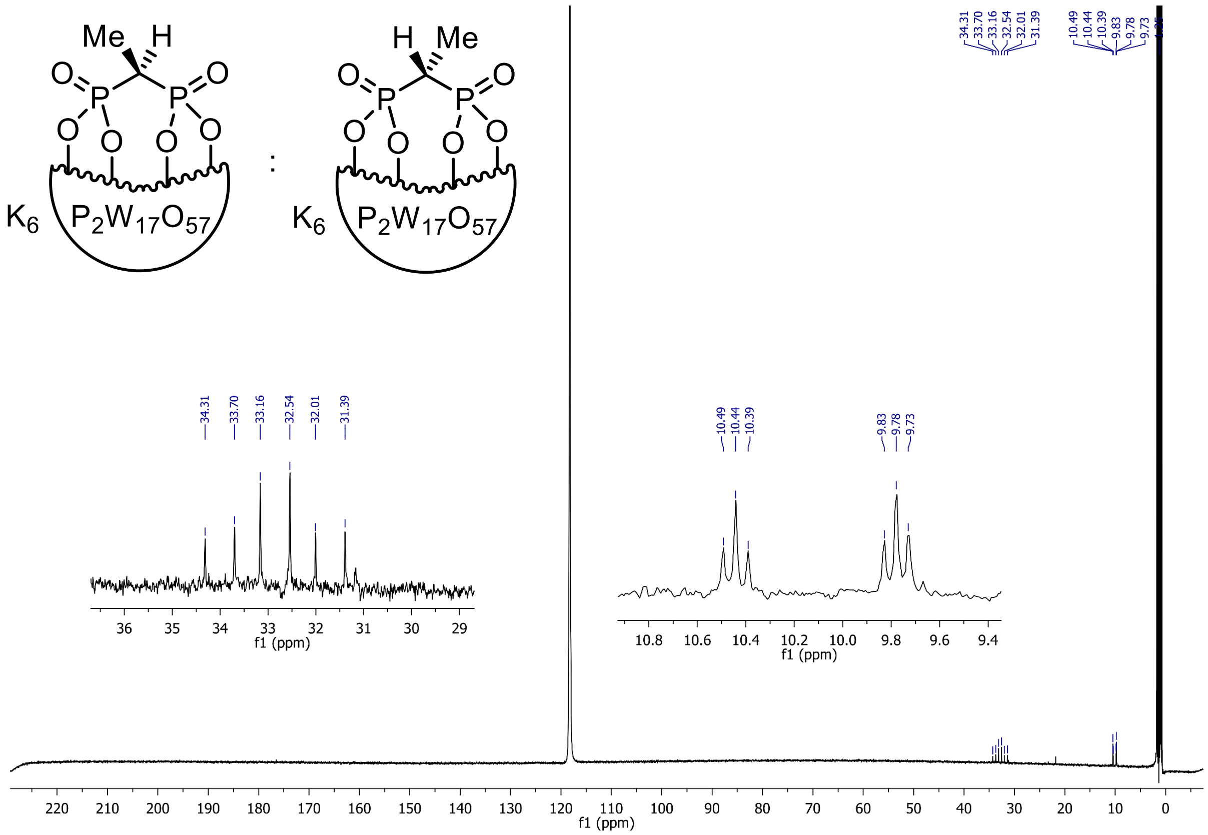

SI Figure 36. ^{13}C NMR (126 MHz, CD_3CN) of $K_6[P_2W_{17}O_{57}(P_2O_6CHCH_3)]$ (**8**) as a 1:1 mixture of diastereomers.

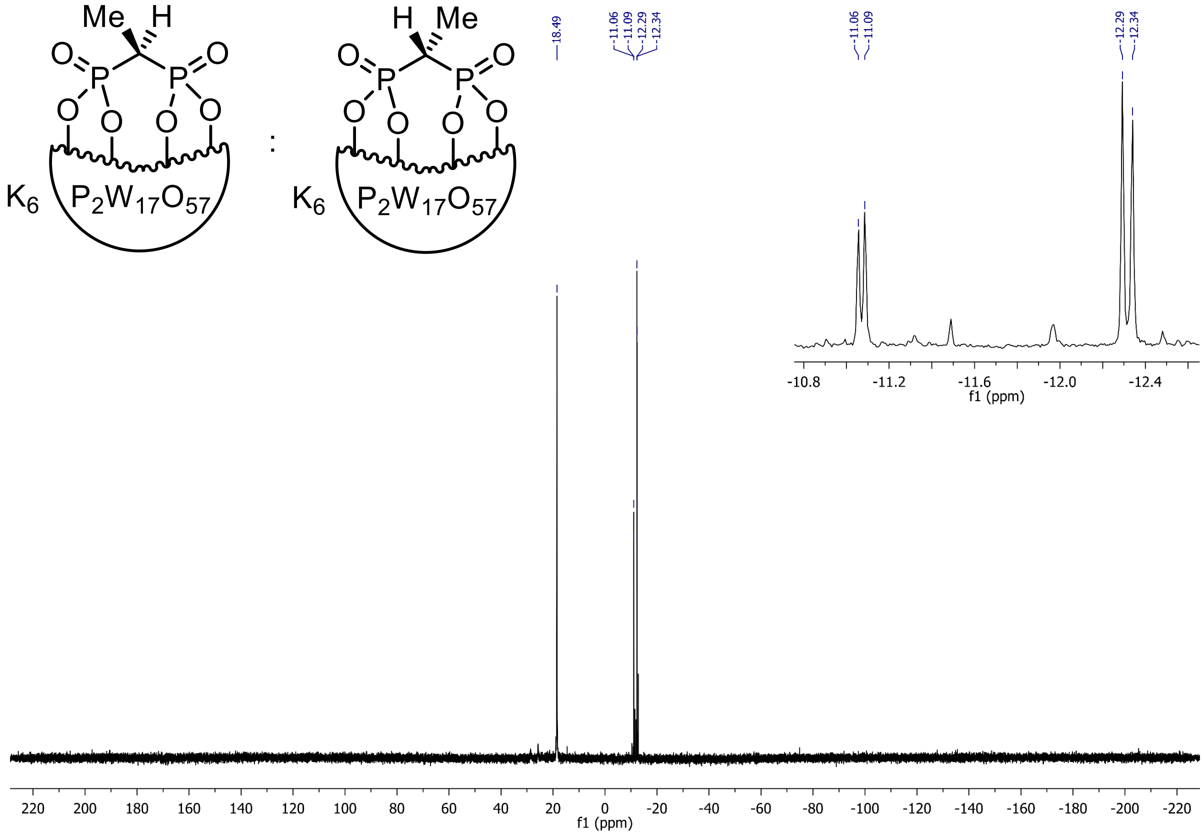

SI Figure 37. $^{31}P\{^{1}H\}$ NMR (202 MHz, CD_3CN) of $K_6[P_2W_{17}O_{57}(P_2O_6CHCH_3)]$ (**8**) as a 1:1 mixture of diastereomers.

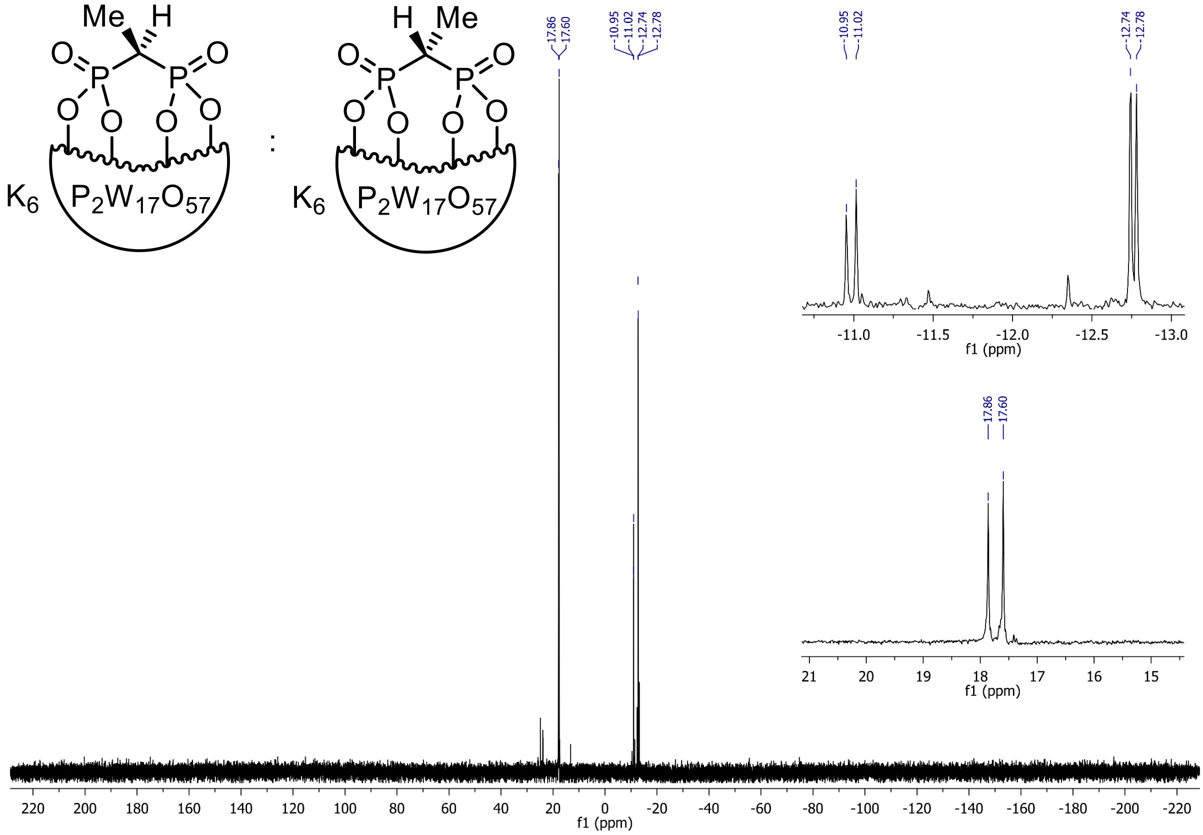

SI Figure 38. $^{31}P\{^{1}H\}$ NMR (202 MHz, D_2O) of $K_6[P_2W_{17}O_{57}(P_2O_6CHCH_3)]$ (**8**) as a 1:1 mixture of diastereomers.

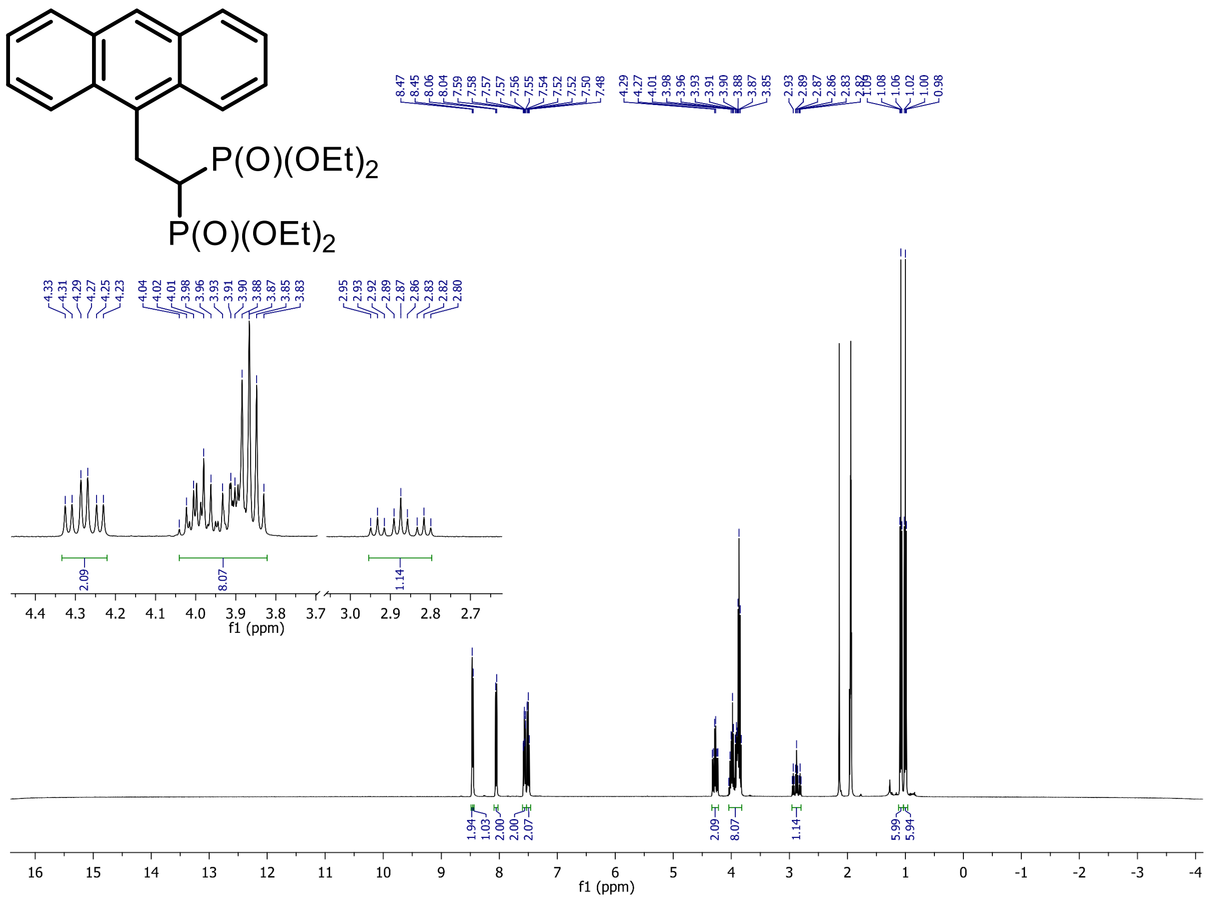

SI Figure 39. ^{1}H NMR (400 MHz, CD_3CN) of tetraethyl (2-(anthracen-9-yl)ethane-1,1-diyl)bis(phosphonate) (**S8**).

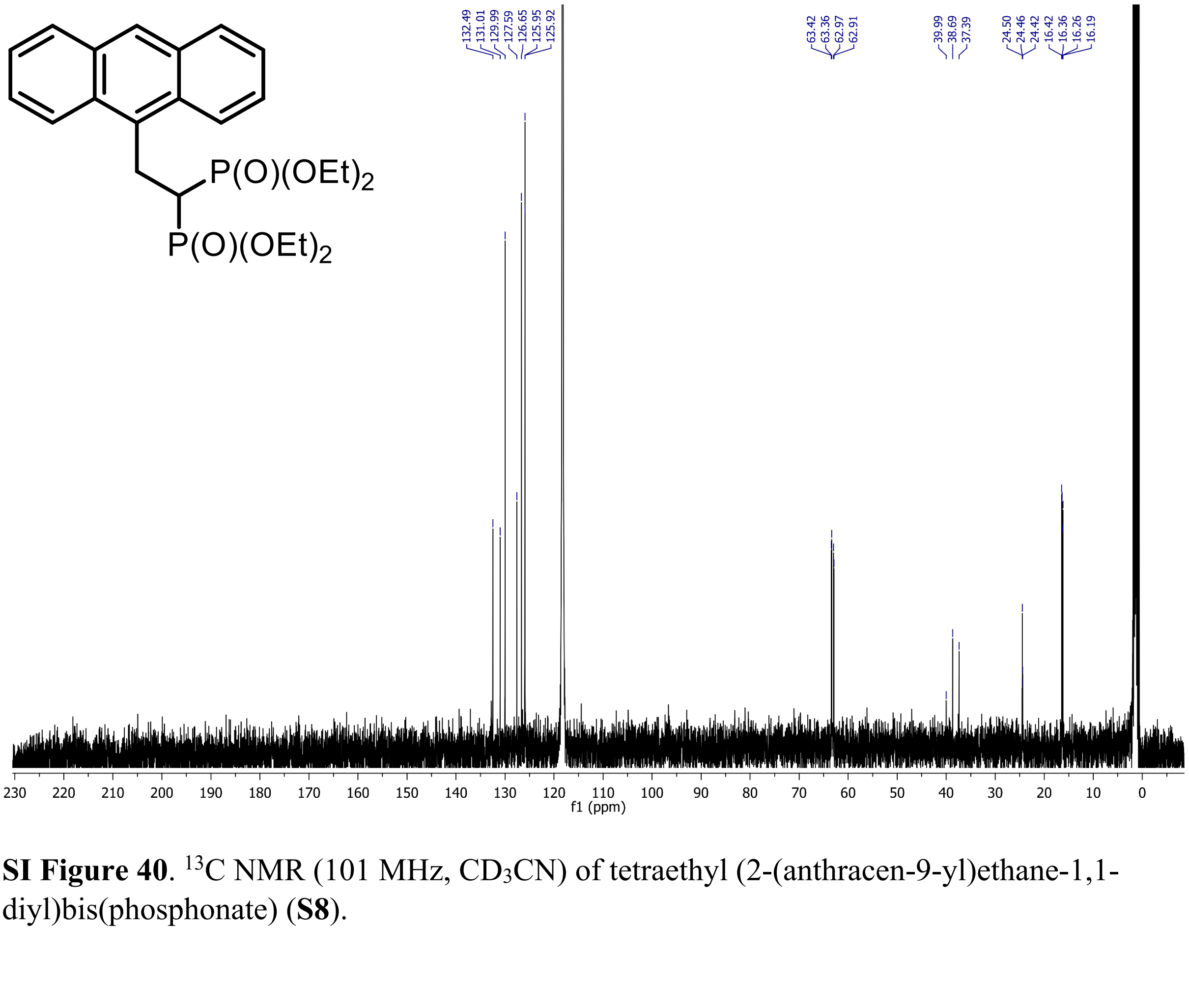

SI Figure 40. ^{13}C NMR (101 MHz, CD_3CN) of tetraethyl (2-(anthracen-9-yl)ethane-1,1-diyl)bis(phosphonate) (**S8**).

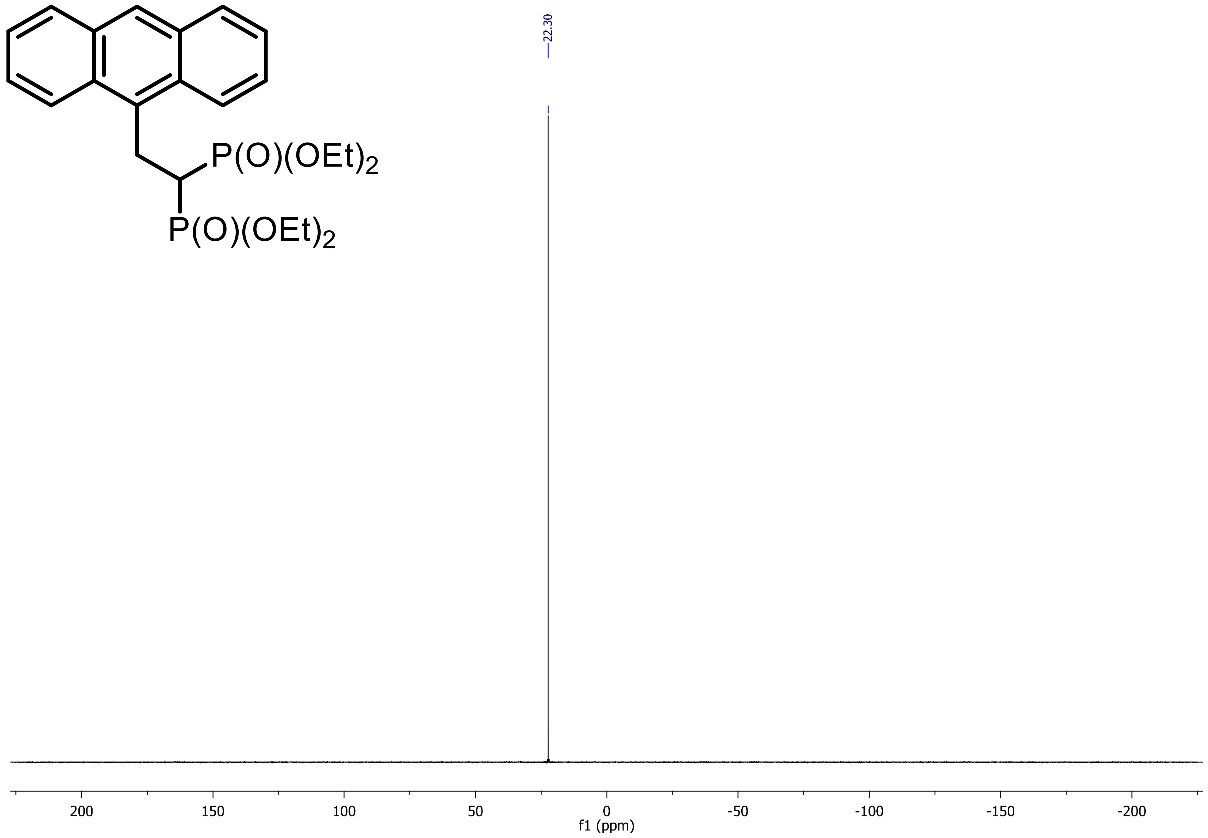

SI Figure 41. $^{31}P\{^1H\}$ NMR (162 MHz, CD_3CN) of tetraethyl (2-(anthracen-9-yl)ethane-1,1-diyl)bis(phosphonate) (**S8**).

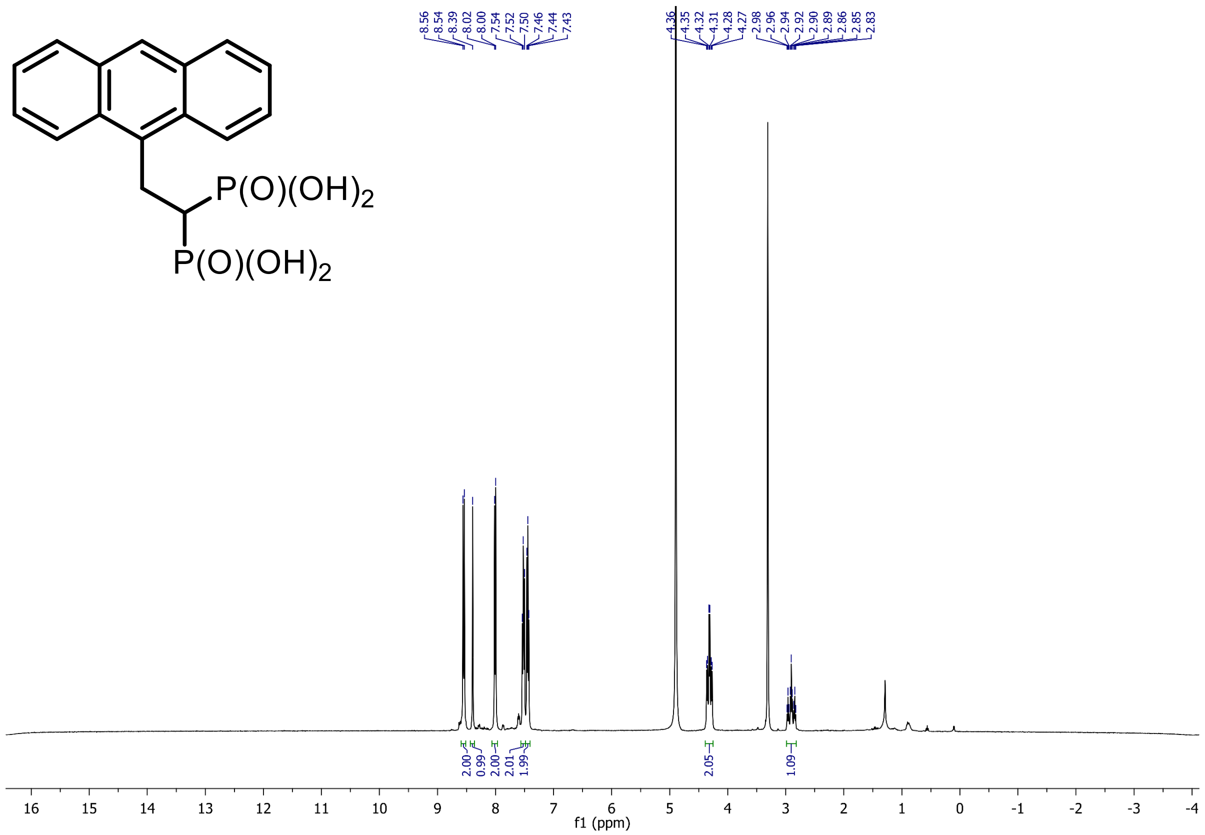

SI Figure 42. ^{1}H NMR (400 MHz, CD_3OD) of (2-(anthracen-9-yl)ethane-1,1-diyl)diphosphonic acid (**S9**).

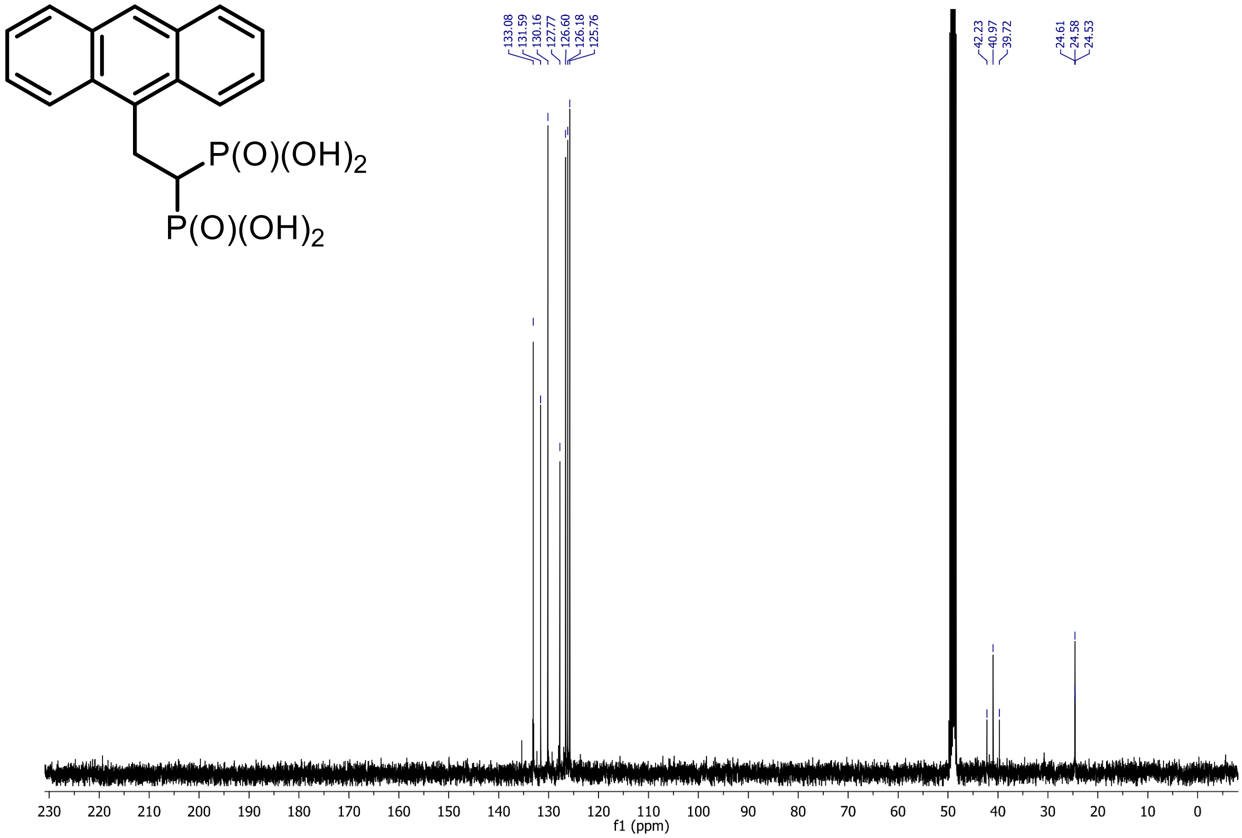

SI Figure 43. ^{13}C NMR (101 MHz, CD_3OD) of (2-(anthracen-9-yl)ethane-1,1-diyl)diphosphonic acid (**S9**).

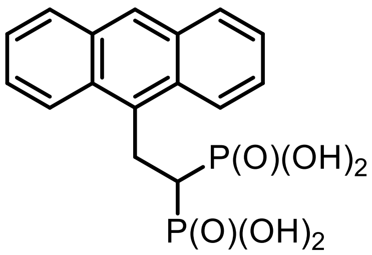

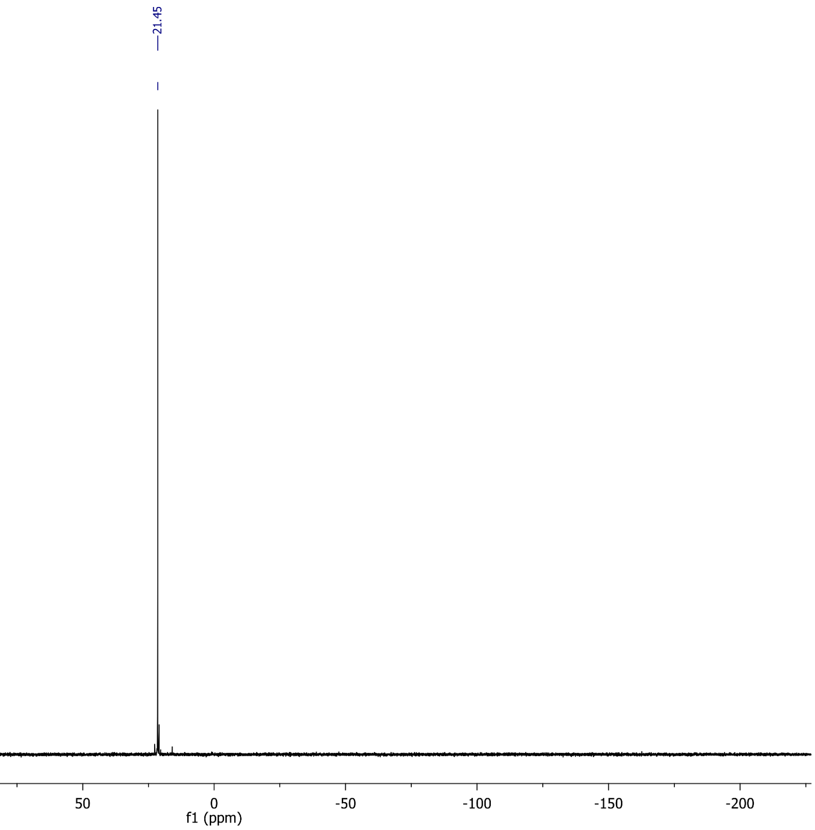

SI Figure 44. $^{31}P\{^{1}H\}$ NMR (162 MHz, CD_3OD) of (2-(anthracen-9-yl)ethane-1,1-diyl) diphosphonic acid (**S9**).

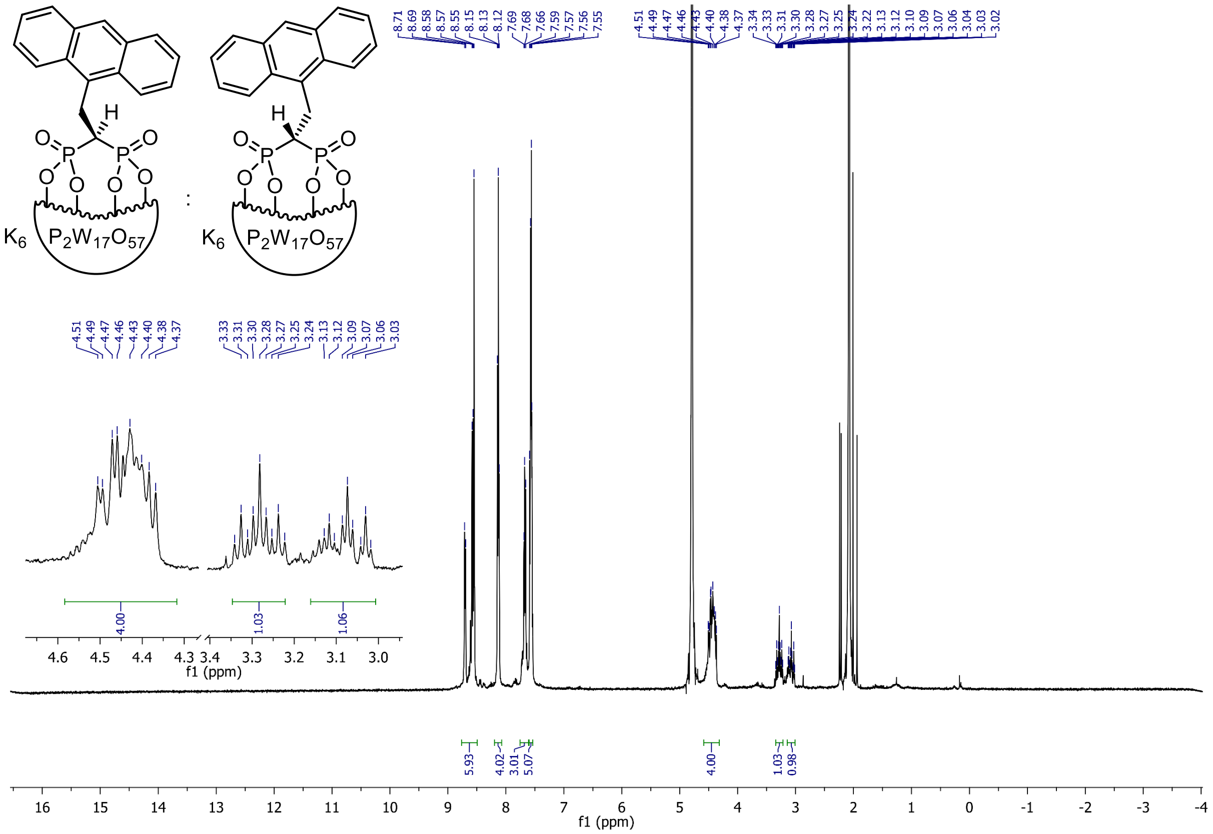

SI Figure 45. ^{1}H NMR (500 MHz, D_2O) of $K_6[P_2W_{17}O_{57}(P_2O_6C(H)CH_2$9-anthryl)] (**9**) as a 1:1 mixture of diastereomers.

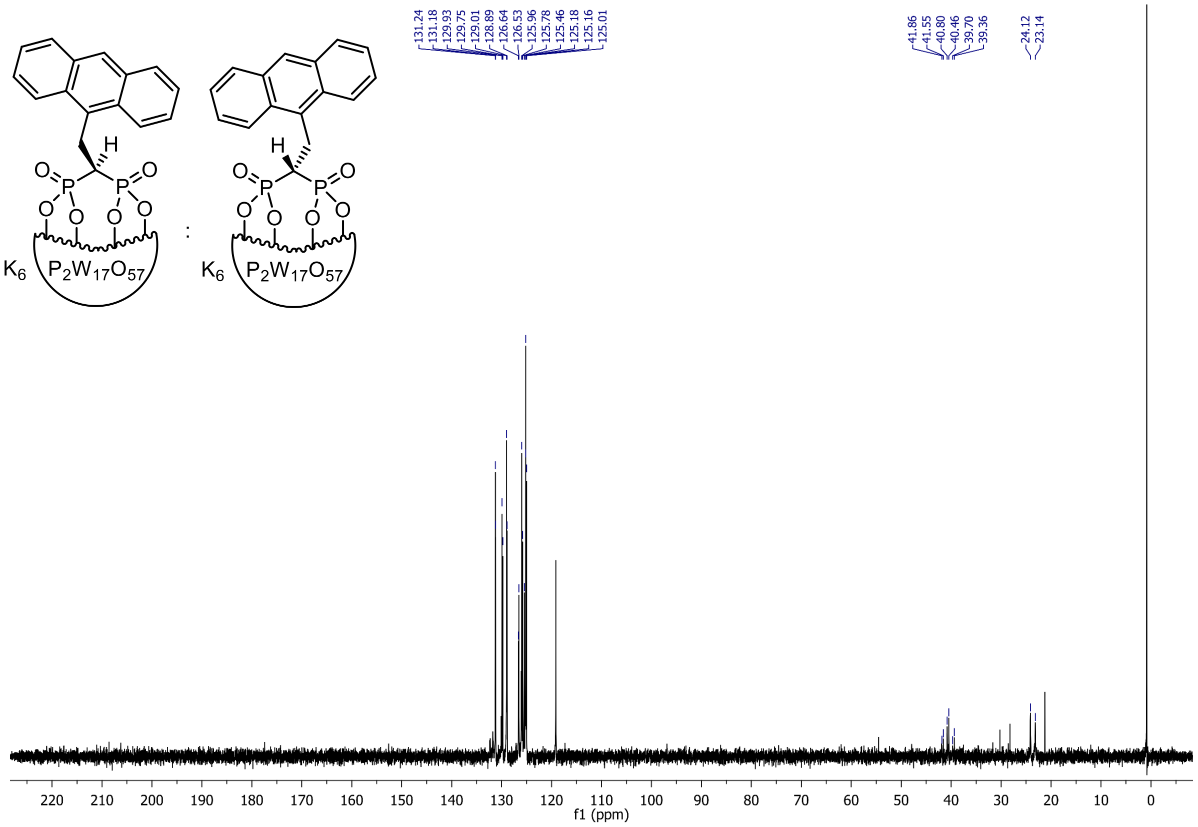

SI Figure 46. ^{13}C NMR (126 MHz, D_2O) of $K_6[P_2W_{17}O_{57}(P_2O_6C(H)CH_2$9-anthryl)] (**9**) as a 1:1 mixture of diastereomers.

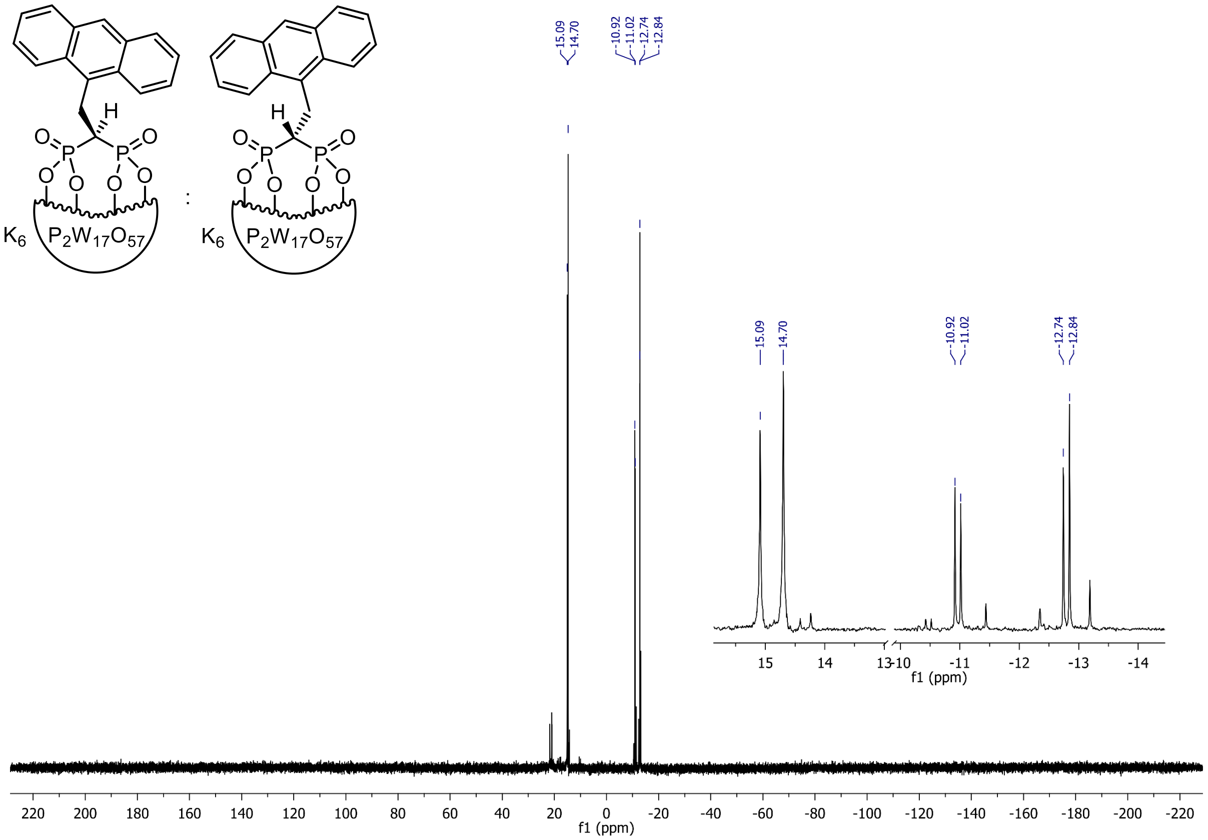

SI Figure 47. $^{31}P\{^{1}H\}$ NMR (202 MHz, D_2O) of $K_6[P_2W_{17}O_{57}(P_2O_6C(H)CH_29\text{-anthryl})]$ (**9**) as a 1:1 mixture of diastereomers.

3.2 Crystallography

Single crystals were selected and mounted using Fomblin® (YR-1800 perfluoropolyether oil) on a polymer-tipped MiTeGen MicroMount™ and cooled rapidly to 120 K in a stream of cold N_2 using an Oxford Cryosystems open flow cryostat.[15] Single crystal X-ray diffraction data were collected on an Oxford Diffraction SuperNova diffractometer (**3**; TitanS2 CCD area detector, mirror-monochromated Cu-Kα radiation source; λ = 1.54184 Å, **4′**; Atlas CCD area detector, mirror-monochromated Mo-Kα radiation source; λ = 0.71073 Å; ω scans). Cell parameters were refined from the observed positions of all strong reflections and absorption corrections were applied using a Gaussian numerical method with beam profile correction (CrysAlisPro).[16] Structures were solved within Olex2[17] by dual space iterative methods (SHELXT)[18] and all non-hydrogen atoms refined by full-matrix least-squares on all unique F2 values with anisotropic displacement parameters (SHELXL).[18] Hydrogen atoms were refined with constrained riding geometries and thermal parameters linked to Uiso of their parent atoms. Structures were checked with checkCIF.[19] CCDC-2239782-3 contains the supplementary data for these compounds. These data can be obtained free of charge from The Cambridge Crystallographic Data Centre via www.ccdc.cam.ac.uk/data_request/cif.

$K_6[P_2W_{17}O_{57}(P_2O_6NH)]$ (3)

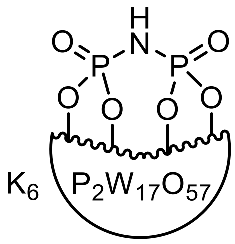

Crystals suitable for SC-XRD were prepared by vapour diffusion with acetonitrile and methanol as the antisolvent. CCDC identification Number: 2239782

The amide hydrogen atoms were not observed in the electron density map; they were geometrically placed and refined with a riding model. The anisotropic displacement parameter of oxygen atom O59A was restrained to have more isotopic character (ISOR). A solvent mask was calculated and 1073 electrons were found in a volume of 4421 Å^3 in 1 void per unit cell. This is consistent with the presence of 6[K], 35[H_2O] per Formula Unit which account for 1856 electrons per unit cell. The number of water residues was primarily estimated on the basis of the volume of the solvent masked region. The residual electron density peaks and holes are adjacent to W atoms and will be a result in deficiencies in the absorption correction of the data for this plate-like crystal. Measures taken to mitigate this problem include use of Mo radiation and application of both empirical and Gaussian absorption correction.

$(^nBu_4N)_6[P_2W_{17}O_{57}(P_2O_6CH_2)]$ (4′)

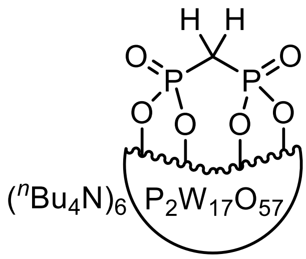

Crystals suitable for SC-XRD were prepared via vapour diffusion with DMF and ethyl acetate as the antisolvent. CCDC identification Number: 2239783

The crystal of the polyoxometalate contains large regions of diffuse counterions and solvent resulting in weak scattering at high angle. The data used in the refinement was truncated at a resolution of 0.95 Å. Extensive use of restraints and constraints were made to support refinement of a chemically sensible model with a low data to parameter ratio. The structure was refined as an inversion twin with the batch scale factor refining to a value of 0.22(2).

All atoms in the Dawson polyoxometalate anion were refined with anisotropic displacement parameters whilst all atoms of the surrounding cations and solvent residues were refined with isotropic displacement parameters. Rigid bond and similarity restraints (RIGU, SIMU) were applied to all atoms in the structure. The anisotropic displacement parameters of oxygen atoms O13, O103, O104 and O116 were restrained to have more isotropic character (ISOR). The anisotropic displacement parameters of close lying disorder component pair oxygen atoms O21/A and O22/A were constrained to have identical parameters (EADP). The carbon atoms involved in the butyl disorder in the tetrabutylammonium residue were refined with their isotropic parameters fixed at a value of 0.15.

Disorder is observed in the identity of the polyoxometalate anion residue with both the iso-metallic tungsten Dawson anion and lacunary hetero Dawson anion being present in the structure. The atoms of the two species overlap for the most part: only seven atoms of the lacunary species are refined distinctly from the shared body. The occupancies of the two components were refined and constrained to sum to unity giving values of 0.87(1) and 0.13(1) for the hetero-lacunary and iso-metallic species respectively. Disorder was also modelled in one of the butyl moieties of tetrabutylammonium residue B. The occupancies of the disorder components were likewise refined and constrained to sum to unity giving values of 0.73(4) and 0.27(4) respectively.

The 1,2 and 1,3 distances and angles of all atoms in the tetrabutylammonium residues were constrained to ideal target values taken from examples in the CSD (DFIX). All C-N bonds across the tetrabutylammonium residues were restrained to have similar values (SIMU).

The atoms of the two dimethylformamide residues were refined as rigid bodies with coordinates taken from the Olex2 fragment library. The C-N bond lengths in the dimethylammonium residue were restrained to a target value of 1.47 Å (DFIX).

The W-O bond lengths around the top and bottom three tungsten atoms of the iso- structure were restrained to be similar reflecting the idealised three-fold symmetry of the residue (SADI). The 1,2 and 1,3 distances pertaining to the lacunary residue were restrained to reflect the idealised mirror symmetry of the residue (SADI). The W-O bond length between W1 and O100 in the iso disorder residue was restrained to a target value of 1.7 Å to match the chemically equivalent bonds across the structure (DIFX). The P-C bond lengths of P104, C101, P102 and C101 in the lacunary structure were restrained to target value 1.79 Å, informed by equivalent bond lengths from structures in the CSD (DFIX).

A solvent mask was calculated, and 28 electrons were found in a volume of 5394 Å^3 in 1 void per unit cell. This is consistent with the presence of 2[K], 50[(H_2O)] per asymmetric unit which account for 3552 electrons per unit cell. The contents of the void were estimated on the basis of both charge balance and void volume, with the assumption that each disordered water molecule occupy a volume of 25 Å^3. The contents of the void region have been included in the calculation of the unit cell contents and all parameters derived therefrom.

Table S10. Crystallographic details for compounds **3** and **4′**.

	**3**	**4′**
CCDC Deposit	2239782	2239783
Chemical formula	$HNO_{63}P_4W_{17}$	$C_{0.866}H_{1.731}O_{62.731}P_{3.731}W_{17.135}\cdot 3(C_{16}H_{36}N)\cdot 0.734(C_2H_8N)\cdot 2(C_3H_7NO)\cdot 2[K]\cdot 50[(H_2O)]$
M_r	5137.50	6913.58
Crystal system, space group	Triclinic, $P1$	Orthorhombic, $P2_12_12_1$
Temperature (K)	120	120
a, b, c (Å)	13.7612 (2), 23.2229 (3), 27.6812 (3)	18.2336 (7), 26.9481 (7), 31.5198 (7)
α, β, γ (°)	98.146 (1), 94.634 (1), 93.986 (1)	90, 90, 90
V (Å^3)	8698.9 (2)	15487.6 (8)
Z (Z')	4 (2)	4 (1)
Radiation type	Cu $K\alpha$	Mo $K\alpha$
μ (mm^{-1})	44.79	12.91
Crystal size (mm)	0.15 × 0.1 × 0.03	0.09 × 0.04 × 0.04
Diffractometer	SuperNova, Titan S2	SuperNova, Atlas
T_{min}, T_{max}	0.028, 0.425	0.477, 0.763
No. of measured, independent and observed [$I > 2\sigma(I)$] reflections	99190, 32788, 29136	132761, 18877, 14701
R_{int}	0.082	0.096
θ_{max} (°)	70.8	22.0
$(\sin \theta/\lambda)_{max}$ (Å^{-1})	0.612	0.526
$R[F^2 > 2\sigma(F^2)]$, $wR(F^2)$, S	0.074, 0.203, 1.06	0.053, 0.138, 1.06
No. of reflections	32788	18877
No. of parameters	1531	1034
No. of restraints	6	2277
	$w = 1/[\sigma^2(F_o^2) + (0.1139P)^2 + 137.5666P]$ where $P = (F_o^2 + 2F_c^2)/3$	$w = 1/[\sigma^2(F_o^2) + (0.0611P)^2 + 80.1977P]$ where $P = (F_o^2 + 2F_c^2)/3$
$\Delta\rangle_{max}$, $\Delta\rangle_{min}$ (e Å^{-3})	5.88, -3.27	1.73, -0.92
Absolute structure	–	Refined as an inversion twin.
Absolute structure parameter	–	0.22 (2)

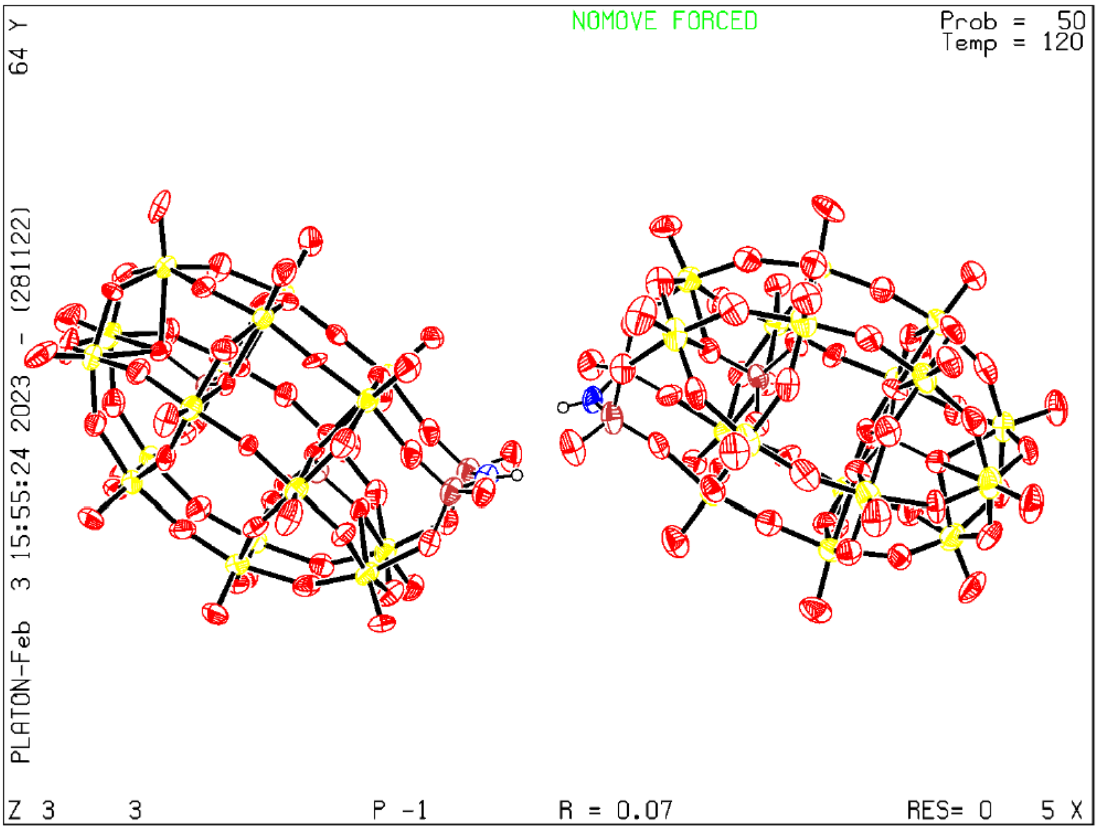

SI Figure 48. ORTEP diagram of POM **3**.

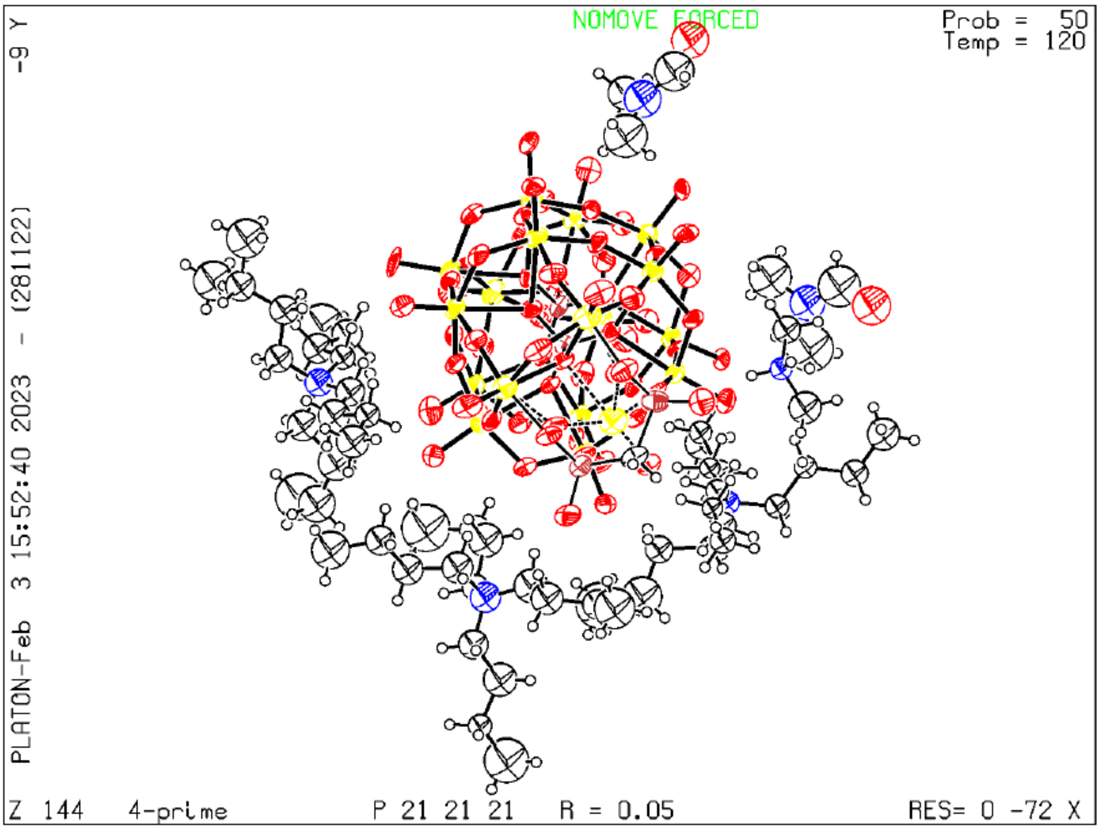

SI Figure 49. ORTEP diagram of POM **4′**.

4.0 References

[1] W. L. F. Armarego, C. Chai, Armarego, *Purification of Laboratory Chemicals*, Jordan Hill: Elsevier Science & Technology, Jordan Hill, **2009**.

[2] C. R. Graham, R. G. Finke, *Inorg. Chem.* **2008**, *47*, 3679-3686.

[3] N. A. Boyle, *Org. Lett.* **2006**, *8*, 187-189.

[4] A. R. H. Dougourikoye, R. Babouri, J.-N. Volle, A. T. Ilagouma, J.-L. Pirat, D. Virieux, *ARKIVOC* **2020**, *2020*, 168-180.

[5] K. Hossain, L. Florean, A. Del Tedesco, E. Cattaruzza, M. Geppi, S. Borsacchi, P. Canton, A. Benedetti, P. Riello, A. Scarso, *Chem. Eur. J.* **2021**, *27*, 17941-17951.

[6] S. S. Amin, J. M. Cameron, M. Winslow, E. S. Davies, S. P. Argent, D. Robinson, G. N. Newton, *Eur. J. Inorg. Chem* **2022**, *2022*, e202200019.

[7] G. R. Fulmer, A. J. M. Miller, N. H. Sherden, H. E. Gottlieb, A. Nudelman, B. M. Stoltz, J. E. Bercaw, K. I. Goldberg, *Organometallics* **2010**, *29*, 2176-2179.

[8] R. K. Harris, E. D. Becker, S. M. C. d. Menezes, P. Granger, R. E. Hoffman, K. W. Zilm, *Pure and Applied Chemistry* **2008**, *80*, 59-84.

[9] R. D. Chambers, J. Hutchinson, *Journal of Fluorine Chemistry* **1998**, *92*, 45-52.

[10] G. Bianchini, A. Scarso, A. Chiminazzo, L. Sperni, G. Strukul, *Green Chemistry* **2013**, *15*, 656-662.

[11] G. S. Kim, K. S. Hagen, C. L. Hill, *Inorg. Chem.* **1992**, *31*, 5316-5324.

[12] S. Fujimoto, J. M. Cameron, R. J. Wei, K. Kastner, D. Robinson, V. Sans, G. N. Newton, H. Oshio, *Inorg. Chem.* **2017**, *56*, 12169-12177.

[13] M. H. Anjass, K. Kastner, F. Ngele, M. Ringenberg, J. F. Boas, J. Zhang, A. M. Bond, T. Jacob, and C. Streb, *Angew. Chem. Int. Ed.* **2017**, *56*, 14749-14752.

[14] A. A. Isse and A. Gennaro, *J. Phys. Chem. B* **2010**, 114, 7894–7899.

[15] J. Cosier and A. M. Glazer, *J. Appl. Crystallogr.*, **1986**, *19*, 105-107.

[16] Rigaku Oxford Diffraction, (2018), CrysAlisPro Software system, version 1.171.40.45a, Rigaku Corporation, Oxford, UK.

[17] O. V Dolomanov, L. J. Bourhis, R. J. Gildea, J. A. K. Howard, H. Puschmann, *J. Appl. Cryst.* **2009**, *42*, 339–341.

[18] G. M. Sheldrick, *Acta Crystallogr. A* **2015**, *71*, 3–8.

[19] "CheckCIF," can be found under http://checkcif.iucr.org
